# Dietary high salt drives cognitive impairment and Aβ accumulation via an insufficient compensatory mitophagy response

**DOI:** 10.3389/fneur.2026.1922997

**Published:** 2026-10-26

**Authors:** Jie Wen, Hao Ren, Fan Wen, Junping Yu, Haixia Fan, Jincheng Liu, Zhiyou Cai

**Affiliations:** 1 Department of Neurology, Xiangyang Hospital of Traditional Chinese Medicine, Xiangyang Hospital affiliated to Hubei University of Chinese Medicine, Xiangyang, Hubei, China; 2 Department of Medical Administration, First Hospital of Shanxi Medical University, Taiyuan, Shanxi, China; 3 Department of Neurology, Liangjiang Hospital of Chongqing Medical University, People Hospital of Chongqing Liangjiang New Area, Chongqing, China

**Keywords:** amyloid-β, behavior, cognitive impairment, high-salt diet, mitophagy

## Abstract

There is mounting evidence that dietary habits are strongly correlated with cerebrovascular diseases and dementia, but the precise mechanism remains unclear how a salt-rich diet adversely affects the brain. Here, 12-month-old mice were fed a high-salt diet (HSD) for 2 months, and we observed salt-induced cognitive deficits in mice. The short-term HSD increased mitophagy-associated markers (PINK1, Parkin, and LC3-II) and decreased SQSTM1/p62, consistent with a compensatory mitophagy response that was insufficient to prevent cognitive decline. Neuro-2a cells were cultured in normal medium, NaCl medium (40 mM) or NaCl (40 mM) + CCCP group (20 μM) medium for 24 h, respectively. Short-term HSD feeding impaired learning and memory and increased amyloid-β (Aβ) burden, in parallel with altered PINK1/Parkin-associated mitophagy markers. These findings link a salt-rich diet to cognitive dysfunction, and suggest that enhancing PINK1/Parkin-mediated mitophagy may counteract the harmful effects of HSD on the brain. Therefore, the PINK1/Parkin mitophagy may be a potential target for combating the harmful effects on the brain caused by HSD or other diseases related to mitophagy.

## 1 Introduction

Excessive sodium chloride (NaCl) intake is a significant global health concern, strongly associated with diseases including hypertension and neurodegeneration, especially if sodium intake exceeds 5 g/day (1). While the detrimental effects of a high-salt diet (HSD) are recognized, the specific mechanisms linking dietary salt to neurological pathology, particularly Alzheimer’s disease (AD), remain incompletely understood. Potential pathways include interference with neurotransmitters, disruption of brain energy metabolism, promotion of inflammation and oxidative stress, and alterations in mitochondrial function and gut microbiome composition, which may influence neurodegeneration via the gut-brain axis.

Mitochondria are critical for neuronal energy supply via ATP production. Mammalian cells possess comprehensive quality control mechanisms, an autophagosome-lysosome system (2), to identify and eliminate damaged mitochondria, such as PINK1-PRKN mediated mitophagy. In this pathway, PINK1 (PTEN-induced kinase 1) accumulates on depolarized mitochondria, recruits the E3 ubiquitin ligase PRKN (Parkin), and targets damaged mitochondria for degradation via autophagosomes. Significantly, mutations in PINK1 and PRKN cause familial Parkinson’s disease, underscoring the importance of mitophagy in neuronal health (3). Impaired mitophagy is increasingly implicated in AD pathogenesis. PINK1 expression was significantly decreased in the brains of AD patients or APP transgenic mice than in normal control or wild-type mice, revealing that impaired mitophagy may be related to AD (4). Amyloid-β (Aβ) deposition and insoluble tau aggregation are considered to be responsible for neuronal dysfunction and cognitive deficits in AD patients. Impaired mitophagy results in the accumulation of dysfunctional mitochondria, which contributes directly to Aβ peptide generation (5, 6) and aggregation key pathological features of AD characterized by mitochondrial dysfunction. Furthermore, it has also been shown that overexpression of mitophagy-related proteins in AD can preserve hippocampal mitochondrial function by improving the level of mitophagy to lower APP and Aβ (4). Aggregation of Aβ peptides in AD is characterized by mitochondrial dysfunction and impaired mitophagy. Notably, HSD feeding induces AD-like pathology, including increased Aβ accumulation and tau hyperphosphorylation, and exacerbates pathology in AD model mice (7).

Previous research has found that an HSD disrupts mitochondrial respiration via the electron transport chain. Furthermore, the amount of sodium consumed affects the immune and functional processes of human cells. Data shows that HSD intake directly affects the respiratory activity of monocyte mitochondria, which may affect their activation and function. Changes in intracellular sodium levels may also greatly affect the oxidative phosphorylation and function of other cell types, which are also affected by dietary sodium changes (8). In addition, long-term exposure to an environment rich in sodium chloride increases the level of reactive oxygen species (ROS), which affects endothelial cell mitosis (9). All of the above researches suggest that a HSD disrupts mitochondrial homeostasis, which plays an indispensable role in cellular metabolism.

Despite the established links between HSD, AD-like pathology, and impaired mitophagy in AD, the specific role of mitophagy in mediating HSD-induced Aβ accumulation and cognitive decline remains unexplored. Based on the evidence of HSD-induced neurodegeneration and the critical role of mitophagy in mitochondrial quality control and Aβ regulation, we hypothesize that HSD-induced mitochondrial injury triggers a compensatory but insufficient activation of PINK1/Parkin-mediated mitophagy; the resulting accumulation of dysfunctional mitochondria promotes Aβ production and, ultimately, cognitive decline. Enhancing PINK1/Parkin-mediated mitophagy may therefore counteract these deleterious effects. This compensatory insufficiency, rather than mitophagy per se, may constitute a pathophysiological mechanism linking HSD to Aβ accumulation and cognitive decline.

## 2 Materials and methods

### 2.1 Mice

The male C57BL/6J mice (ENSIWEIER, Chongqing, China) were housed in 12 h light/dark cycle and a temperature-controlled facility at the Laboratory Animal Center of the Chongqing Key Laboratory of Neurodegenerative Diseases (Chongqing, China). All procedures were approved by the committee of Chongqing Medical University. Mice were randomly allocated to the ND or HSD group (*n* = 10 per group), and all behavioral tests and image analyses were performed by investigators blinded to group allocation.

### 2.2 High-salt diet

Mice (12 months old) were given normal chow (0.5% NaCl, normal diet, ND) or sodium-rich chow (8% NaCl, high salt diet, HSD) (Tengxin Biotechnology Co., Ltd., Chongqing, China) for 2 months. All mice had tap water ad libitum. Thereafter, 14-month-old C57BL/6 mice were used to evaluate the interaction between cognitive and HSD.

### 2.3 Cell culture and processing

The mouse neuroblastoma N2a cells (Procell Life Science&Technology Co., Ltd) were maintained in Dulbecco’s Modified Eagle Medium (DMEM, Gibco, Carlsbad, CA, USA). All cell cultures were complemented with 10% fetal bovine serum (FBS, Invigentech, Irvine, CA, USA) and antibiotics (100 U/mL penicillin and 100 μg/mL streptomycin). After a 24-h differentiation period, cells were divided into three groups: control (CON); NaCl (40 mM for 24 h); and NaCl (40 mM) plus CCCP (20 μM for 24 h) (10). Nontreated N2a cells were control (CON) group; exposed to NaCl (40 mM added to DMEM, yielding a total NaCl concentration of ~150 mM, approximating physiological extracellular sodium levels) (NaCl group). CCCP (carbonyl cyanide 3-chlorophenylhydrazone) is a protonophore and the most widely used pharmacological inducer of PINK1/Parkin-mediated mitophagy. The 20 μM concentration and 24 h exposure duration applied here fall within the range that has been validated in neuronal and non-neuronal cell lines to activate PINK1/Parkin-mediated mitophagy without overt cytotoxicity at the time points used for downstream readouts (3, 10, 11). The NaCl working concentration (40 mM) was chosen because it did not significantly affect N2a cell viability (Figure 1B). The combined NaCl + CCCP condition was designed to test whether pharmacological enhancement of mitophagy could attenuate NaCl-induced Aβ elevation; the absence of a separate CCCP-only group is acknowledged as a limitation (see Discussion).

**Figure 1:**
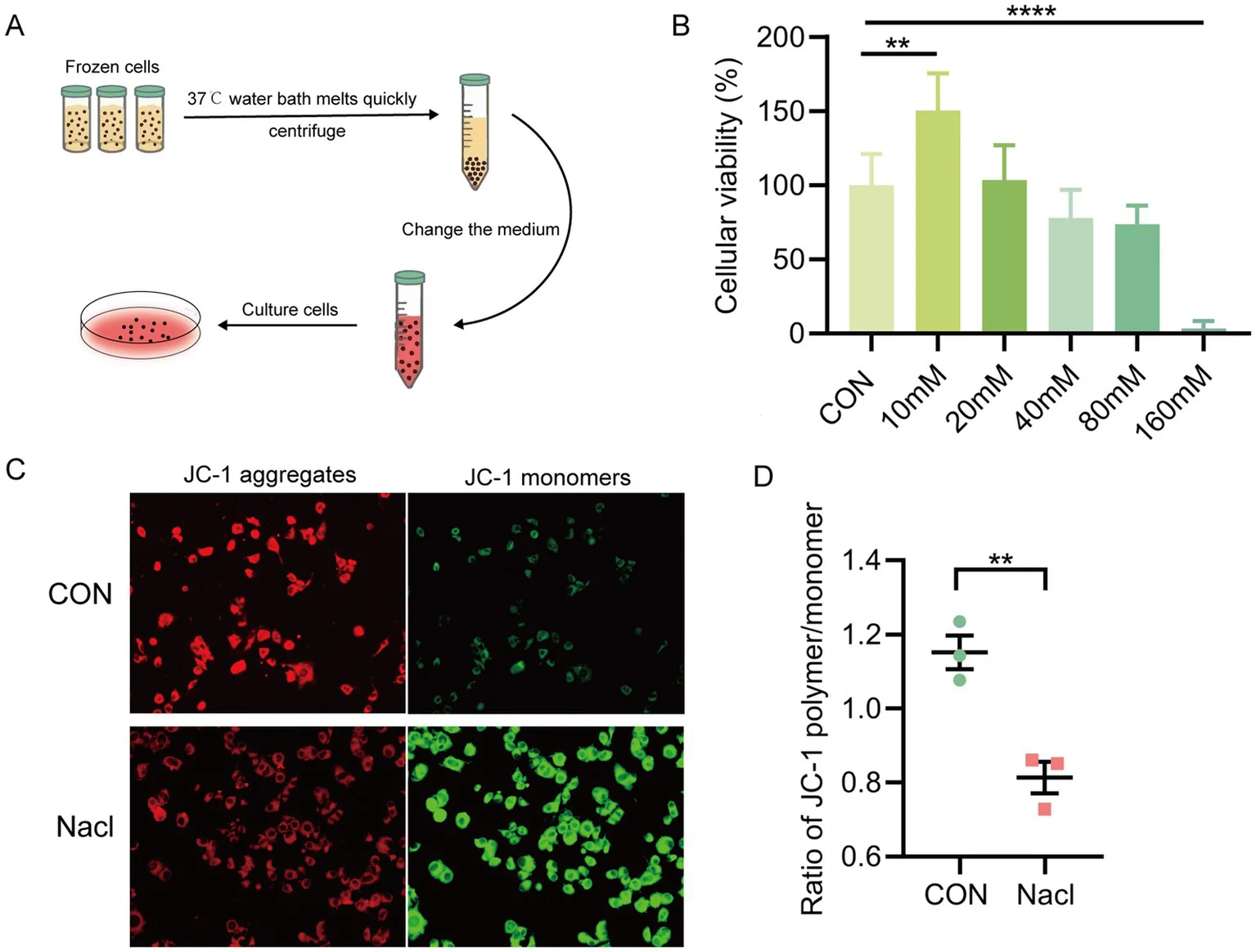
High salt induces mitochondrial damage. **(A)** Representative images of cultured N2a cells. **(B)** Cell viability under different NaCl concentrations. **(C)** JC-1 staining showing mitochondrial membrane potential: red fluorescence indicates JC-1 aggregates, while green fluorescence reflects the monomeric form. **(D)** Quantification of red/green fluorescence ratios to assess mitochondrial depolarization (*n* = 3 per group; two-sample *t*-test). Scale bar, 20 μm. Data are presented as mean ± SEM; **p < 0.01, ****p < 0.0001. N2a, Neuro-2a; CON, control.

### 2.4 Mitochondrial membrane potentials assay

Mitochondrial depolarization was assessed using a JC-1 probe (C2003S, Beyotime Biotechnology, Shanghai, China). Cells were seeded in 6-well plates and incubated with 1 mL of JC-1 working solution at 37 °C for 20 min, followed by two washes with PBS. Fluorescence was then examined under a microscope. At high mitochondrial membrane potential, JC-1 aggregates in the mitochondrial matrix and emits red fluorescence, whereas a reduction in membrane potential retains JC-1 in its monomeric form, producing green fluorescence in the cytoplasm. Thus, alterations in membrane potential can be evaluated by shifts in fluorescence color, with the red/green fluorescence ratio serving as a standard indicator of mitochondrial depolarization.

### 2.5 Behavioral tests

#### 2.5.1 Morris water maze test

The Morris water maze (MWM) was used to assess spatial learning and memory in mice after 2 months of HSD feeding. A hidden platform (8 cm in diameter) was placed in the northeast quadrant of a circular pool (1.2 m diameter, 0.4 m depth); water temperature was maintained at 25 °C ± 1 °C. Acclimated mice underwent four training trials per day for five consecutive days. At the start of each trial, mice were released from one of four designated start positions and allowed to swim freely for up to 60 s. Upon reaching the platform, mice were permitted to remain on it for 5 s. If a mouse failed to locate the platform within 60 s, it was gently guided to the platform and allowed an additional 30 s. At the end of each trial, mice were removed from the pool, gently dried, and returned to their home cage. Escape latency and swim trajectories were recorded using ANY-maze video-tracking software (Stoelting Co., USA). On day 6 (probe trial), the platform was removed and mice were released from the quadrant opposite the former platform location; during a 60-s probe, time spent in the target quadrant, distance traveled within the target quadrant, and the number of crossings of the former platform location were recorded (12).

#### 2.5.2 Open field test

Spontaneous locomotor activity and anxiety-like behavior were assessed in an open field arena. Each mouse was placed in the center of the arena and allowed to explore freely for 3 min; total distance travelled, the number of entries into, distance travelled within, and time spent in the central zone were recorded and analyzed with ANY-maze video-tracking software (Stoelting Co., USA). The arena was cleaned with 75% ethanol between animals.

#### 2.5.3 Shuttle-box test

Active avoidance learning was assessed using a two-way shuttle box. Each session consisted of 30 trials per day on 5 consecutive days. In each trial, a conditioned stimulus (light) was presented for 5 s, followed by an unconditioned stimulus (electric foot shock, 0.4 mA) delivered through the grid floor; a shuttling response to the opposite compartment during the conditioned stimulus terminated the trial and was scored as an active avoidance. The number of active-avoidance failures per session was recorded and used as the index of learning performance. Detailed parameters of the behavioral protocols have been described previously.

### 2.6 Western blot analysis

Brain tissues were dissected to isolate the cortex and hippocampus. Total protein was extracted and quantified using a BCA protein assay kit (Beyotime Biotechnology, Shanghai, China). Equal amounts of protein were separated by SDS-PAGE on 10%–15% gels alongside a prestained protein molecular-weight marker (e.g., PageRuler, Thermo Scientific) and transferred onto polyvinylidene fluoride membranes (PVDF; Bio-Rad, Hercules, CA, USA). After blocking, membranes were incubated overnight at 4 °C with the following primary antibodies: anti-PINK1 (#29297, SAB), Parkin (#4211, CST), LC3A/B (#12741, CST), SQSTM1/p62 (#23214, CST), beclin1 (#3495, CST), Neprilysin (18008-1-AP, Proteintech Group, Inc), Presenilin1 (#5643, CST), Presenilin 2/AD5 (ab51249, Abcam), BACE (#5606, CST), Aβ (#8243, CST), Aβ (1–42) (#14974, CST), glyceraldehyde-3-phosphate dehydrogenase (GAPDH, AF0006, Beyotime) at 4 °C overnight. The membranes were then incubated with HRP-conjugated secondary antibodies for 1 h at room temperature. Protein bands were visualized using Immobilon Western chemiluminescent HRP substrate (Millipore, Billerica, MA, USA) and imaged with the Tanon-5200 multiplex chemiluminescent analysis system (Tanon, Shanghai, China). Band intensities were quantified with ImageJ and normalized to GAPDH as indicated. Each experiment was performed with three independent biological replicates.

### 2.7 Immunofluorescence staining

Whole brains were collected and fixed in 4% paraformaldehyde for 48 h. Tissues were embedded in paraffin and sectioned at 2 μm in the sagittal plane using a rotary microtome (HM 340E, Thermo Scientific). Sections were blocked with 5% goat serum and incubated overnight at 4 °C with anti-Aβ antibody (#8243, CST). After washing, sections were incubated for 1 h in the dark with Alexa Fluor 488-conjugated secondary antibody (ZSGB-BIO, Beijing, China), followed by nuclear counterstaining with DAPI (Beyotime) at 37 °C for 8 min. Images were acquired using a NEXCOPE fluorescence microscope (NE900, USA) (12). For quantification, three coronal sections per animal and five randomly selected fields per section were analyzed (*n* = 3 animals per group); fluorescence intensities from multiple fields of the same animal were averaged and treated as one biological replicate.

### 2.8 Transmission electron microscopy

The hippocampal tissue was pre-fixed in 3% glutaraldehyde and post-fixed in 1% osmium tetroxide, followed by dehydration in a graded acetone series (50%, 70%, 80%, 90%, and 95%), and finally in 100% acetone three times. Samples were infiltrated and embedded in Epox 812. Semi-thin sections were stained with methylene blue, and ultrathin sections (60–90 nm) were obtained using an ultramicrotome. Sections were subsequently stained with uranyl acetate for 10–15 min and lead citrate for 1–2 min at room temperature. Ultrastructural analysis was performed using a JEM-1400-FLASH transmission electron microscope at magnifications of ×12,000 and ×30,000; scale bars are indicated in the figures. For morphological assessment, mitochondria were classified as normal, swollen, or containing autophagic structures according to pre-defined criteria, and autophagosome/autolysosome-like structures were identified on the basis of a double membrane or partially degraded contents. All ultrastructural evaluations were performed by an investigator blinded to group allocation.

### 2.9 Statistics

All data are presented as mean ± SEM. Statistical analyses were performed using GraphPad Prism (version 11.1.0, GraphPad Software, San Diego, CA, USA). The normality of the data was assessed using the Shapiro–Wilk test. Comparisons between two groups were performed using an unpaired two-tailed Student’s *t*-test, and comparisons among multiple groups were performed using one-way analysis of variance (ANOVA) followed by Tukey’s *post hoc* test. For behavioral data acquired repeatedly from the same animals over consecutive training days (escape latency in the Morris water maze and the number of active-avoidance failures in the shuttle-box test), a two-way repeated-measures ANOVA was applied to evaluate the effects of diet, time, and diet × time interaction, followed by Sidak’s multiple-comparisons test for between-group comparisons at individual time points. *p* < 0.05 was considered statistically significant (*p < 0.05, **p < 0.01, ***p < 0.001, and ****p < 0.0001).

## 3 Results

### 3.1 Dietary salt aggravated cognitive impairment

During the 2-month feeding, we found that mice fed HSD (8% NaCl) showed yellow, sparse hair compared with mice fed a ND (0.5% NaCl) (Figures 2A,B). As mice were short-term fed an HSD, the weight had no significant changed (Figure 2C). However, there was no significant in food intake (Figure 2D). The salt intake and water intake of mice on HSD was significantly higher than that of the normal group (Figures 2E,F).

**Figure 2:**
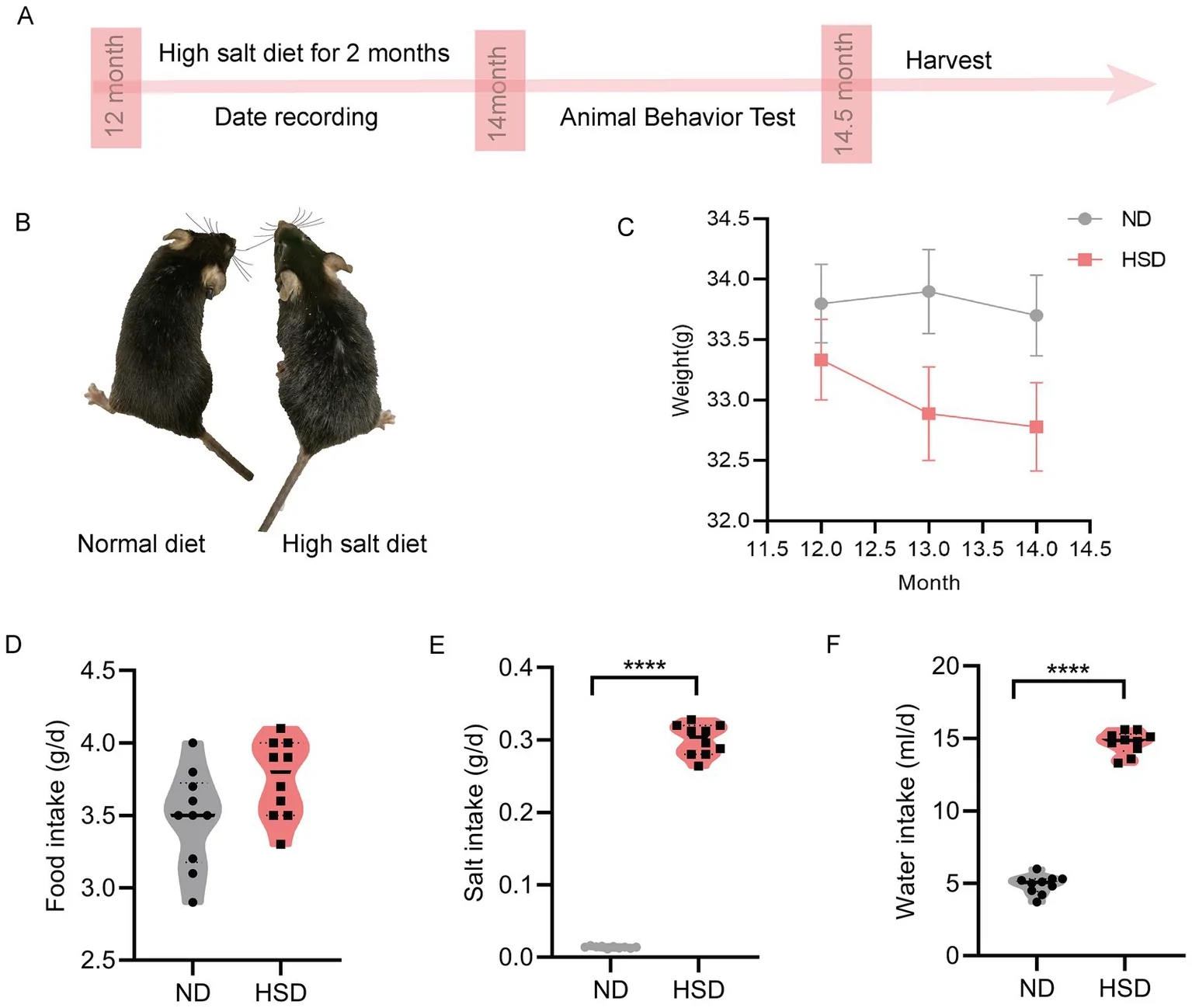
Characteristics of mice. **(A)** Twelve-month-old C57BL/6 mice were fed an HSD or a ND for 2 months. **(B)** Representative appearance of HSD- and ND-fed mice. **(C)** Body weight and **(D)** food intake did not differ significantly between groups. **(E,F)** HSD-fed mice consumed substantially more salt and water than ND-fed controls. *n* = 10 per group; two-sample *t*-test; mean ± SEM; *p < 0.05, ****p < 0.0001. HSD, high-salt diet; ND, normal diet.

Behavioral tests were performed on 14-month-old C57BL/6 mice (Figure 3A), including Morris water maze (MWM), two-way shuttle box test (SBT), and open field test (OFT), on each group of mice in turn. We found in MWM results that the HSD group still spent significantly more time to find the platform as the training time increases (days 4 and 5) (Figures 3B,C). Number of platform entries, time on platform, distance travelled to platform and time in target quadrant were significantly different between the two groups (Figures 3D–G). However, the mice did not differ in how fast they swam between the two groups, suggesting that the possibility that the difference between two groups was not related to the difference in swimming ability (Figure 3H). Figure 3I illustrates the trajectories of HSD and ND mice during the space exploration mission. In SBT, the number of active avoidance failures in HSD mice was significantly higher than that in the normal diet group (Figures 3J,K). Together, these data suggest HSD aggravated the process of cognitive deficits and spatial memory in aged C57BL/6 mice.

**Figure 3:**
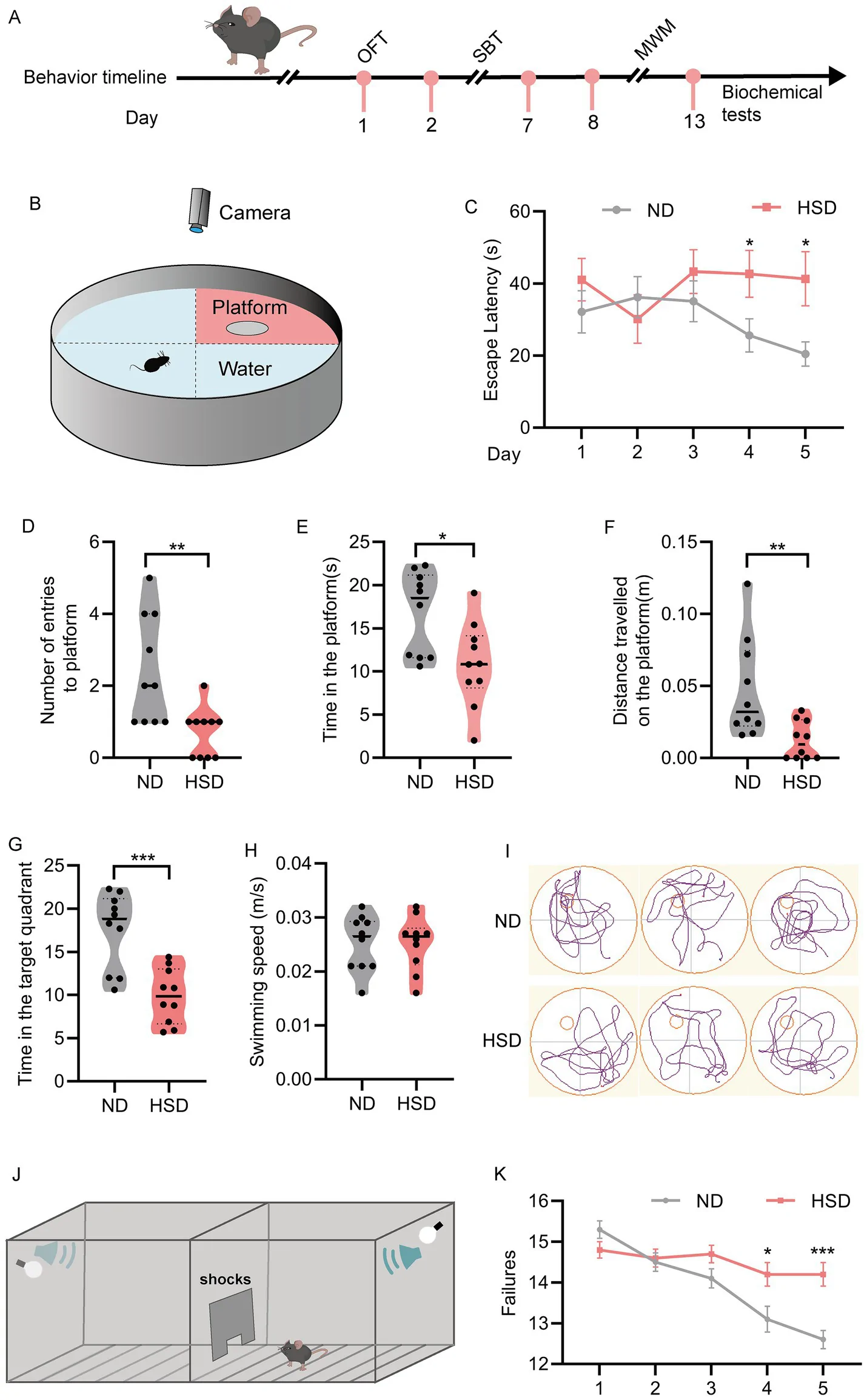
Dietary salts supplementation accelerates recognition deficits. Spatial learning and memory were assessed using the Morris water maze **(B)**. **(C)** Escape latency during training was recorded over consecutive days (*n* = 10 per group; repeated-measures two-way ANOVA; mean ± SEM; *p < 0.05). **(D–F)** In the probe trial, the number of platform crossings, time spent, and distance traveled in the target quadrant were analyzed. **(G,H)** Time spent in the target quadrant and swimming speed were further compared between groups. **(I)** Representative swimming trajectories on the fifth training day. **(J)** Schematic of the two-way shuttle box test (SBT). **(K)** The number of active avoidance failures during 5 consecutive training days. Data are presented as mean ± SEM; *n* = 10 per group; repeated-measures two-way ANOVA; *p < 0.05, **p < 0.01, ***p < 0.001. HSD, high-salt diet; ND, normal diet.

In the OFT, no significant between-group differences were detected in total distance travelled or in the number of center entries, distance travelled in the center, or time spent in the center (Figures 4A–D), indicating that spontaneous locomotor and exploratory behavior did not differ between groups; representative trajectories are shown in Figure 4E.

**Figure 4:**
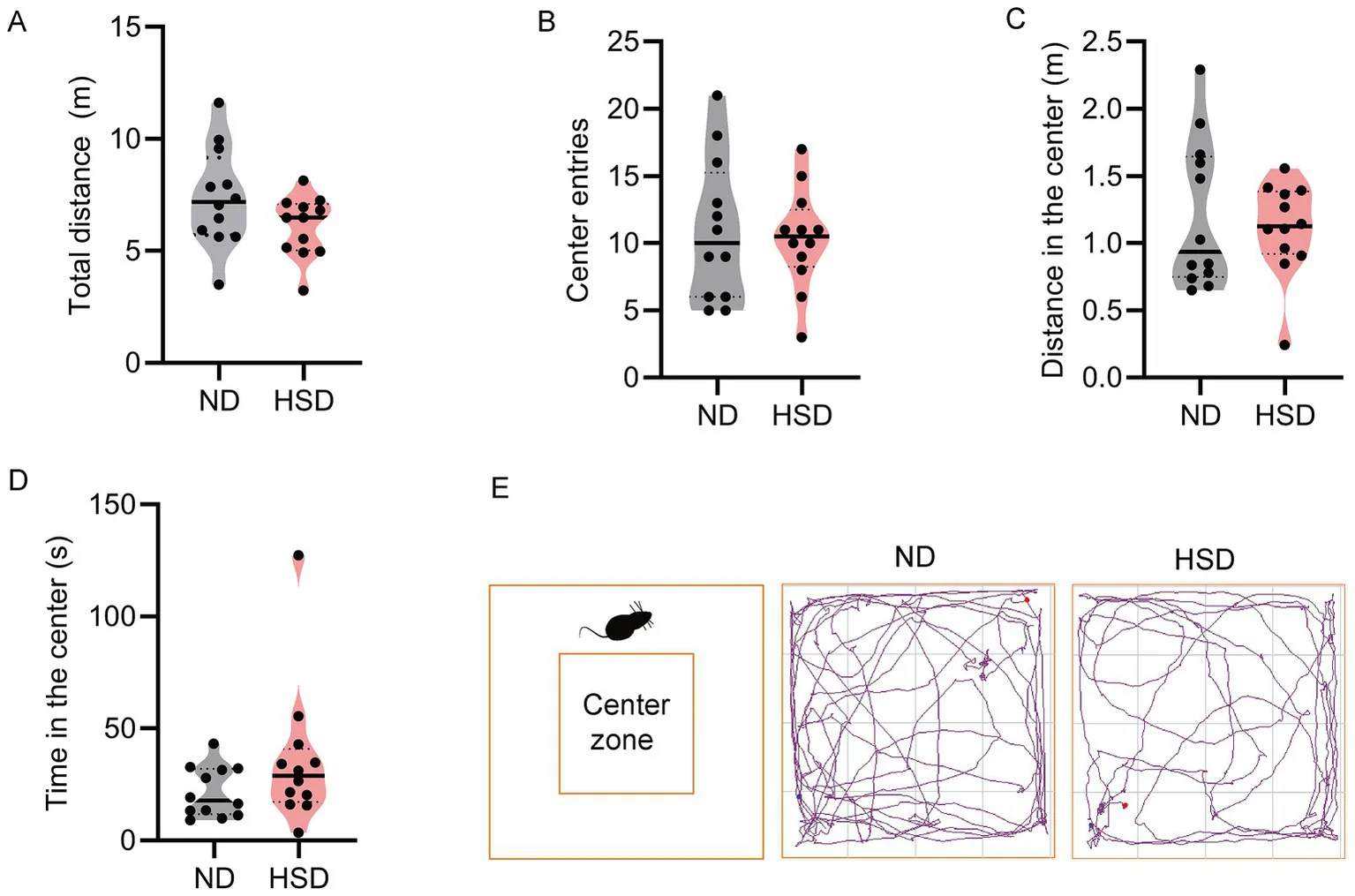
Dietary salt does not affect the anxiety-like behavior. 12-month-old mice were subjected to an open field test after 2 months of HSD treatment. Locomotor activity and anxiety-related parameters, including total distance traveled **(A)**, number of center entries **(B)**, distance traveled in the center **(C)**, and time spent in the center **(D)**, were quantified. Representative trajectories for each group are shown **(E)**, with the arena divided into central (four inner squares), peripheral (two-edge squares), and corner zones (four corner squares). Data are expressed as mean ± SEM. (*n* = 10/group; two-sample *t*-test). HSD, high-salt diet; ND, normal diet.

### 3.2 High salt induces mitochondrial damage and increases mitophagy-associated markers

First, we determined the appropriate concentration of NaCl-treated cells. Figure 1A showed the cell processing process. Compared with the control group, 160 mM NaCl significantly inhibited the viability of N2a (****p < 0.0001), while 40 mmol/L NaCl had no significant effect on the activity of N2A cells (*p* > 0.05). Therefore, we selected 40 mM, the concentration used in the mechanistic experiments (Figures 1, 5–7), as the working concentration of NaCl (Figure 1B). As shown in Figures 1C,D, changes in mitochondrial membrane potential were assessed using the JC-1 assay (Enhanced Mitochondrial Membrane Potential Assay Kit). A shift in fluorescence from red to green indicates a loss of membrane potential, which is a hallmark of early apoptosis. During mitophagy, the proteins Parkin, SQSTM1/p62, and LC3 are translocated from the cytoplasm to the mitochondrial membrane. HSD significantly increased the abundance of PINK1, Parkin, and LC3-II (normalized to GAPDH) and reduced SQSTM1/p62, whereas Beclin-1 levels were unchanged (Figures 5A,B). Because LC3-II immunoblotting alone cannot distinguish enhanced autophagosome formation from impaired autophagosome clearance (13), we interpreted this pattern together with the degradation of the autophagic substrate SQSTM1/p62: the coordinate increase in LC3-II and decrease in SQSTM1/p62 in HSD-fed mice is consistent with increased autophagic flux, although flux was not measured directly *in vivo*. Our *in vitro* model of an HSD was in line with the *in vivo* results. Our data indicate that HSD increased mitophagy-associated markers, consistent with a compensatory activation of PINK1/Parkin-mediated mitophagy; however, static markers alone cannot establish mitophagic flux (14). Moreover, co-treatment with CCCP (20 μM), a mitochondrial uncoupler widely used to induce PINK1/Parkin-mediated mitophagy (10), further increased the levels of PINK1, Parkin, and LC3-II and decreased SQSTM1/p62 (Figures 5C,D).

**Figure 5:**
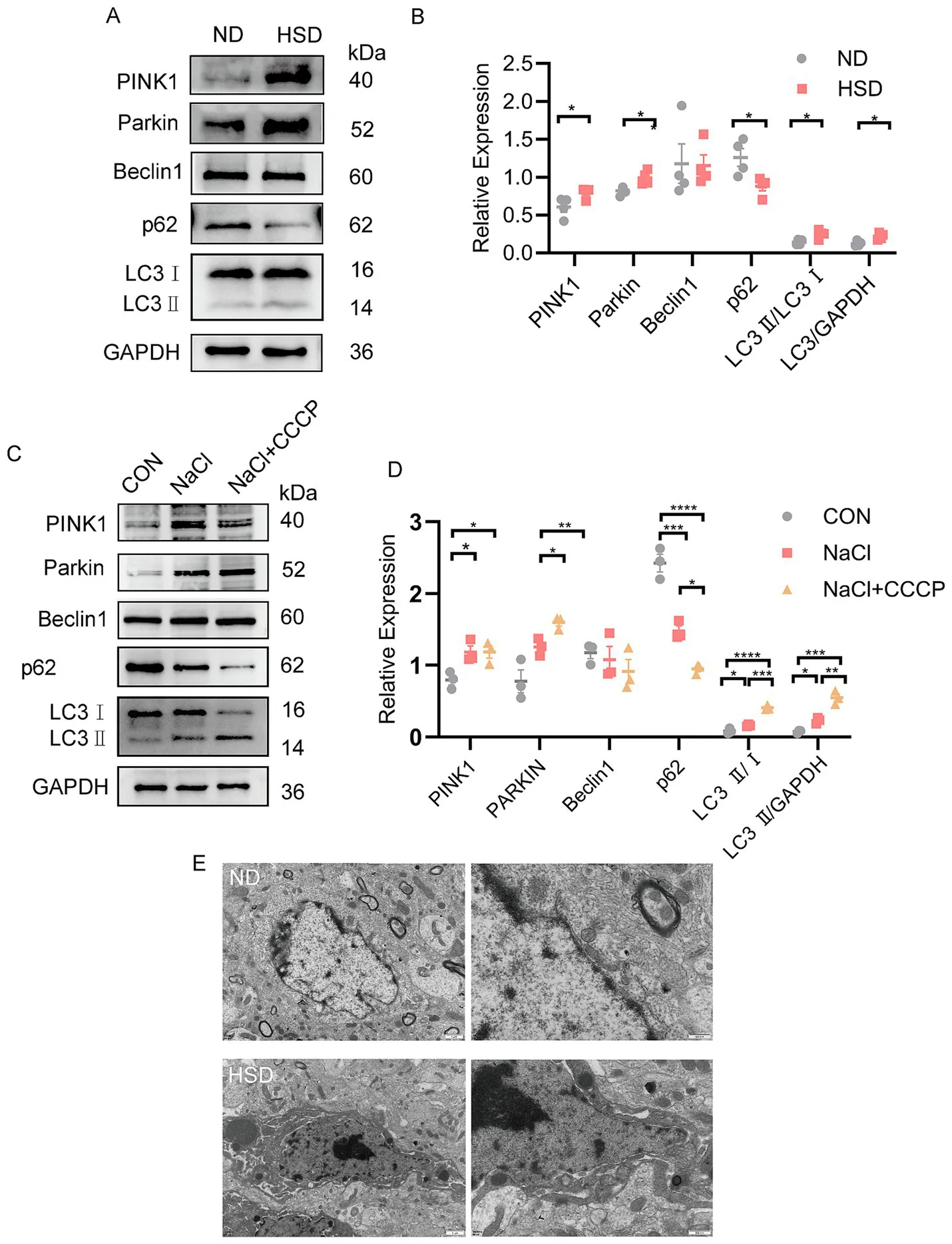
High-salt diet increases mitophagy-associated markers *in vivo* and *in vitro*. **(A,B)** Expression of PINK1, Parkin, and LC3-II (normalized to GAPDH) was elevated, while SQSTM1/p62 was reduced, in HSD-fed mice. **(C,D)** In N2a cells, co-treatment with CCCP (20 μM) further increased PINK1, Parkin, and LC3-II and decreased SQSTM1/p62. **(E)** Neuronal ultrastructure in ND and HSD groups visualized by transmission electron microscopy. CON, control; HSD, high-salt diet; ND, normal diet. Scale bars, 1 μm (×12,000) and 500 nm (×30,000).

**Figure 6:**
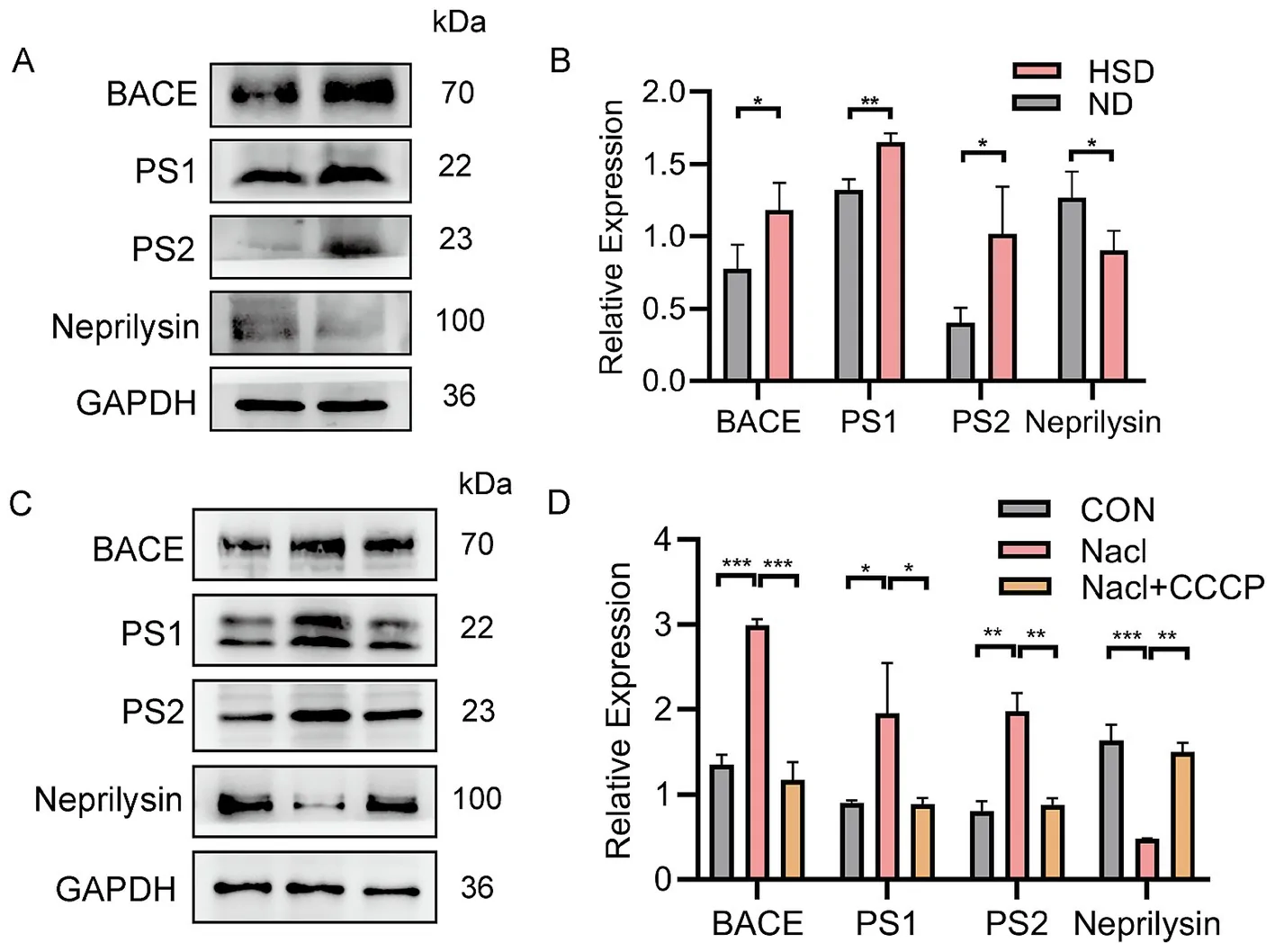
High-salt intake altered Aβ-associated proteins expression. **(A,B)** The protein expression of BACE, PS1, PS2 were increased and the protein of neprilysin was reduced in HSD group. **(C,D)** In N2a cells, co-treatment with CCCP significantly decreased the levels of BACE, PS1, and PS2 and enhanced the level of neprilysin. CON, control; HSD, high-salt diet.

**Figure 7:**
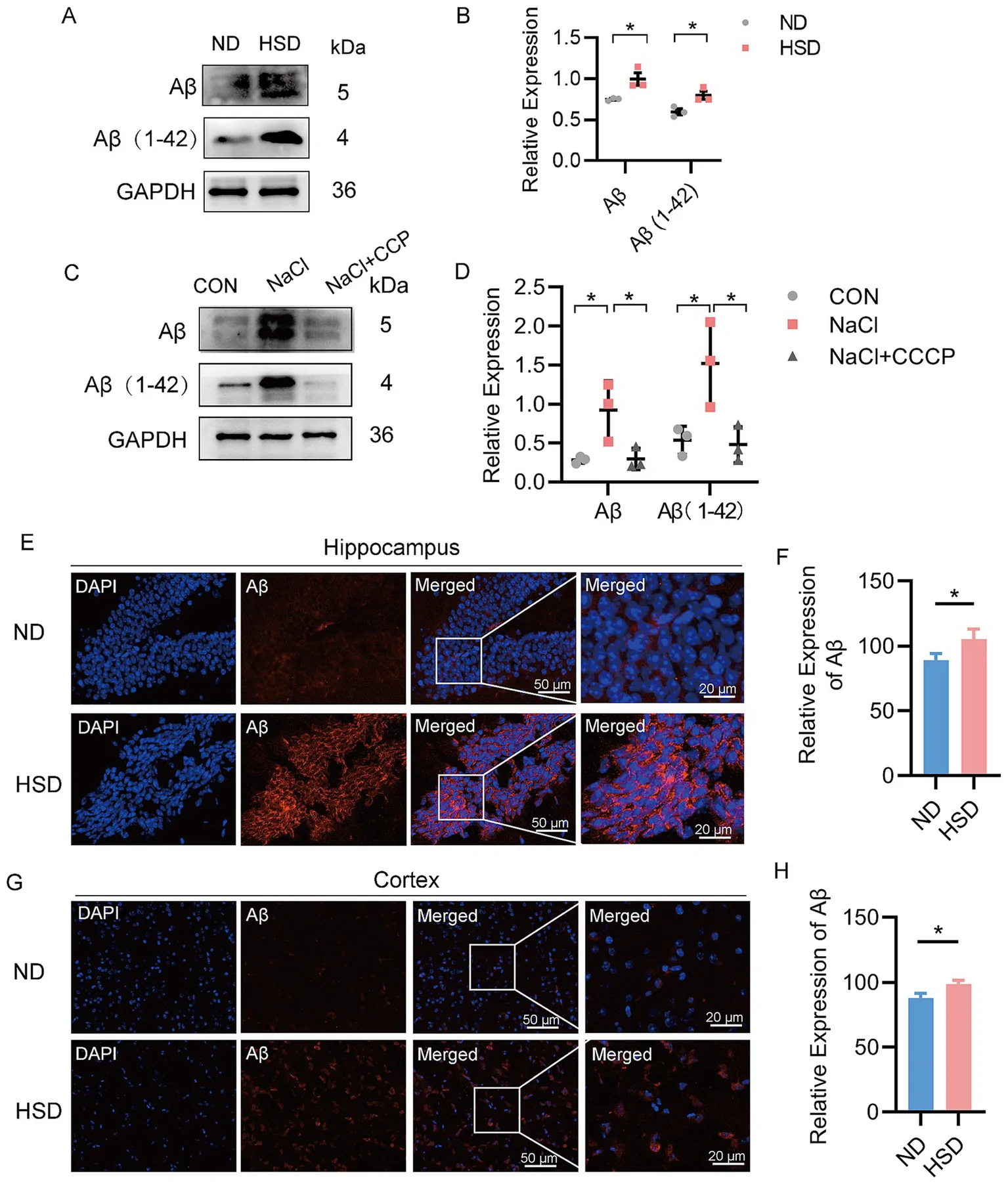
Enhanced mitophagy attenuates Aβ accumulation induced by an HSD. **(A,B)** Western blot analysis showed that the protein levels of Aβ and Aβ (1–42) were significantly elevated in the HSD group. In contrast, *in vitro* treatment with NaCl combined with CCCP markedly reduced Aβ and Aβ (1–42) expression **(C,D)**. Immunoffuorescence further conffrmed the distribution of Aβ in the hippocampus and cortex (E-H) (*n* = 3/group). Scale bars: 10 μm and 5 μm. CON, control; HSD, high-salt diet.

We used transmission electron microscopy to observe the morphology of neurons. The nuclei of neurons are irregular polygons with uniform distribution of chromatin, mainly euchromatin, and clear and complete nuclear membrane in normal diet mice (E). Mitochondria with complete and clear structure can be seen in the cytoplasm. However, in HSD mice, some neuronal cells suffered necrosis and apoptosis (nuclear shrinkage, cell membrane discontinuity), most mitochondria were swollen in the cytoplasm, and a small number of mitochondrial autophagy and lysosomes were also seen (Figure 5E).

### 3.3 High salt diet induces an increase in aβ

To explore whether mitophagy improved cognitive ability and Aβ, we firstly measured proteins level in Aβ-related. As shown in Figures 6A,B, HSD significantly increased the protein expression of BACE, PS1, PS2 and reduced neprilysin protein level. *In vitro*, co-treatment with CCCP significantly decreased the levels of BACE, PS1, and PS2 and restored the level of neprilysin (Figures 6C,D).

As shown in Figures 7A,B, HSD significantly increased the protein expression of Aβ and Aβ (1–42). *in vitro* experiment, CCCP significantly decreased the levels of Aβ and Aβ (1–42) (Figures 7C,D). That suggests that enhanced mitophagy can inhibit the formation of Aβ high fat diet. In addition, immunofluorescence was performed to analyse the expression of Aβ (Figures 7E–H), which is consistent with the results obtained by Western blotting.

## 4 Discussion

We studied the mechanism of the adverse impact of salt on the brain. It has been demonstrated that HSD causes alterations in endothelial function in the brain and systemic vasculature due to a decrease in endothelial NO. However, the majority of research has focused on salt-induced hypertension, and an HSD can lead to neurovascular changes that ultimately lead to cognitive impairments affecting different cognitive domains (15). Working with an HSD model that simulates sustained high salt intake in mice, we discovered that an HSD leads to cognitive decline and increased Aβ accumulation in mice, with subsequent changes in the levels of Aβ and its associated proteins upon activation of mitophagy.

Most studies have focused on the effects of HSD on mitophagy. Chronic mitochondrial stress associated with AD can trigger Parkin-dependent mitophagy *in vitro* and *in vivo* (16); increased mitophagy in AD is thus considered a beneficial, compensatory response that degrades dysfunctional mitochondria and protects neurons from oxidative damage. However, this compensatory response cannot fully resist HSD-induced mitochondrial damage, and our data indicate that although mitophagy-associated markers are increased in HSD-fed mice, cognition is significantly impaired and Aβ production is increased. Meanwhile, enhancing mitophagy with CCCP—the most widely used pharmacological inducer of PINK1/Parkin-mediated mitophagy (3, 10)—reduced Aβ production *in vitro*, suggesting that pharmacological enhancement of mitophagy may attenuate the effects of HSD; however, CCCP is not pathway-specific (11), and this observation requires validation with pathway-specific approaches and *in vivo* models. In addition, because CCCP alters mitochondrial respiration and protein homeostasis more broadly, the present *in vitro* findings should be interpreted as showing a concordance between enhanced PINK1/Parkin-associated markers and reduced Aβ rather than a direct mitophagy–Aβ causal chain. These findings are consistent with recent studies reporting increased mitophagy together with pathology in HSD-fed mice (17). Emerging evidence suggests that excessive salt intake may compromise spatial memory retention, possibly via elevated oxidative stress in the hippocampus, a vital structure implicated in neurodegenerative disease progression (18). In the behavioral test, the C57BL/6 that were given HSD did not display any signs of anxiety, but they did exhibit cognitive impairment. This effect was related to the loss of the striatum (19). There are similarities to our research in C57BL/6J mice. Even more studies have reported that the offspring of subjects with maternal HSD exhibit short- and long-term memory deficits in adulthood, especially in spatial memory. Impairment of hippocampal synaptic transmission and plasticity was observed in adult offspring (1).

APP is cleaved by β- and γ-secretase, releasing neurotoxic Aβ peptides that can aggregate into oligomeric aggregates. In the amyloidosis pathway, the transmembrane protein APP is cleaved by α-secretase, β-secretase (BACE1), and γ-secretase, which consists mainly of four subunits, presenilin (PS1, PS2), nicastrin, APH-1, and PEN-2, and mutations in the presenilin-1 (PSEN1) and presenilin-2 (PSEN2) genes increase the cleavage of γ-secretase at this site. They promote the production of Aβ and the formation of amyloid plaques (Figure 8). However, Aβ-degrading enzyme neprilysin can significantly reduce Aβ pathology (20). The current hypothesis about Aβ suggests that insoluble low-molecular-weight substance can disrupt the communication between neurons. Additionally, this substance may aggregate into insoluble β-sheet-rich amyloid fibrils, potentially leading to localized inflammatory responses. Over time, this may eventually result in the death of neurons and the development of characteristic neuronal lesions associated with AD (21). This is consistent with our findings that HSD mice had increased neuronal cell necrosis compared to control animals. Hippocampal neuronal apoptosis is a major mechanism of cognitive and synaptic plasticity impairment. During the pathway of cell apoptosis, activated caspases interfere with the electron transport chain complexes I and II, leading to mitochondrial dysfunction and the production of ROS (22). Neuroinflammation is established to be related to the pathology of neurodegenerative diseases such as cognitive dysfunction (23). Inflammation is considered as the root cause of the disease, or as the immune system’s obstruction to control the accumulation of Aβ in the brain (24).

**Figure 8:**
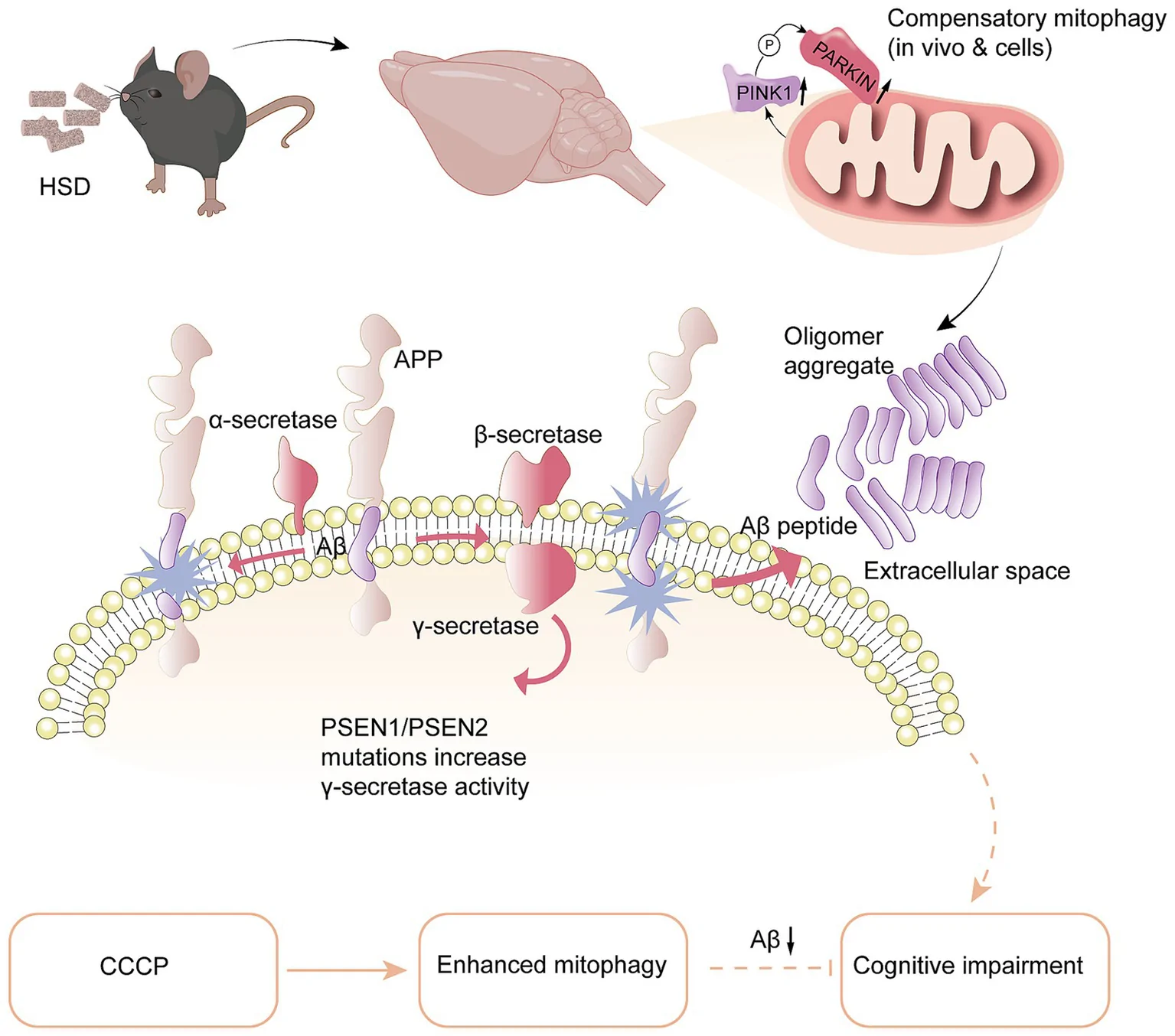
Proposed mechanism of cognitive impairment induced by an HSD. HSD induces mitochondrial damage (mitochondrial depolarization and swelling), which triggers a compensatory but insufficient activation of PINK1/Parkin-mediated mitophagy; the accumulation of damaged mitochondria promotes Aβ production and cognitive impairment. Solid arrows indicate findings supported by the present data; dashed arrows indicate proposed links that require experimental validation. Enhancing mitophagy attenuated Aβ accumulation *in vitro*.

On the basis of our observations, caution in interpreting these findings is essential owing to potential variations and discrepancies. For example, the effects of a high-salt diet may vary widely based on the mouse model used, each of which may have unique metabolic and immune responses. It is likewise true that the type of cells for *in vitro* experiments, whether they are primary cells derived directly from the tissues of high salt-fed mice or cultured cell lines, has profound effects on the outcome of the experiments. In this study, we observe that HSD increases water and food intake. However, 2 months of high-intensity HSD have not significantly increased weight. Previous studies have shown that weight gain is associated with cognitive impairment (24). A possible explanation for this might be that it is related to the duration of HSD feeding.

Several limitations of the present study should be acknowledged. First, blood pressure and systemic metabolic parameters were not measured; because an 8% NaCl diet can affect the cerebral vasculature and systemic physiology, we cannot exclude the possibility that systemic effects contribute to the observed cognitive changes (15), and we refrain from claiming that these effects occur independently of blood pressure. Second, the *in vivo* experiment was performed exclusively in male mice; although this design avoids the confounding influence of the estrous cycle (25), whether the findings can be generalized to female mice requires confirmation. Third, autophagic flux and mitochondrial–lysosomal co-localization were not directly assessed; because static mitophagy markers cannot distinguish enhanced mitochondrial clearance from impaired lysosomal degradation, direct flux measurements (for example, mito-QC or mt-Keima reporters, or lysosomal blockade) will be required to confirm the present interpretation (14); baseline cognitive performance was not assessed before dietary intervention; although random allocation should minimize pre-existing differences, we cannot exclude baseline variation between groups. Fourth, the *in vitro* model involves acute exposure of N2a cells to additional NaCl, which differs substantially from chronic dietary exposure in aged mice with respect to duration, physiological context, cell type, and systemic influences; extrapolation of the cell-culture findings to the *in vivo* phenotype should therefore be made with caution. Fifth, the mitophagy-enhancing intervention was performed only *in vitro*; whether pharmacological enhancement of mitophagy improves cognition in HSD-fed mice remains to be tested *in vivo*. Sixth, a causal requirement for the PINK1/Parkin pathway was not established experimentally. CCCP was selected as the mitophagy-enhancing intervention because it is the most widely used pharmacological inducer of PINK1/Parkin-mediated mitophagy (3, 10); however, as a protonophore that acutely dissipates the mitochondrial membrane potential, CCCP is not pathway-specific and may influence Aβ metabolism through mechanisms independent of mitophagy (11). In the absence of pathway-specific interventions, such as PINK1 or Parkin knockdown or knockout, the present findings support—but do not prove—a causal contribution of PINK1/Parkin-mediated mitophagy to HSD-induced Aβ accumulation; establishing this causal link will require genetic loss-of-function approaches *in vitro* and *in vivo* (26). Seventh, the present *in vitro* design has specific limitations. Although NaCl alone at 40 mM did not significantly reduce N2a cell viability (Figure 1B), cell viability under the combined NaCl (40 mM) + CCCP (20 μM) condition was not formally assessed in this study. Another, a CCCP-only control group was not included; therefore, the possibility that CCCP alters Aβ metabolism through mechanisms independent of mitophagy (e.g., direct effects on mitochondrial respiration, oxidative stress, or protein turnover) cannot be excluded (11). We have therefore been cautious in attributing the observed reductions of BACE, PS1/PS2, neprilysin, Aβ, and Aβ (1–42) solely to enhanced mitophagy, and we frame these *in vitro* observations as consistent with a mitophagy-dependent contribution that remains to be confirmed by pathway-specific interventions and additional controls. Finally, the duration and composition of the diet can markedly influence outcomes, and short-term feeding may not reproduce the effects of long-term exposure; future studies with larger sample sizes and varied dietary protocols are needed to confirm and extend the present findings.

Recently, dietary therapy has appeared as a promising adjunctive therapeutic strategy for neurodegenerative diseases, autoimmune disorders, cardiovascular diseases, and metabolic disorders. For example, the Mediterranean diet may reduce AD risk and prevent memory loss and mesial temporal lobe atrophy by decreasing amyloid-β and phosphorylated tau protein levels. These interventions may show substantial potential in regulating metabolic and therapeutic responses (27).

In conclusion, HSD feeding induces mitochondrial damage, activates a compensatory but insufficient PINK1/Parkin-mediated mitophagy response, promotes Aβ accumulation, and impairs cognition in aged mice; pharmacological enhancement of mitophagy attenuated the NaCl-induced Aβ elevation *in vitro*, although a causal role for PINK1/Parkin-mediated mitophagy remains to be established. Strategies aimed at restoring PINK1/Parkin-mediated mitophagy may thus hold therapeutic promise for counteracting the harmful effects of HSD on the brain. Currently, dietary interventions hold great promise as a new approach to disease management, as dietary changes often produce fewer side effects than drug treatments and interventions are easier to implement. With further research, dietary interventions could become an integral part of personalized medicine, offering new ways to prevent and treat countless diseases.

## Data Availability

The original contributions presented in the study are included in the article/Supplementary material, further inquiries can be directed to the corresponding authors.

## References

[1] Ge Q Hu X Ma N Sun M Zhang L Cai Z . Maternal high-salt diet during pregnancy impairs synaptic plasticity and memory in offspring. FASEB J. (2021) 35:e21244. doi: 10.1096/fj.202001890R, 33715195

[2] Eldeeb MA Fallahi A Soumbasis A Bayne AN Trempe J-F Fon EA. Mitochondrial import stress and PINK1-mediated mitophagy: the role of the PINK1-TOMM-TIMM23 supercomplex. Autophagy. (2024) 20:1903–5. doi: 10.1080/15548627.2024.2340399, 38597070 PMC11262206

[3] Lazarou M Sliter DA Kane LA Sarraf SA Wang C Burman JL . The ubiquitin kinase PINK1 recruits autophagy receptors to induce mitophagy. Nature. (2015) 524:309–14. doi: 10.1038/nature14893, 26266977 PMC5018156

[4] Du F Yu Q Yan S Hu G Lue LF Walker DG . PINK1 signalling rescues amyloid pathology and mitochondrial dysfunction in Alzheimer's disease. Brain. (2017) 140:3233–51. doi: 10.1093/brain/awx258, 29077793 PMC5841141

[5] Gwon AR Park JS Arumugam TV Kwon YK Chan SL Kim SH . Oxidative lipid modification of nicastrin enhances amyloidogenic γ-secretase activity in Alzheimer's disease. Aging Cell. (2012) 11:559–68. doi: 10.1111/j.1474-9726.2012.00817.x, 22404891 PMC4217088

[6] Du H Guo L Yan S Sosunov AA McKhann GM Yan SS. Early deficits in synaptic mitochondria in an Alzheimer's disease mouse model. Proc Natl Acad Sci USA. (2010) 107:18670–5. doi: 10.1073/pnas.1006586107, 20937894 PMC2972922

[7] Chen HC Cao JX Zhang YS Ma YZ Zhang L Su XM . High salt diet exacerbates cognitive deficits and neurovascular abnormalities in APP/PS1 mice and induces AD-like changes in wild-type mice. J Nutr Biochem. (2024) 125:109570. doi: 10.1016/j.jnutbio.2024.109570, 38218348

[8] Geisberger S Bartolomaeus H Neubert P Willebrand R Zasada C Bartolomaeus T . Salt transiently inhibits mitochondrial energetics in mononuclear phagocytes. Circulation. (2021) 144:144–58. doi: 10.1161/CIRCULATIONAHA.120.052788, 33906377 PMC8270232

[9] Forte M Bianchi F Cotugno M Marchitti S Stanzione R Maglione V . An interplay between UCP2 and ROS protects cells from high-salt-induced injury through autophagy stimulation. Cell Death Dis. (2021) 12:919. doi: 10.1038/s41419-021-04188-4, 34625529 PMC8501098

[10] Narendra D Tanaka A Suen DF Youle RJ. Parkin is recruited selectively to impaired mitochondria and promotes their autophagy. J Cell Biol. (2008) 183:795–803. doi: 10.1083/jcb.200809125, 19029340 PMC2592826

[11] Georgakopoulos ND Wells G Campanella M. The pharmacological regulation of cellular mitophagy. Nat Chem Biol. (2017) 13:136–46. doi: 10.1038/nchembio.2287, 28103219

[12] Cai Z Pan T Pu Y Zou Q Jing F Wen J . Dietary salt disrupts tricarboxylic acid cycle and induces tau hyperphosphorylation and synapse dysfunction during aging. Aging Dis. (2022) 13:1532. doi: 10.14336/ad.2022.0220, 36186135 PMC9466974

[13] Mizushima N Yoshimori T. How to interpret LC3 immunoblotting. Autophagy. (2007) 3:542–5. doi: 10.4161/auto.4600, 17611390

[14] McWilliams TG Prescott AR Allen GF Tamjar J Munson MJ Thomson C . Mito-QC illuminates mitophagy and mitochondrial architecture in vivo. J Cell Biol. (2016) 214:333–45. doi: 10.1083/jcb.201603039, 27458135 PMC4970326

[15] Faraco G Brea D Garcia-Bonilla L Wang G Racchumi G Chang H . Dietary salt promotes neurovascular and cognitive dysfunction through a gut-initiated TH17 response. Nat Neurosci. (2018) 21:240–9. doi: 10.1038/s41593-017-0059-z, 29335605 PMC6207376

[16] Wang H Jiang T Li W Gao N Zhang T. Resveratrol attenuates oxidative damage through activating mitophagy in an in vitro model of Alzheimer’s disease. Toxicol Lett. (2018) 282:100–8. doi: 10.1016/j.toxlet.2017.10.021, 29097221

[17] Li Y Zhao K Hu Y Yang F Li P Liu Y. MicroRNA-142-3p alleviated high salt-induced cardiac fibrosis via downregulating optineurin-mediated mitophagy. iScience. (2024) 27:109764. doi: 10.1016/j.isci.2024.109764, 38726368 PMC11079474

[18] Liu YZ Chen JK Li ZP Zhao T Ni M Li DJ . High-salt diet enhances hippocampal oxidative stress and cognitive impairment in mice. Neurobiol Learn Mem. (2014) 114:10–5. doi: 10.1016/j.nlm.2014.04.010, 24752150

[19] Guo CP Wei Z Huang F Qin M Li X Wang YM . High salt induced hypertension leads to cognitive defect. Oncotarget. (2017) 8:95780–90. doi: 10.18632/oncotarget.21326, 29221166 PMC5707060

[20] Wang YY Yan Q Huang ZT Zou Q Li J Yuan MH . Ameliorating Ribosylation-induced amyloid-β pathology by Berberine via inhibiting mTOR/p70S6K signaling. J Alzheimer's Dis. (2021) 79:833–44. doi: 10.3233/JAD-200995, 33361598

[21] Patterson C Feightner JW Garcia A Hsiung GY MacKnight C Sadovnick AD. Diagnosis and treatment of dementia: 1. Risk assessment and primary prevention of Alzheimer disease. CMAJ. (2008) 178:548–56. doi: 10.1503/cmaj.070796, 18299540 PMC2244657

[22] Ricci J-E Gottlieb RA Green DR. Caspase-mediated loss of mitochondrial function and generation of reactive oxygen species during apoptosis. J Cell Biol. (2003) 160:65–75. doi: 10.1083/jcb.200208089, 12515825 PMC2172744

[23] Wyss-Coray T Mucke L. Inflammation in neurodegenerative disease--a double-edged sword. Neuron. (2002) 35:419–32. doi: 10.1016/S0896-6273(02)00794-8, 12165466

[24] Husain I Akhtar M Vohora D Abdin MZ Islamuddin M Akhtar MJ . Rosuvastatin attenuates high-salt and cholesterol diet induced Neuroinflammation and cognitive impairment via preventing nuclear factor KappaB pathway. Neurochem Res. (2017) 42:2404–16. doi: 10.1007/s11064-017-2264-2, 28417263

[25] Beery AK Zucker I. Sex bias in neuroscience and biomedical research. Neurosci Biobehav Rev. (2011) 35:565–72. doi: 10.1016/j.neubiorev.2010.07.002, 20620164 PMC3008499

[26] Pickles S Vigié P Youle RJ. Mitophagy and quality control mechanisms in mitochondrial maintenance. Curr Biol. (2018) 28:R170–85. doi: 10.1016/j.cub.2018.01.004, 29462587 PMC7255410

[27] Xiao Y-L Gong Y Qi Y-J Shao Z-M Jiang Y-Z. Effects of dietary intervention on human diseases: molecular mechanisms and therapeutic potential. Signal Transduct Target Ther. (2024) 9:59. doi: 10.1038/s41392-024-01771-x, 38462638 PMC10925609

