# Supplementary material for: Dietary high salt drives cognitive impairment and Aβ accumulation via an insufficient compensatory mitophagy response
Source: Front Neurol. 2026 Oct 26;17:1922997. doi: 10.3389/fneur.2026.1922997 (PMC13745211; doi:10.3389/fneur.2026.1922997)

PINK1 in brain tissue (ND group，HSD group)

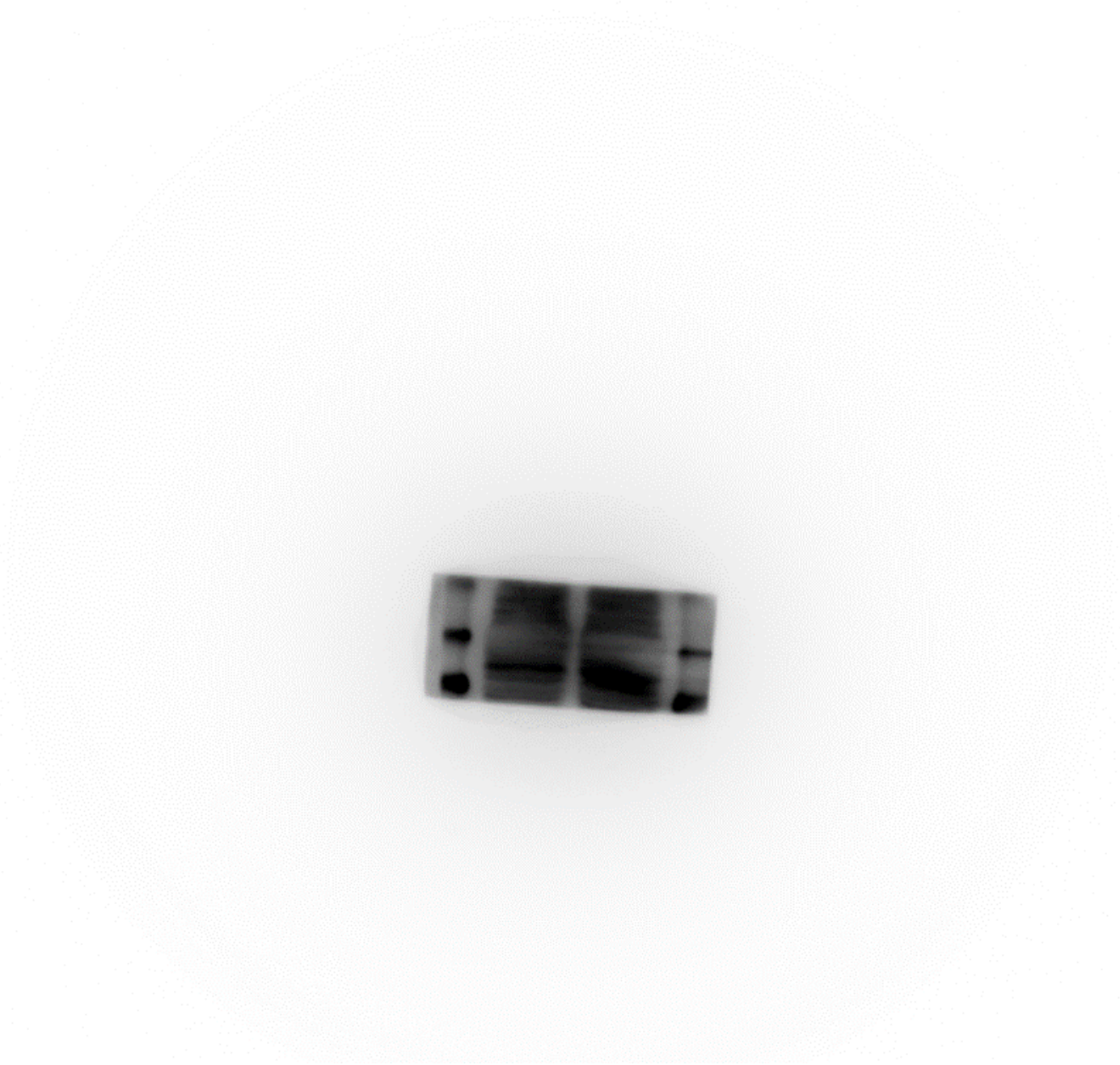

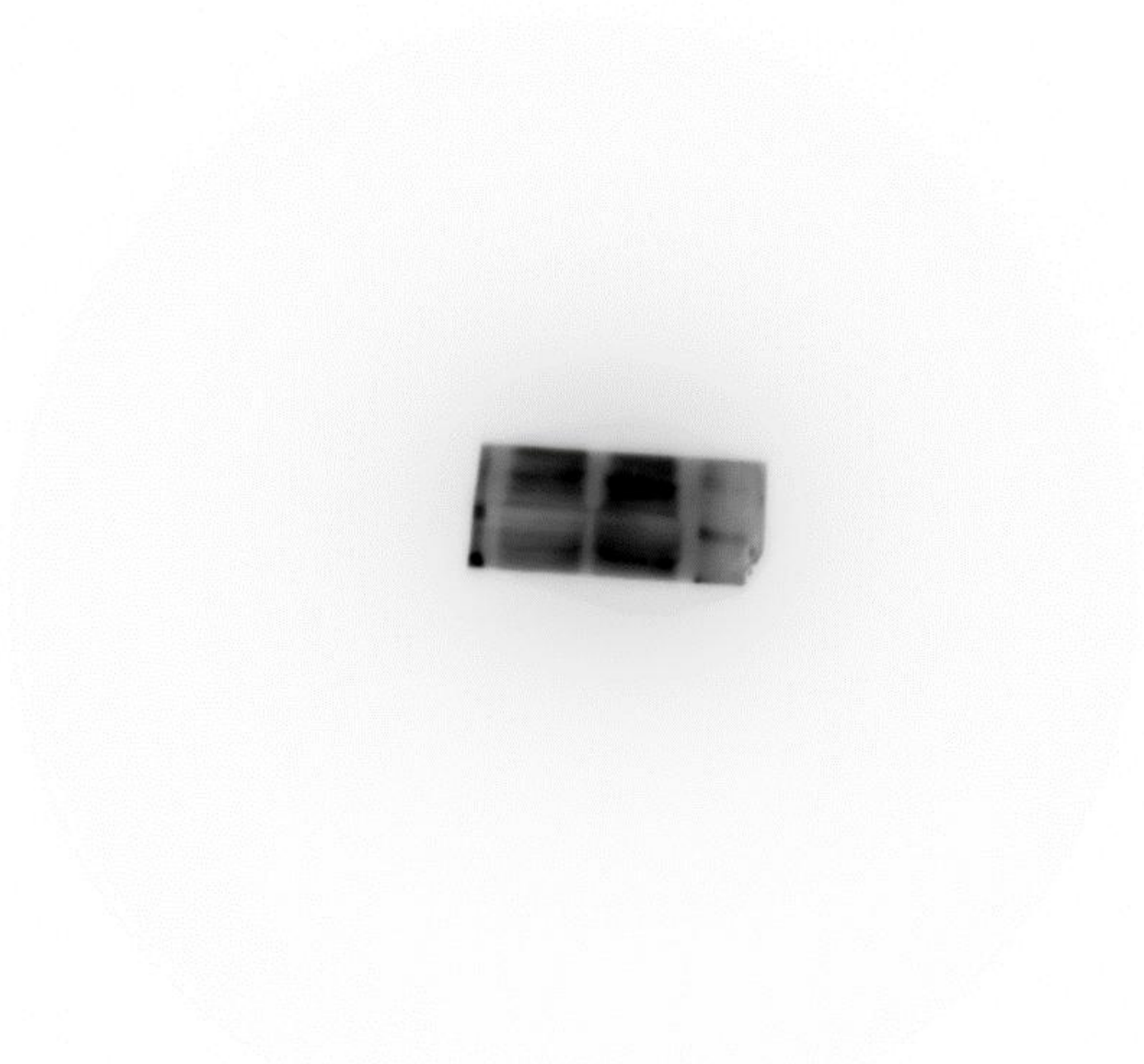

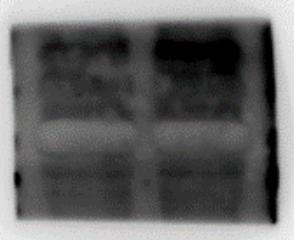

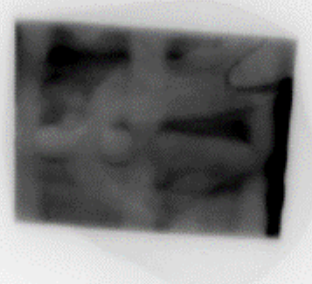

PARKIN in brain tissue (ND group，HSD group)

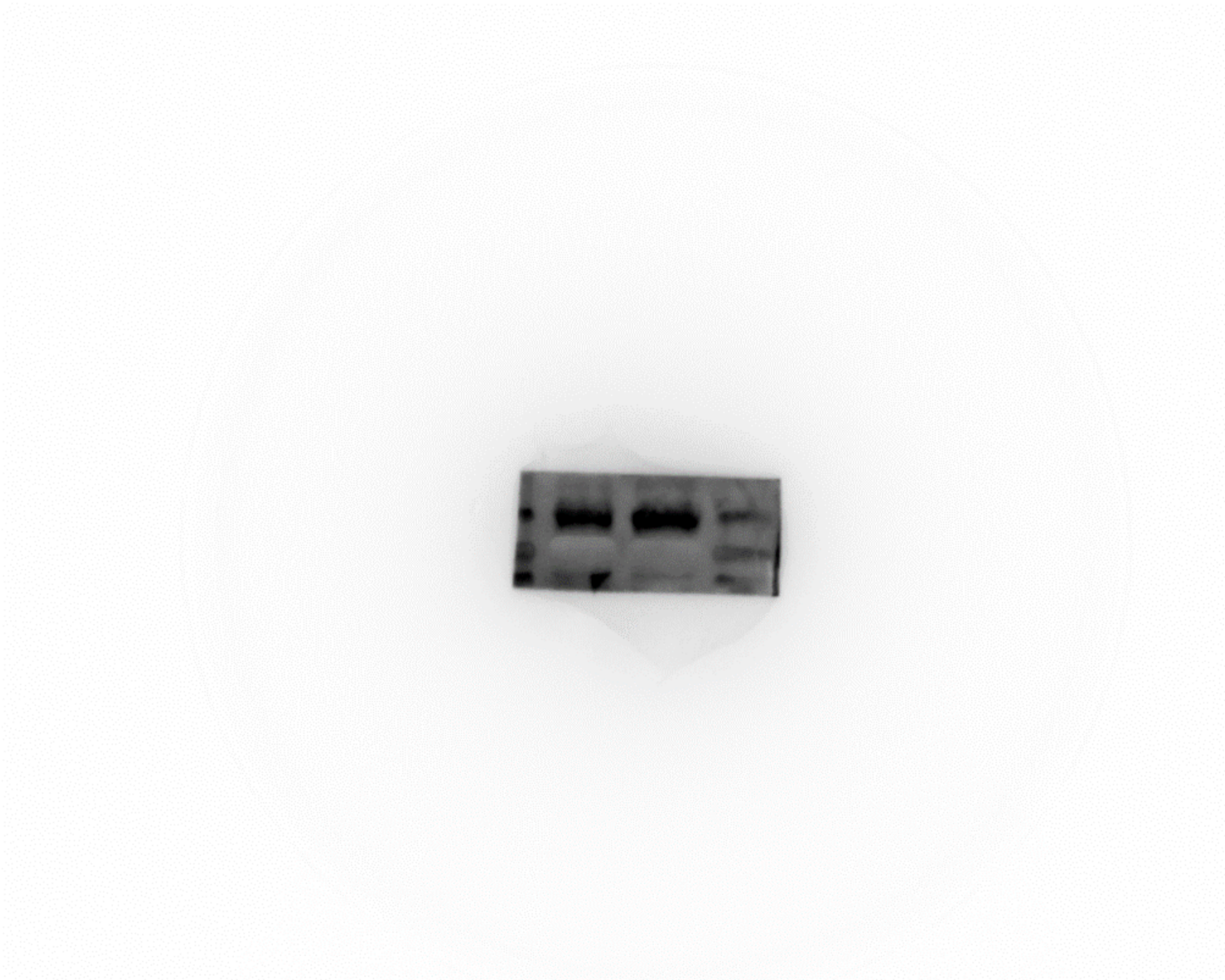

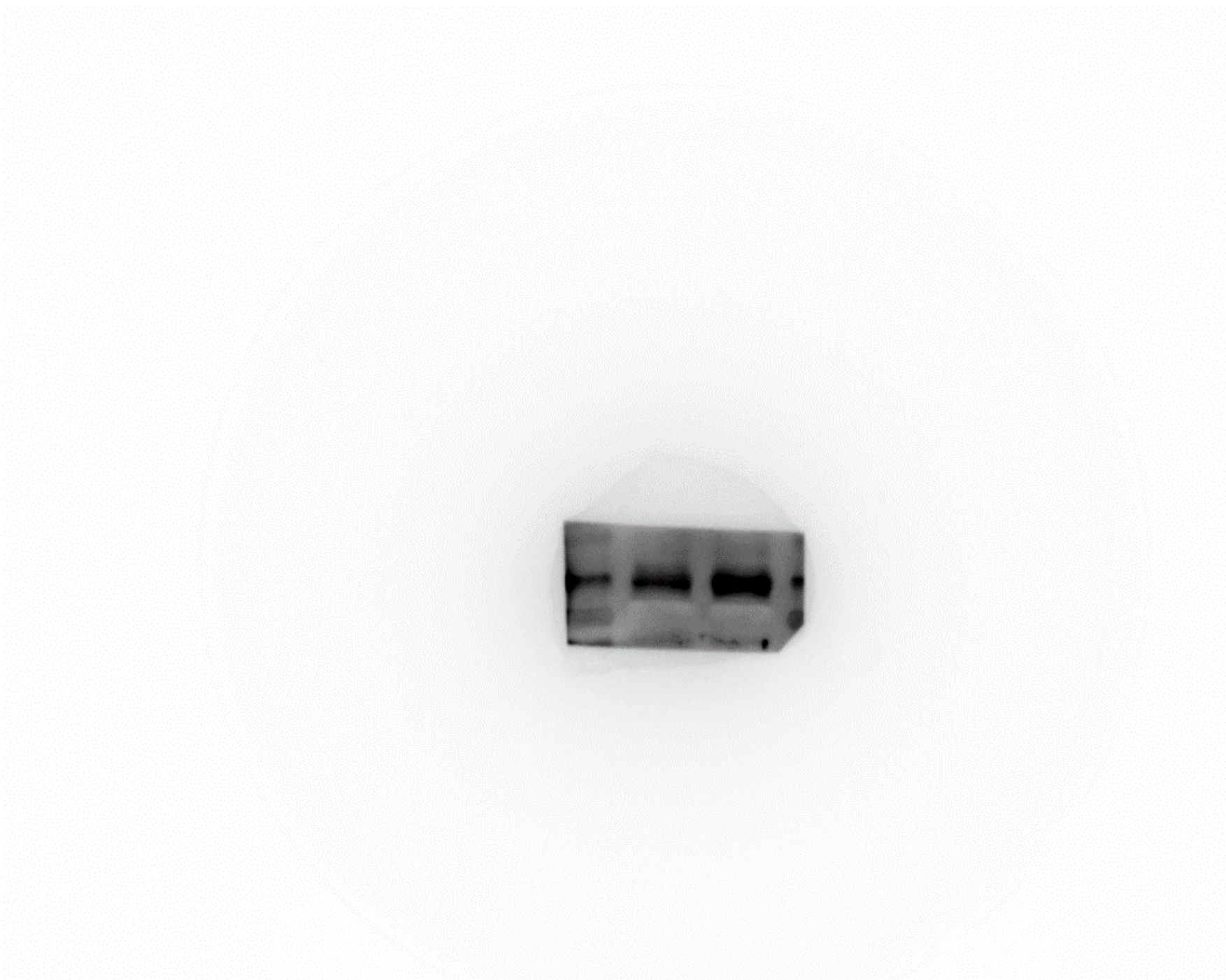

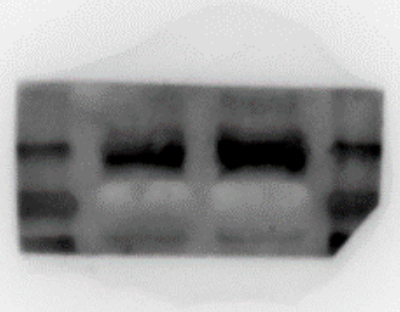

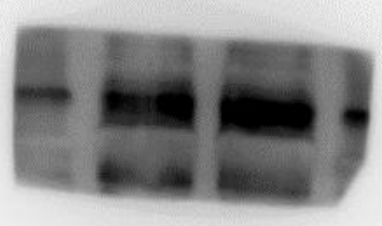

Beclin in brain tissue (ND group，HSD group)

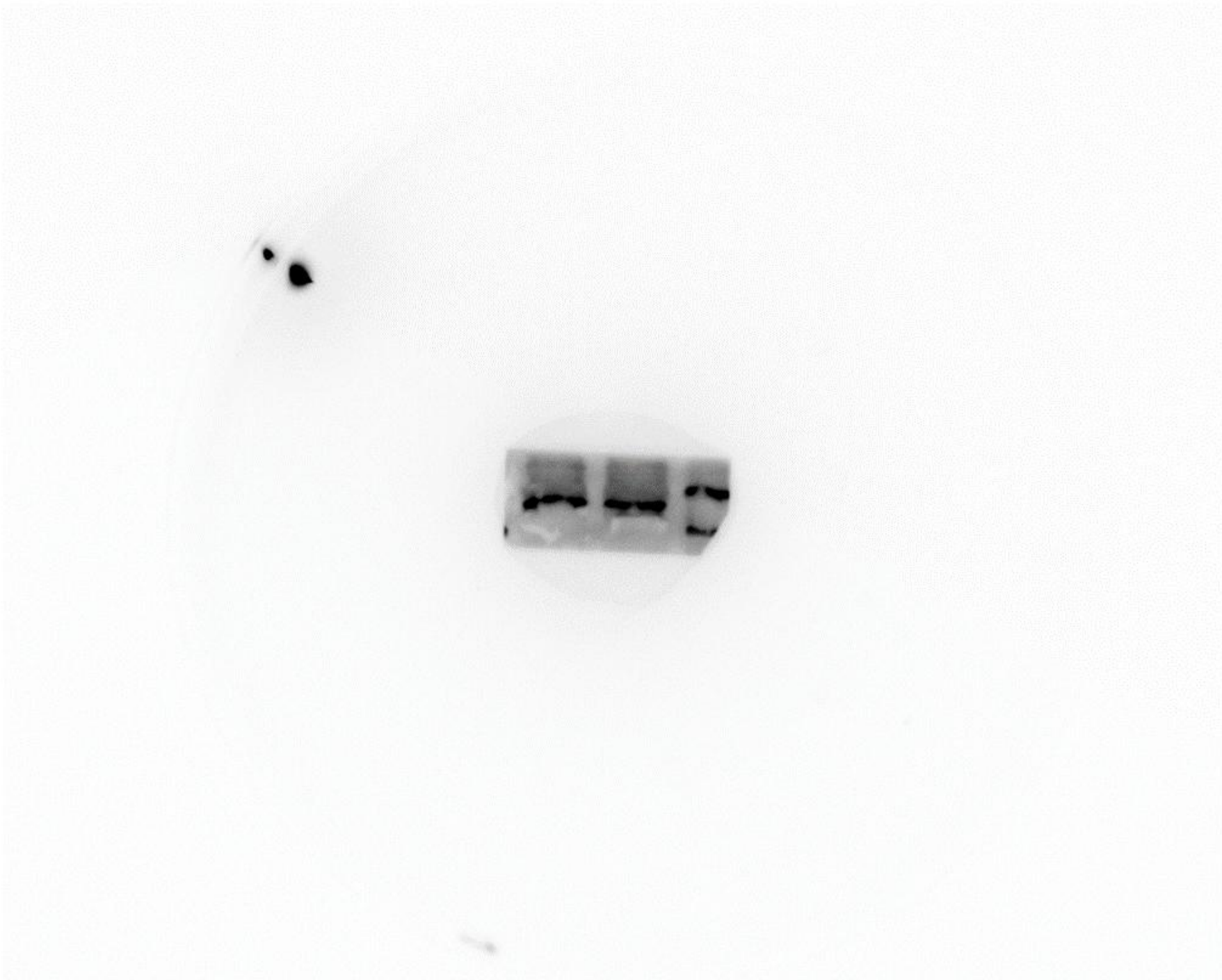

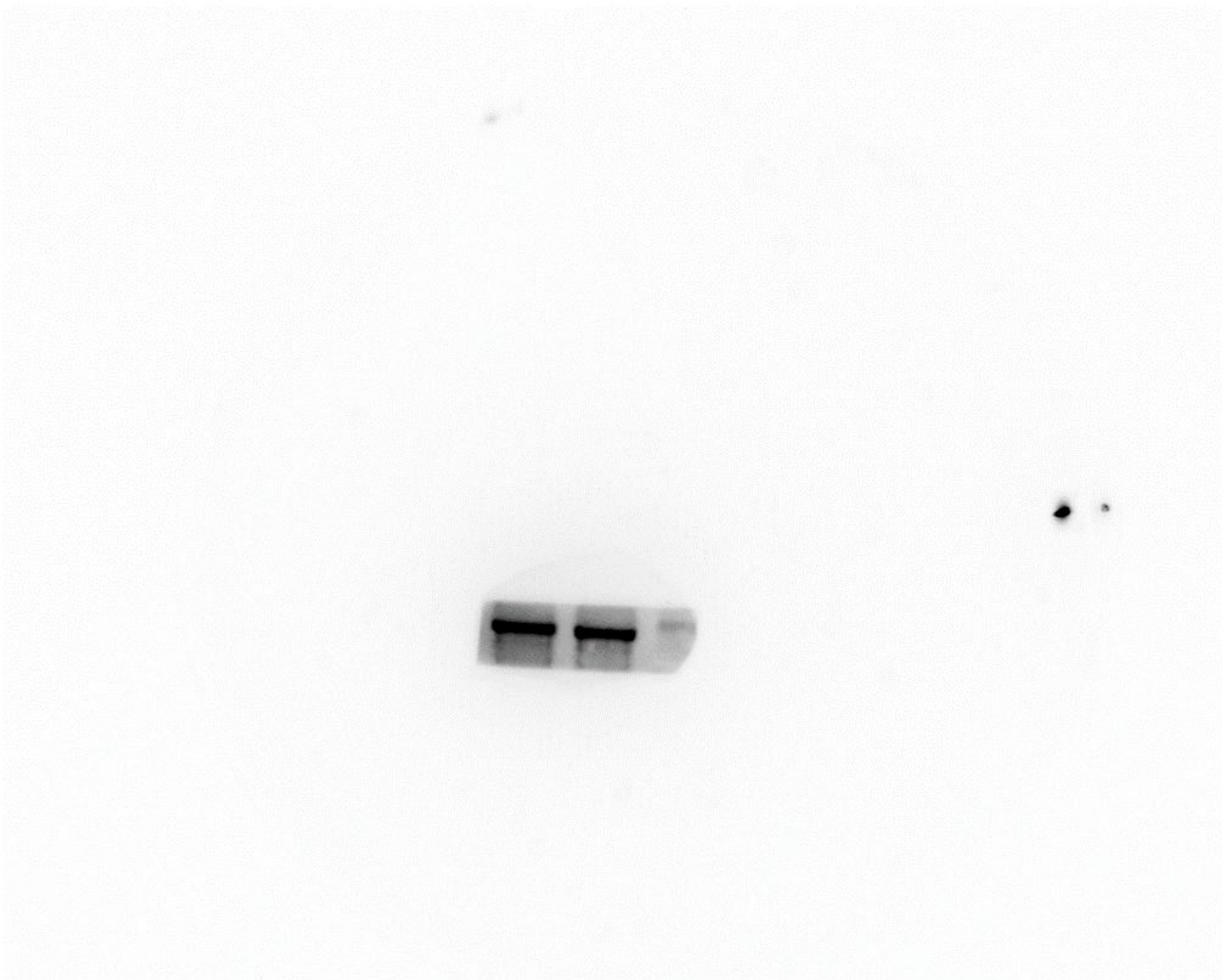

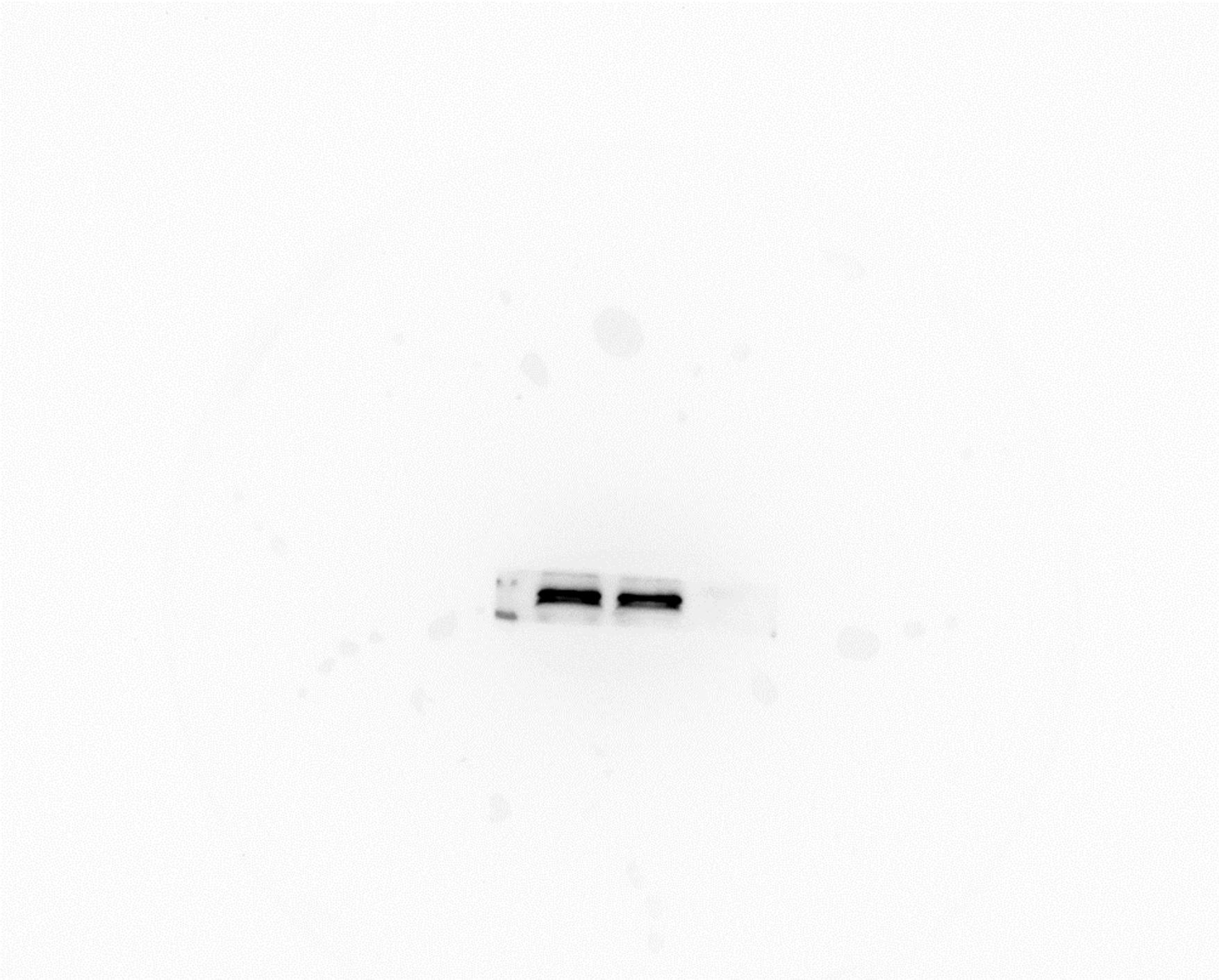

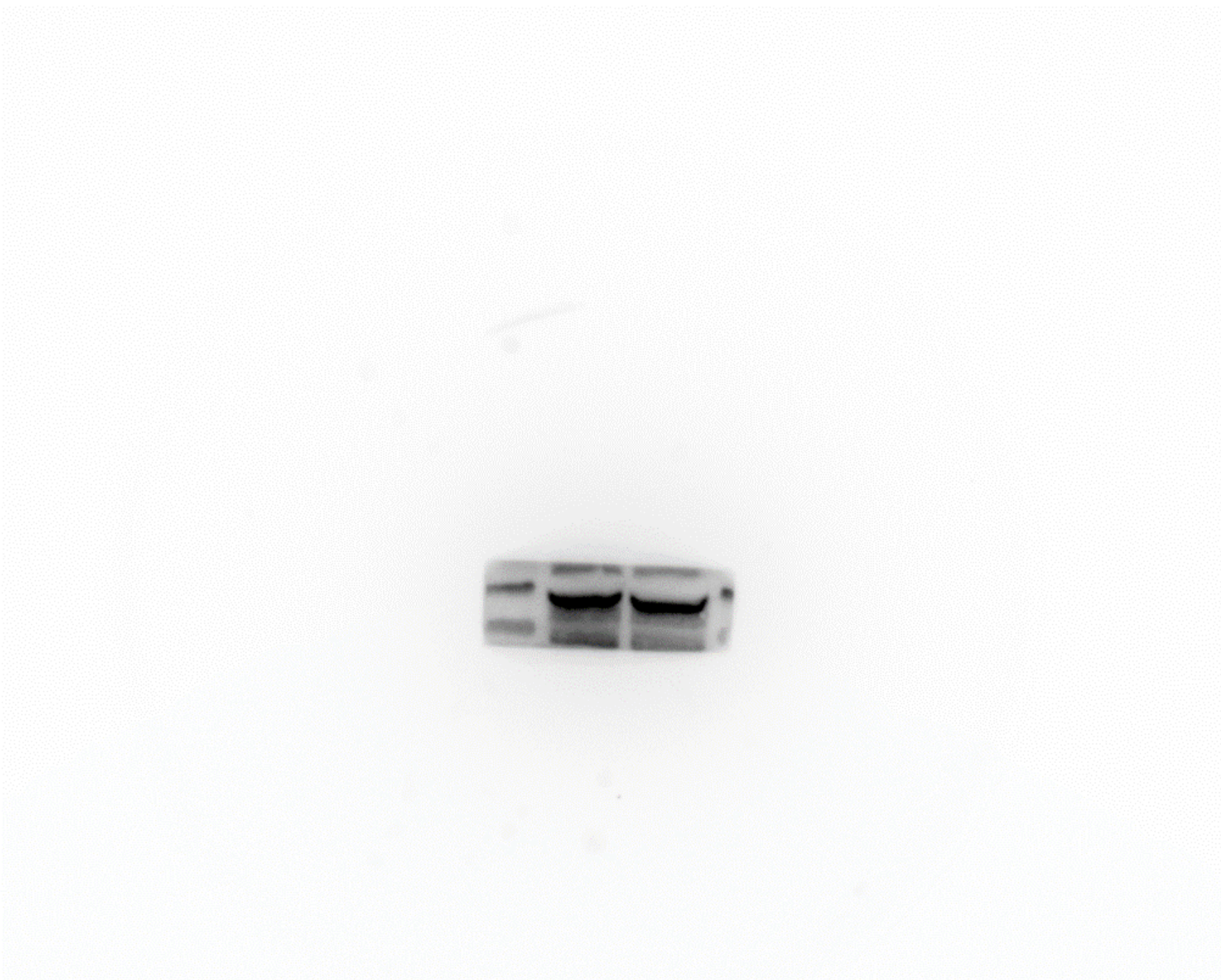

p62 in brain tissue (ND group, HSD group)

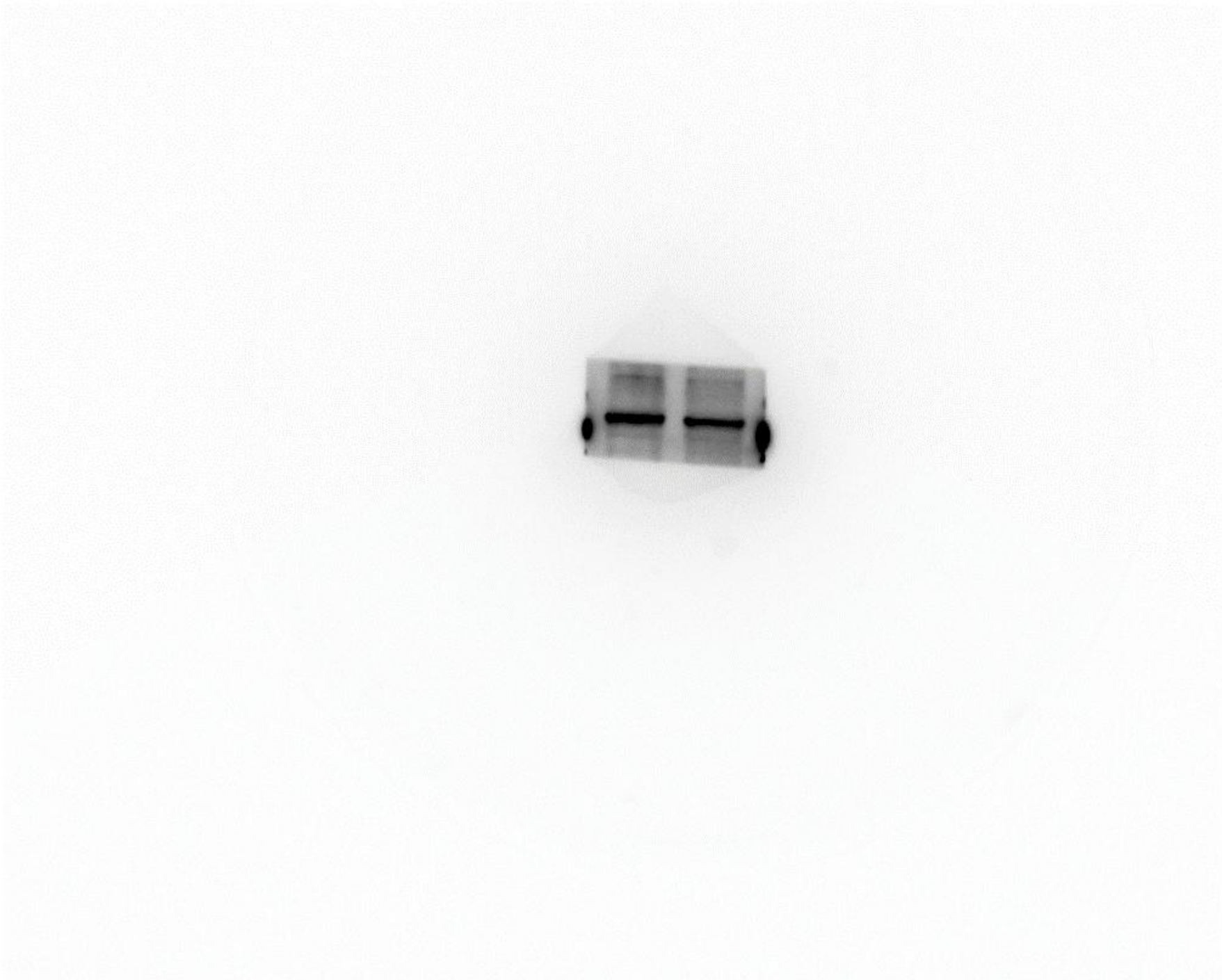

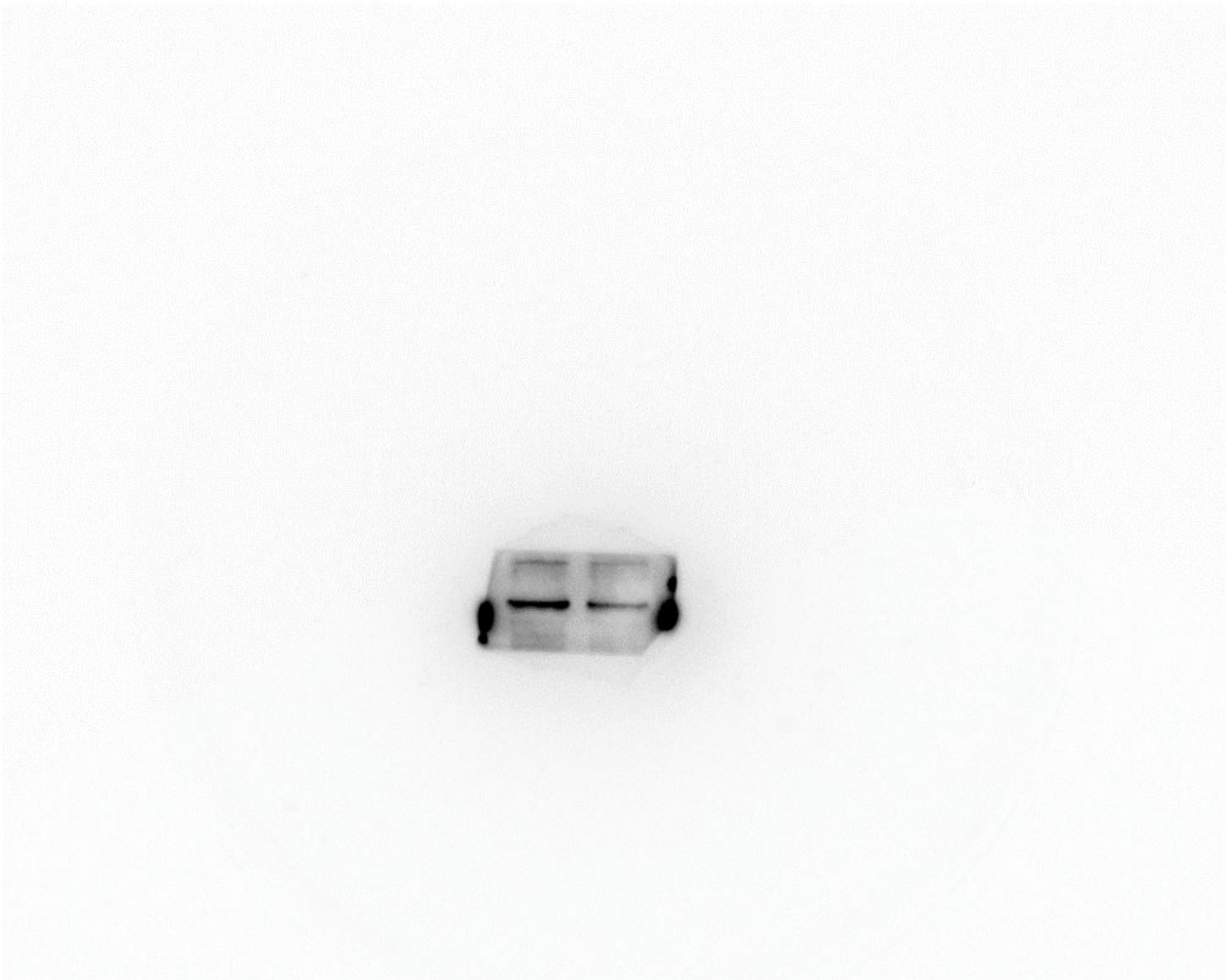

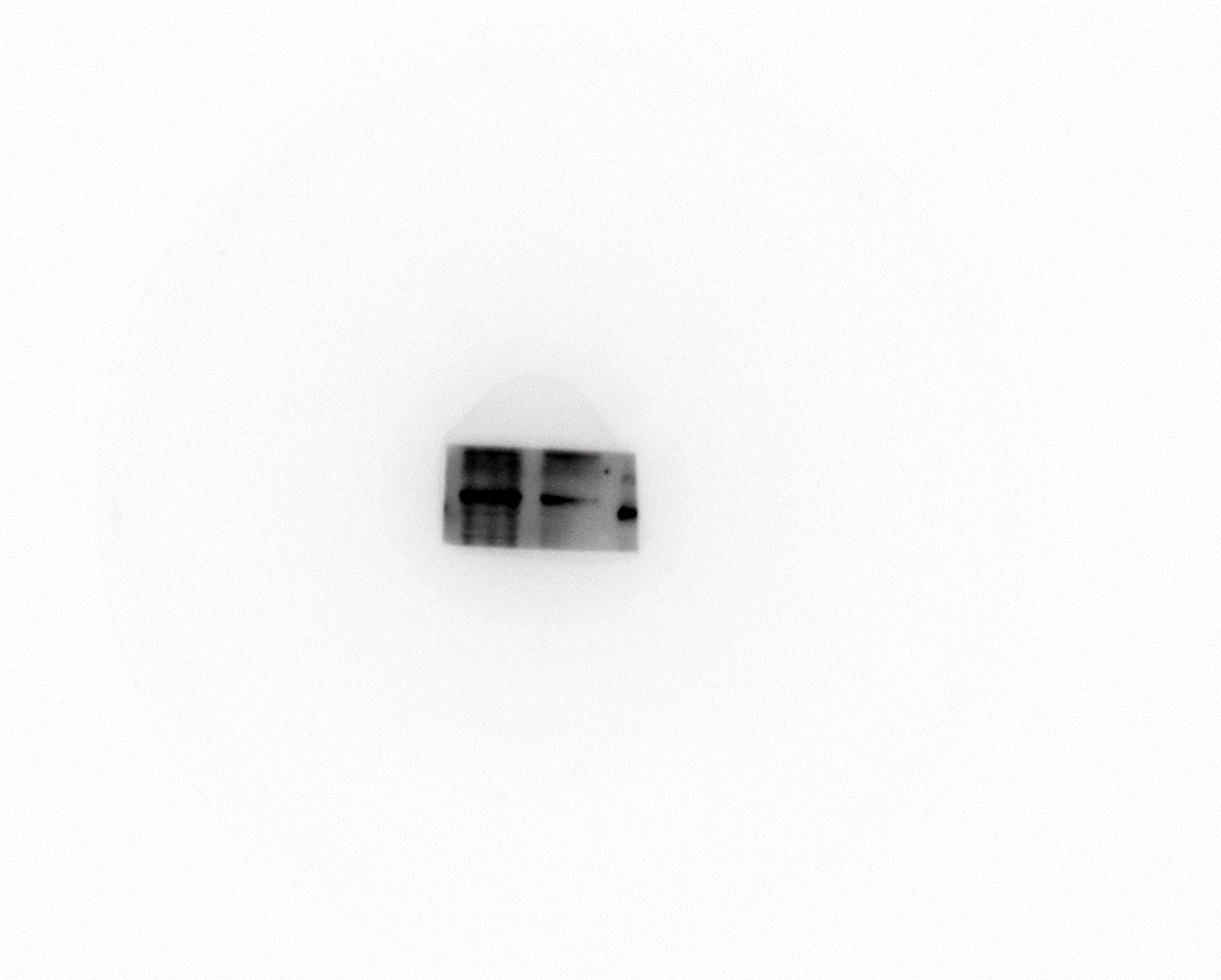

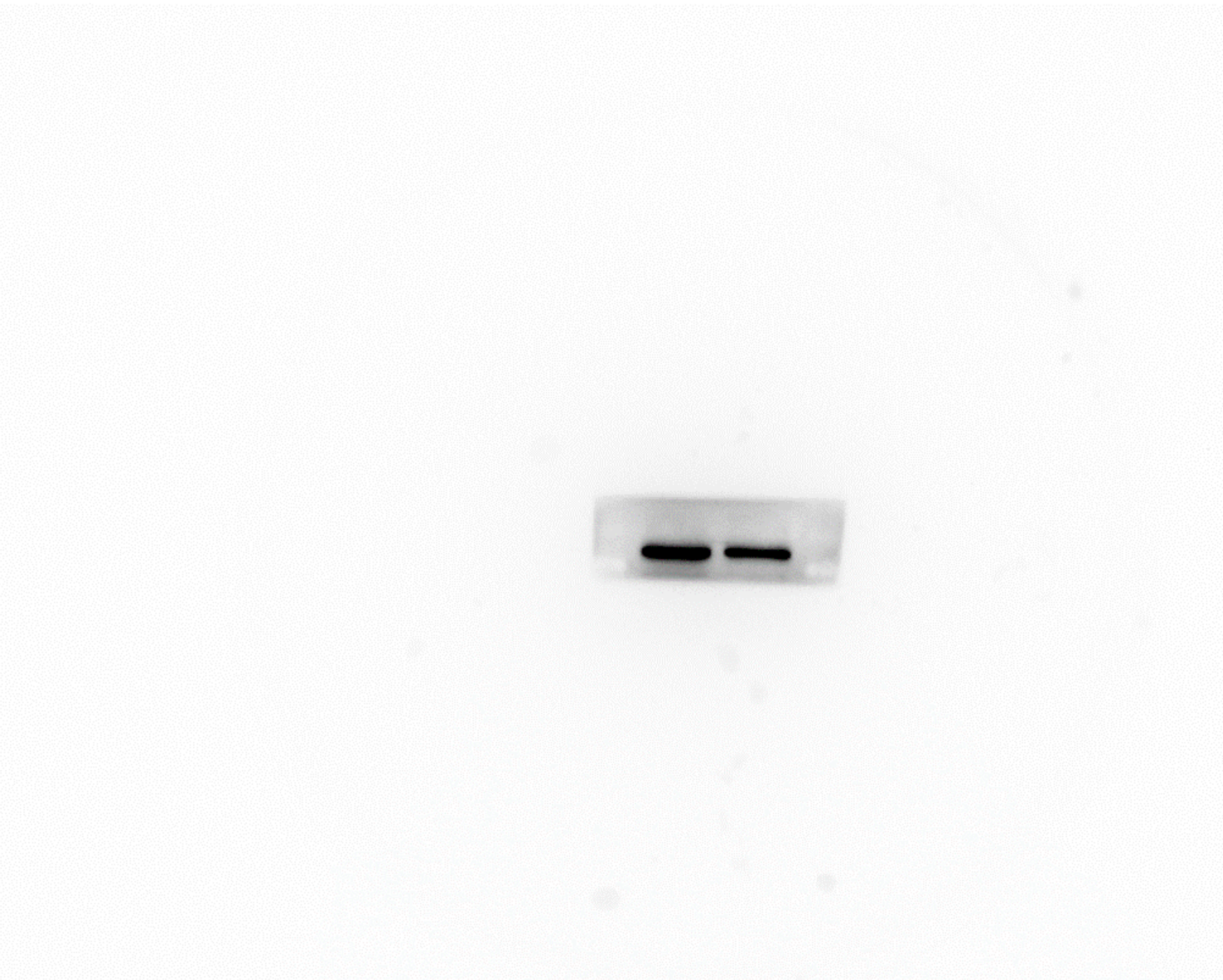

LC3 in brain tissue (ND group，HSD group)

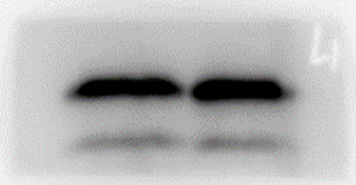

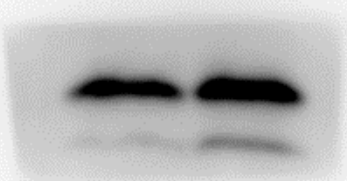

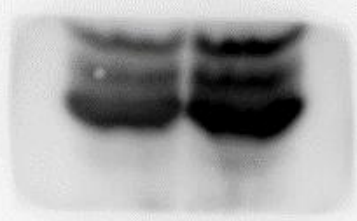

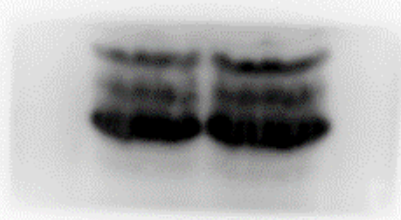

BACE in brain tissue (ND group， HSD group)

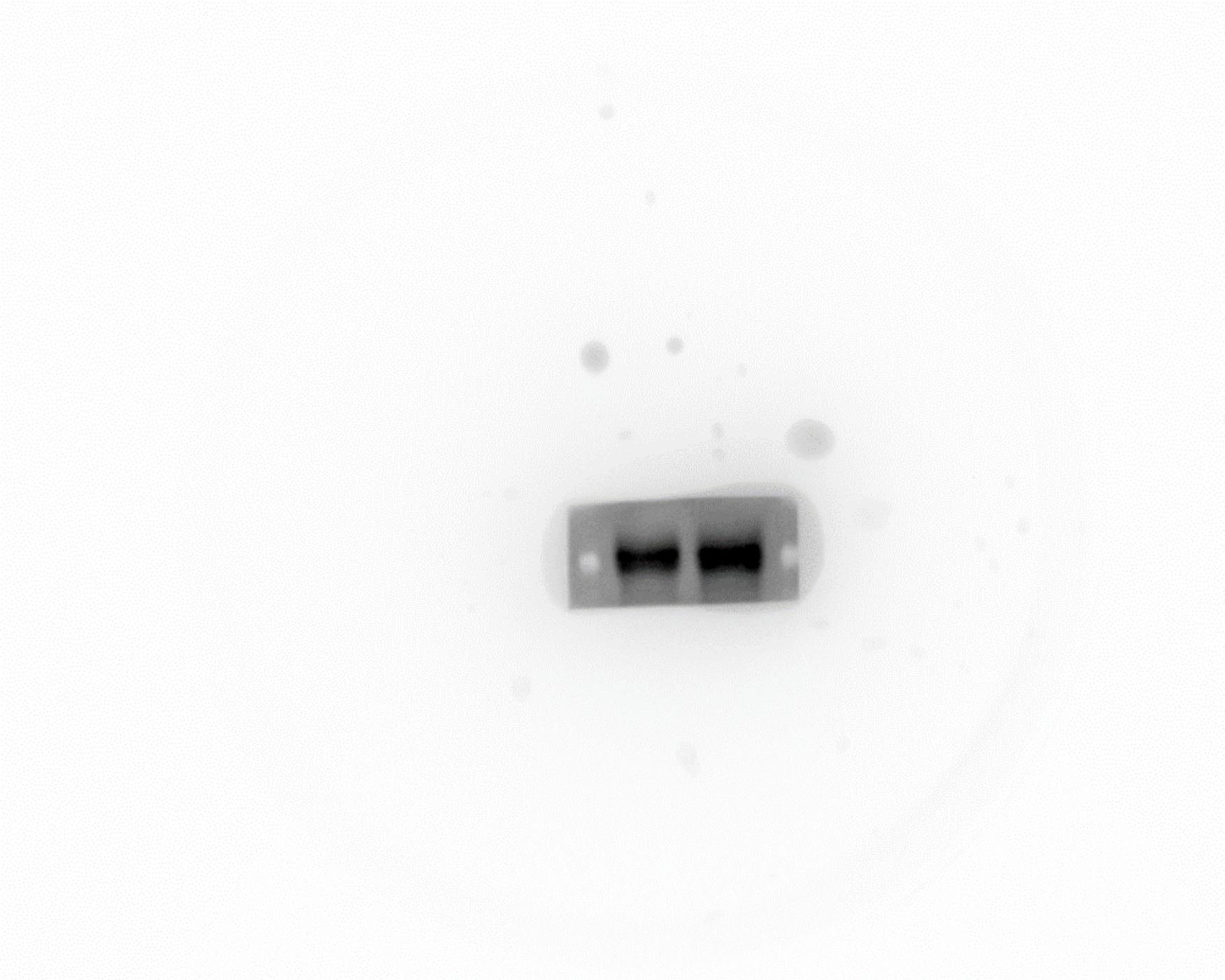

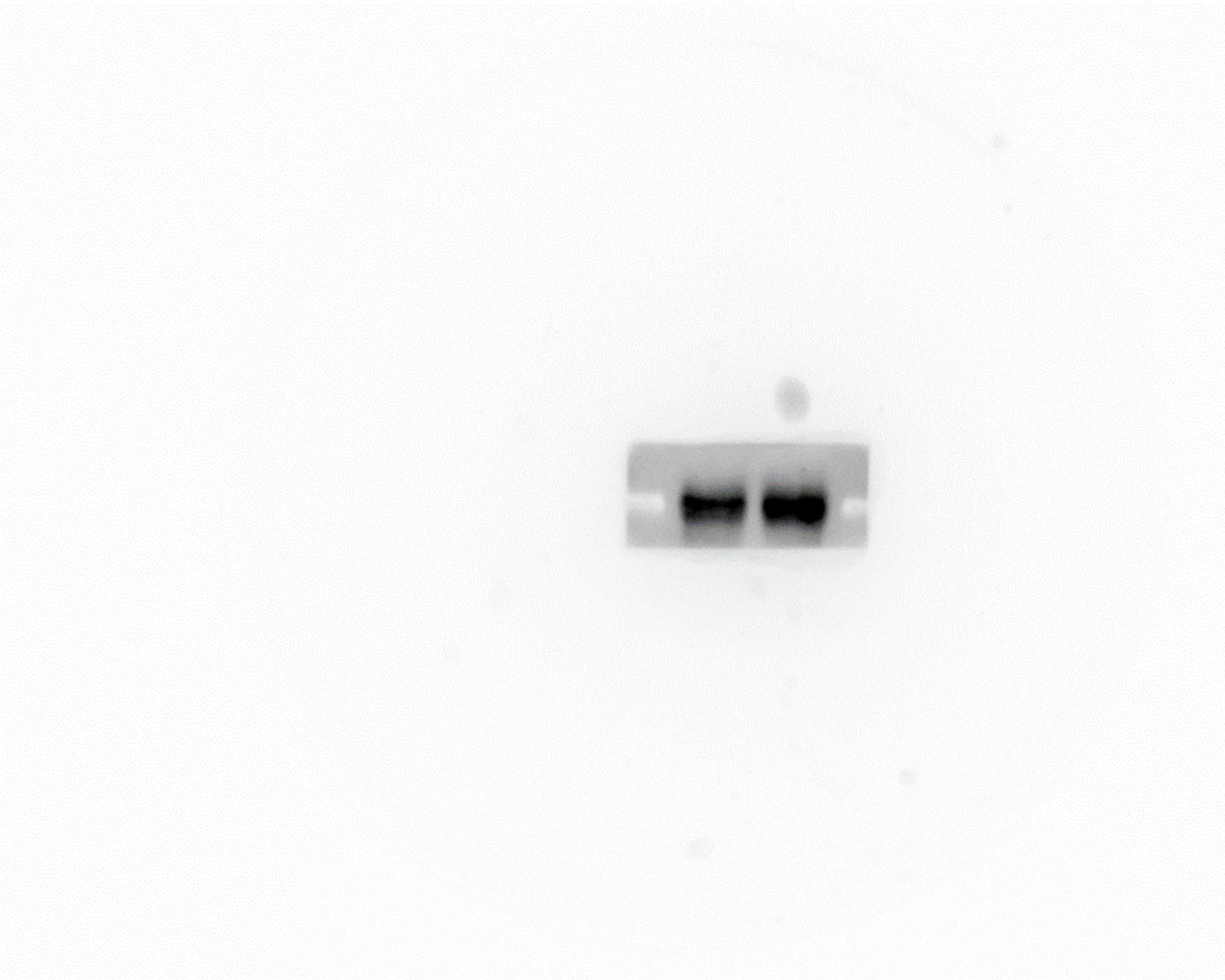

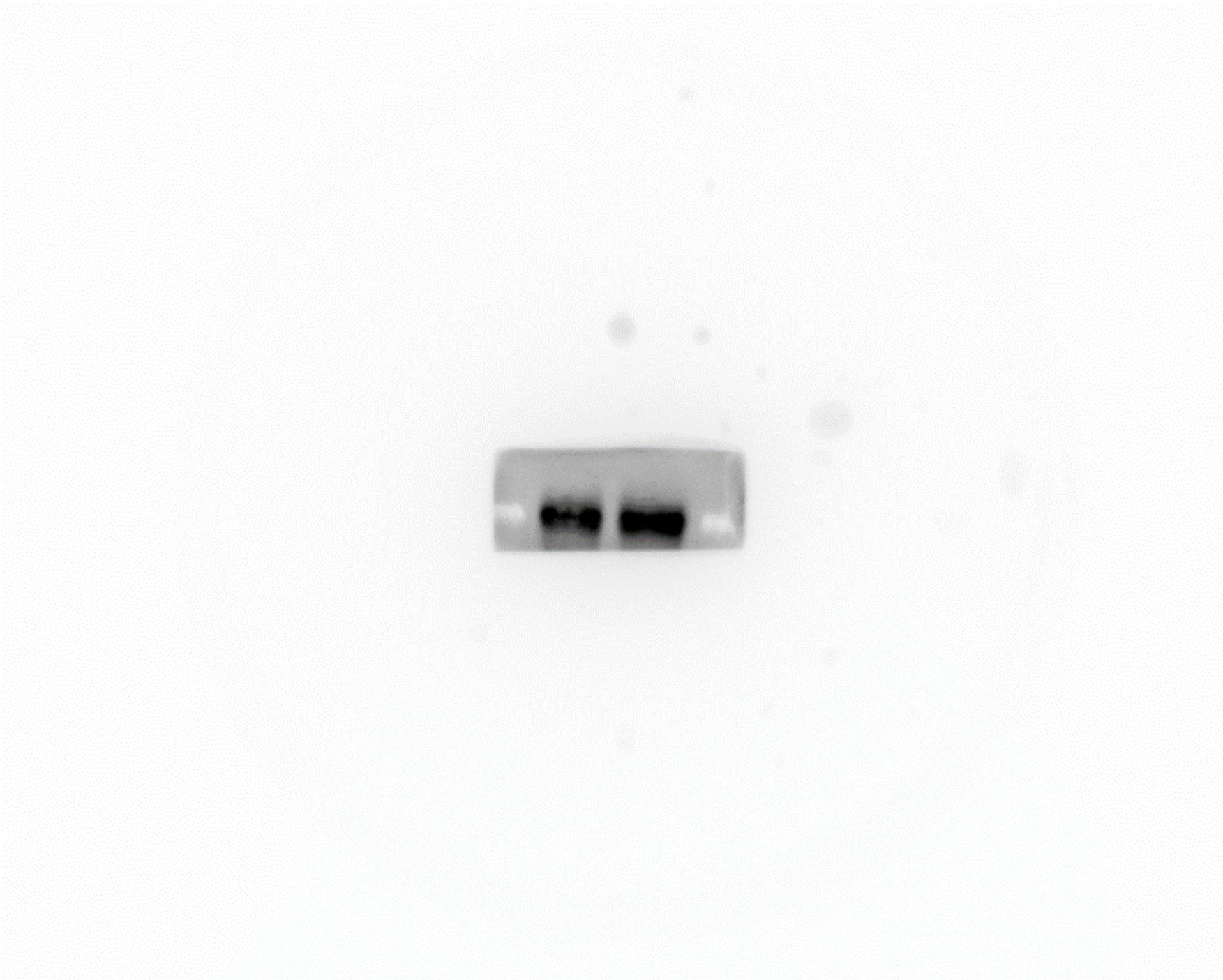

Neprilysin in brain tissue (ND group，HSD group)

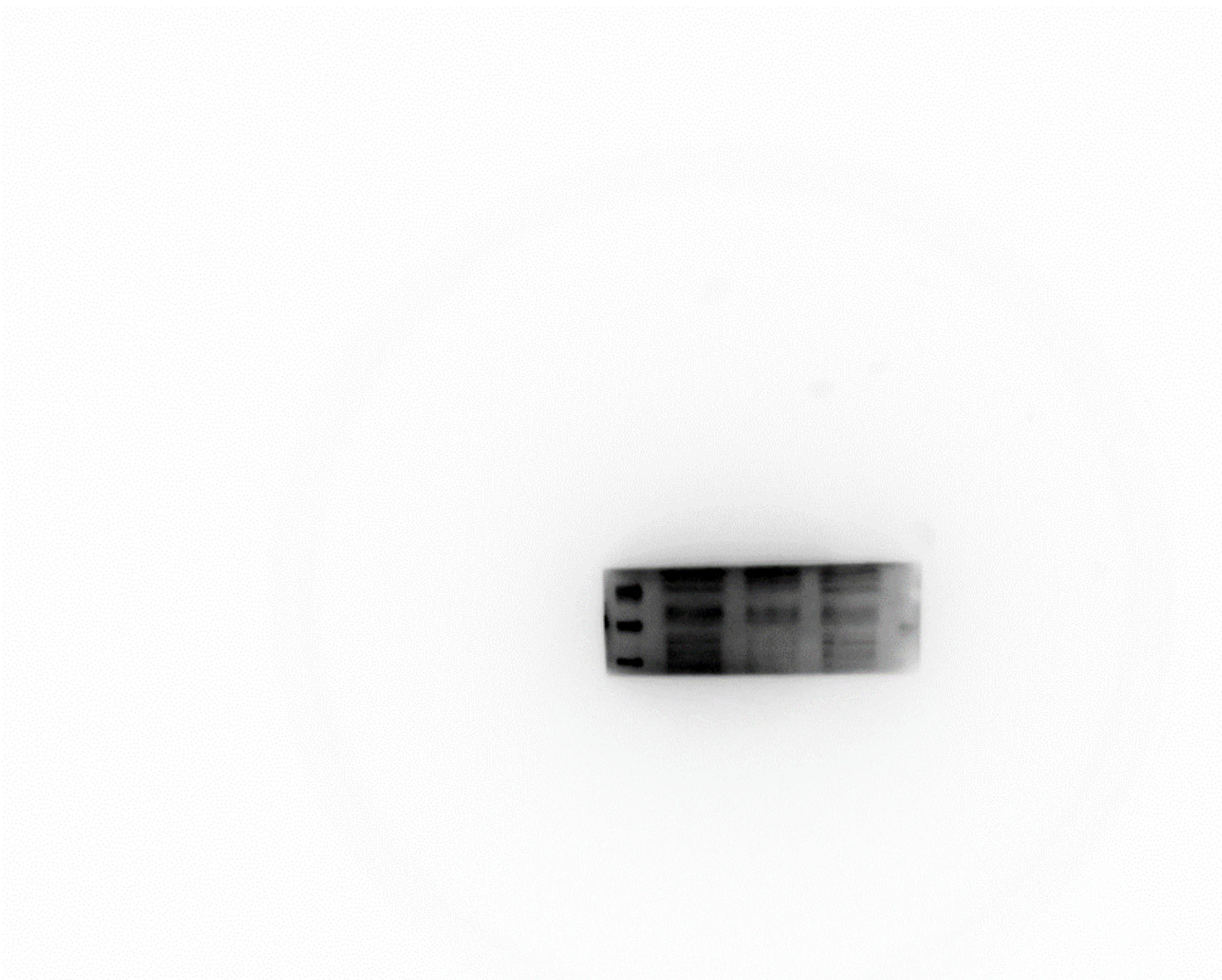

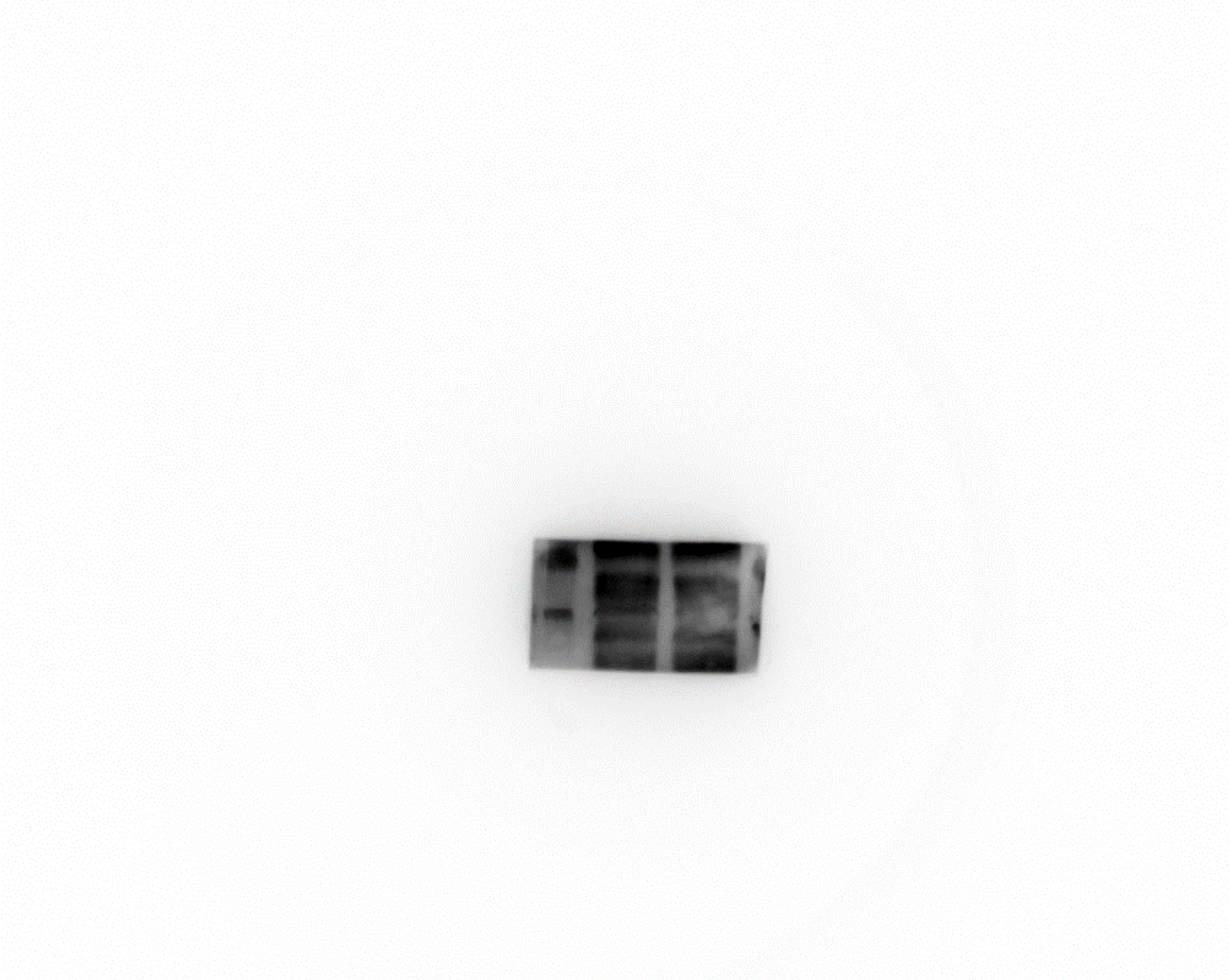

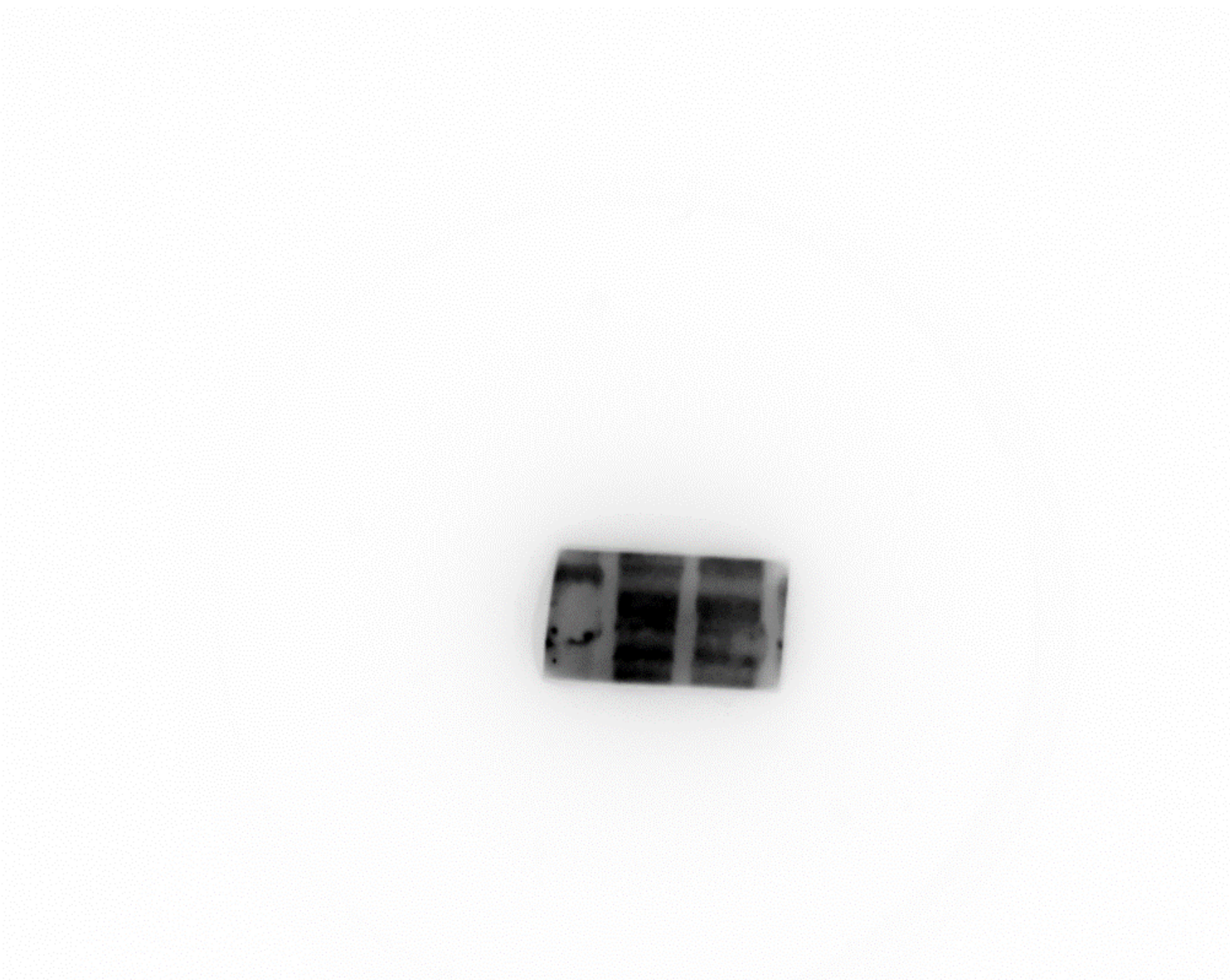

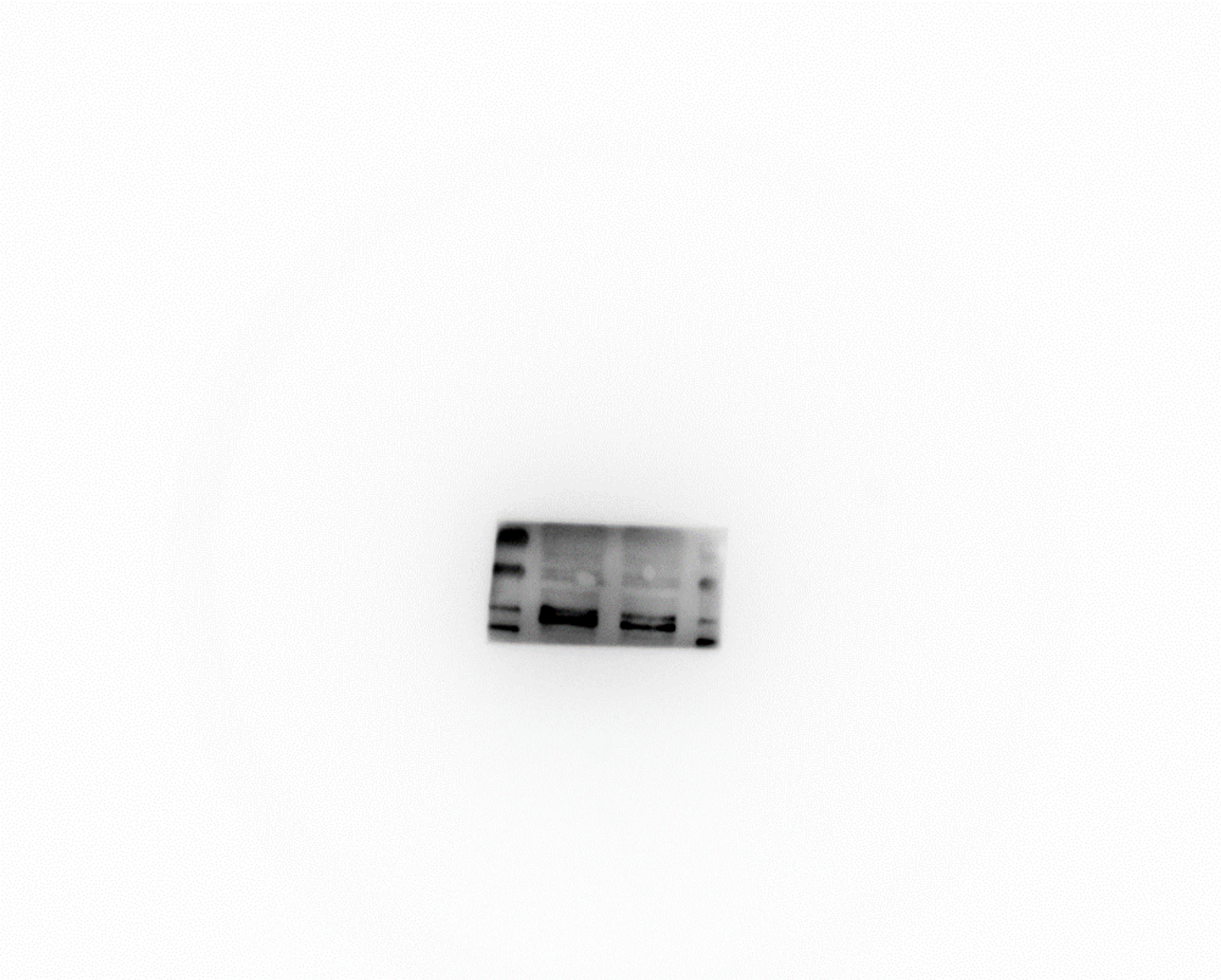

Presenilin1 in brain tissue (ND group, HSD group)

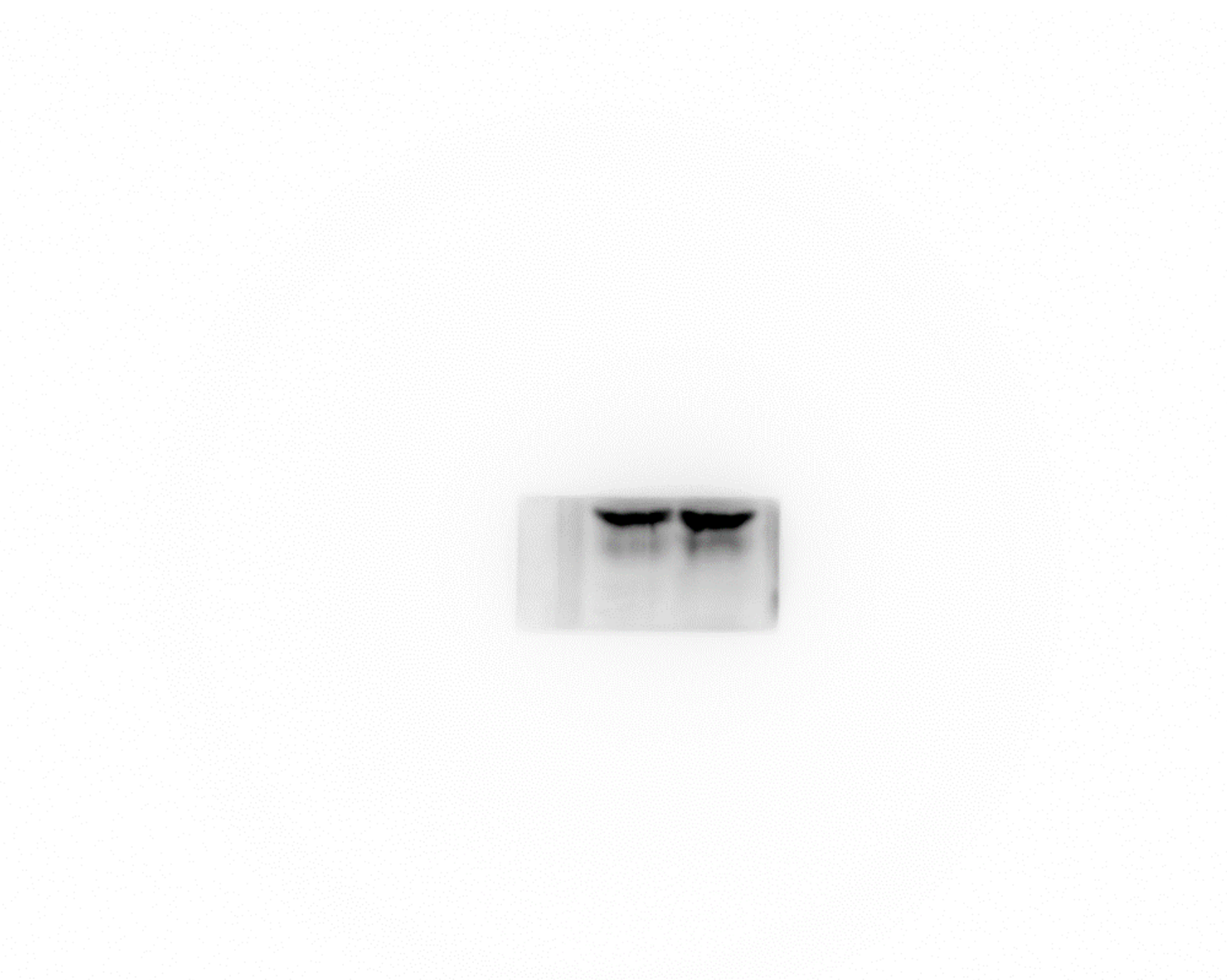

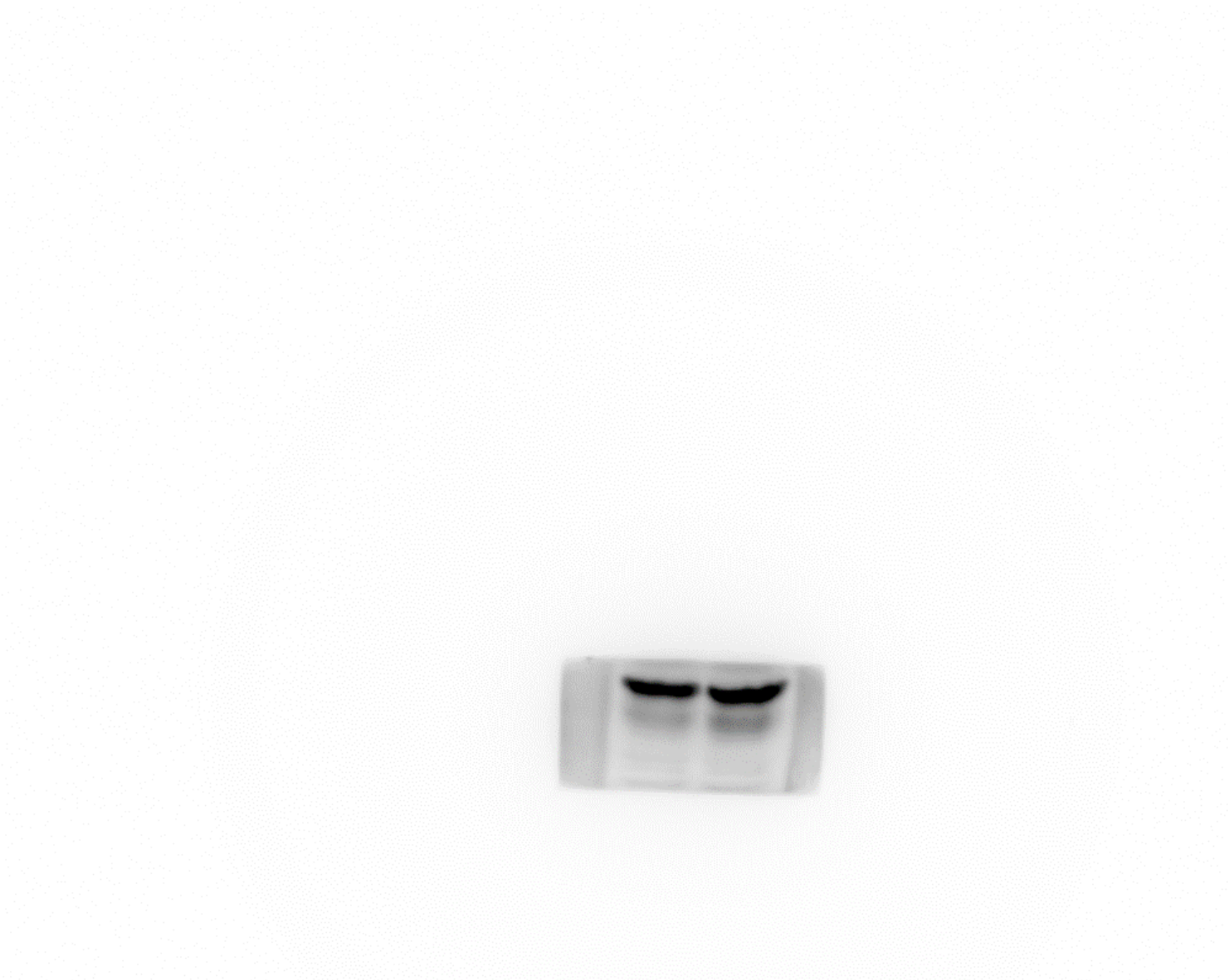

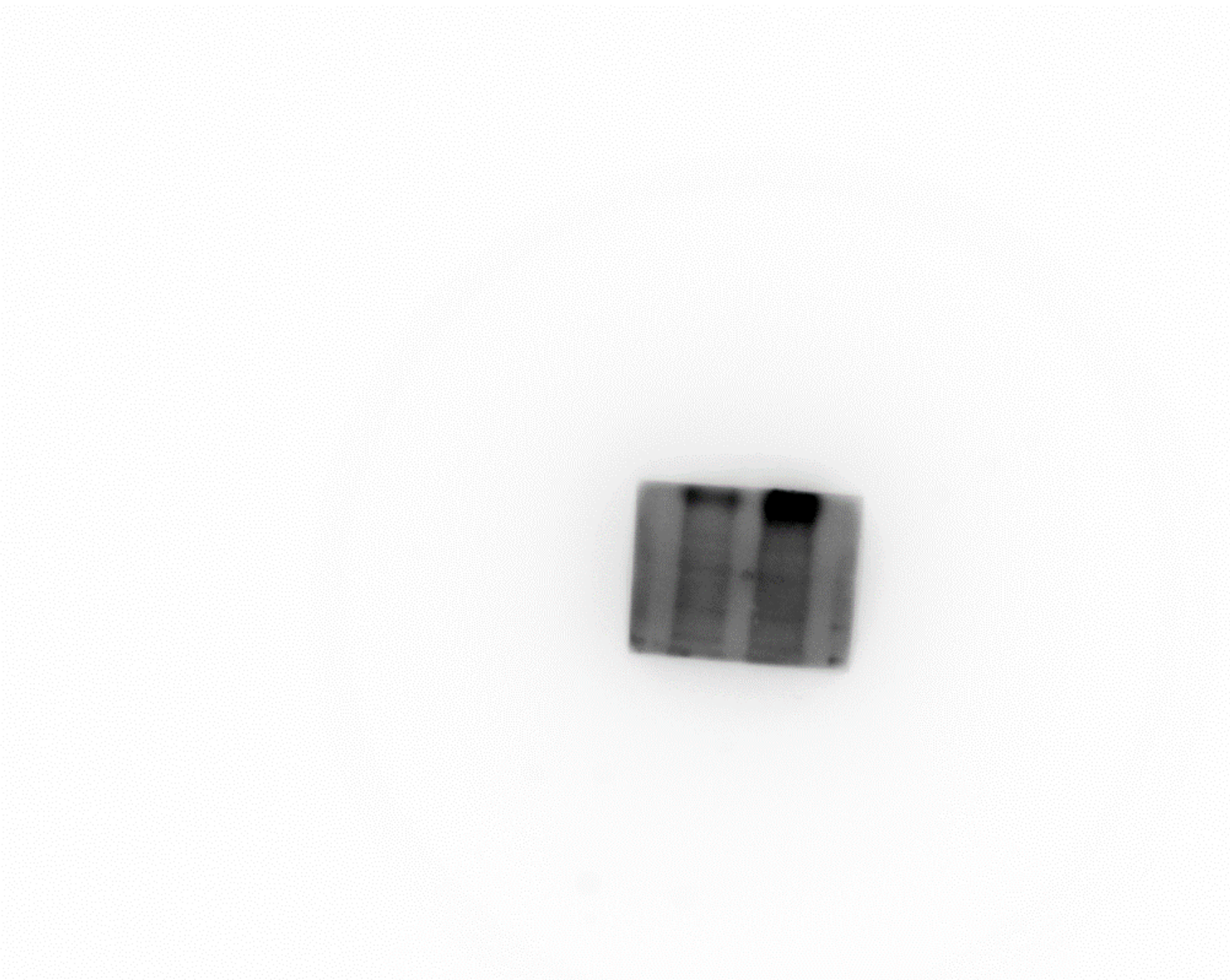

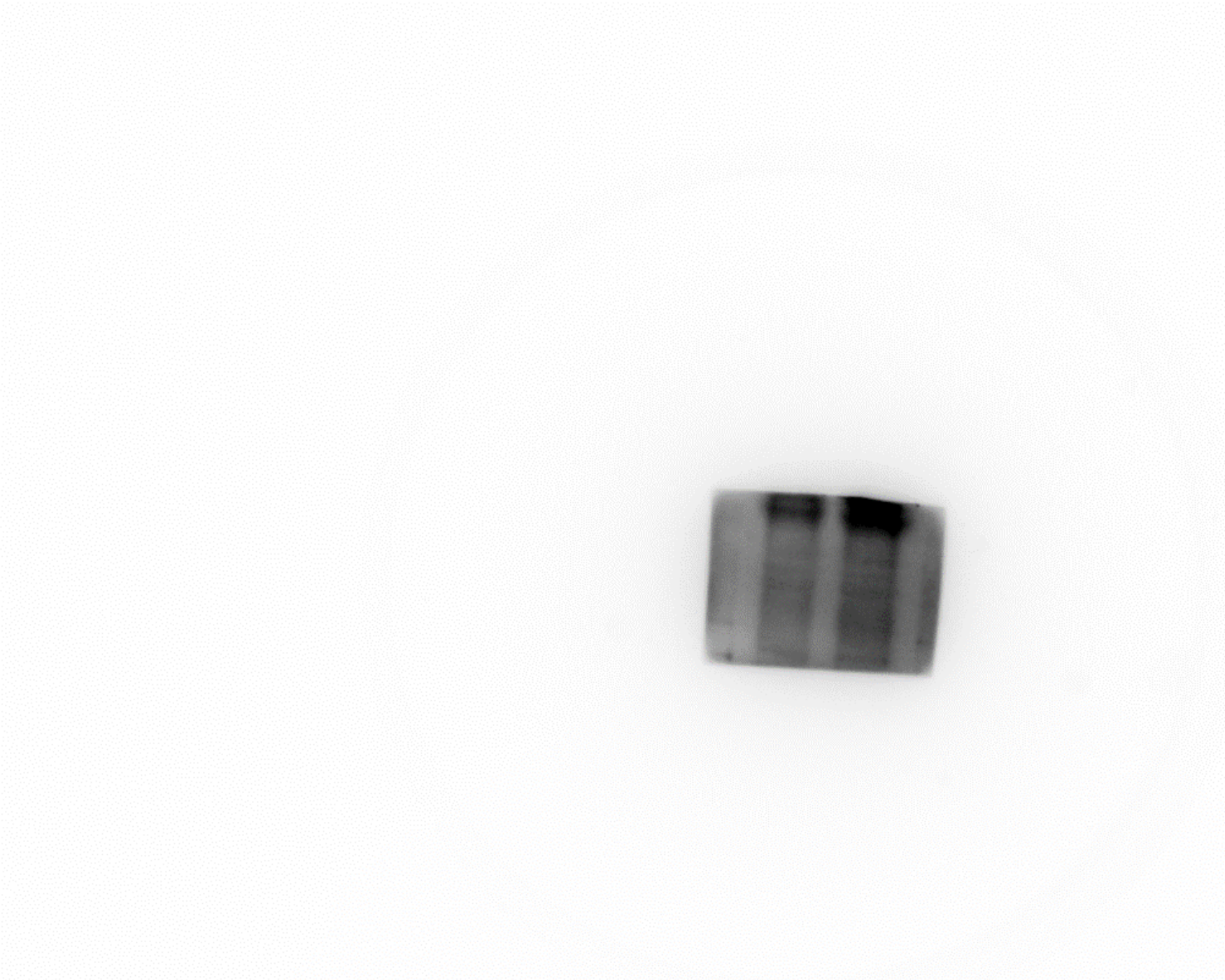

Presenilin2 in brain tissue (ND group，HSD group)

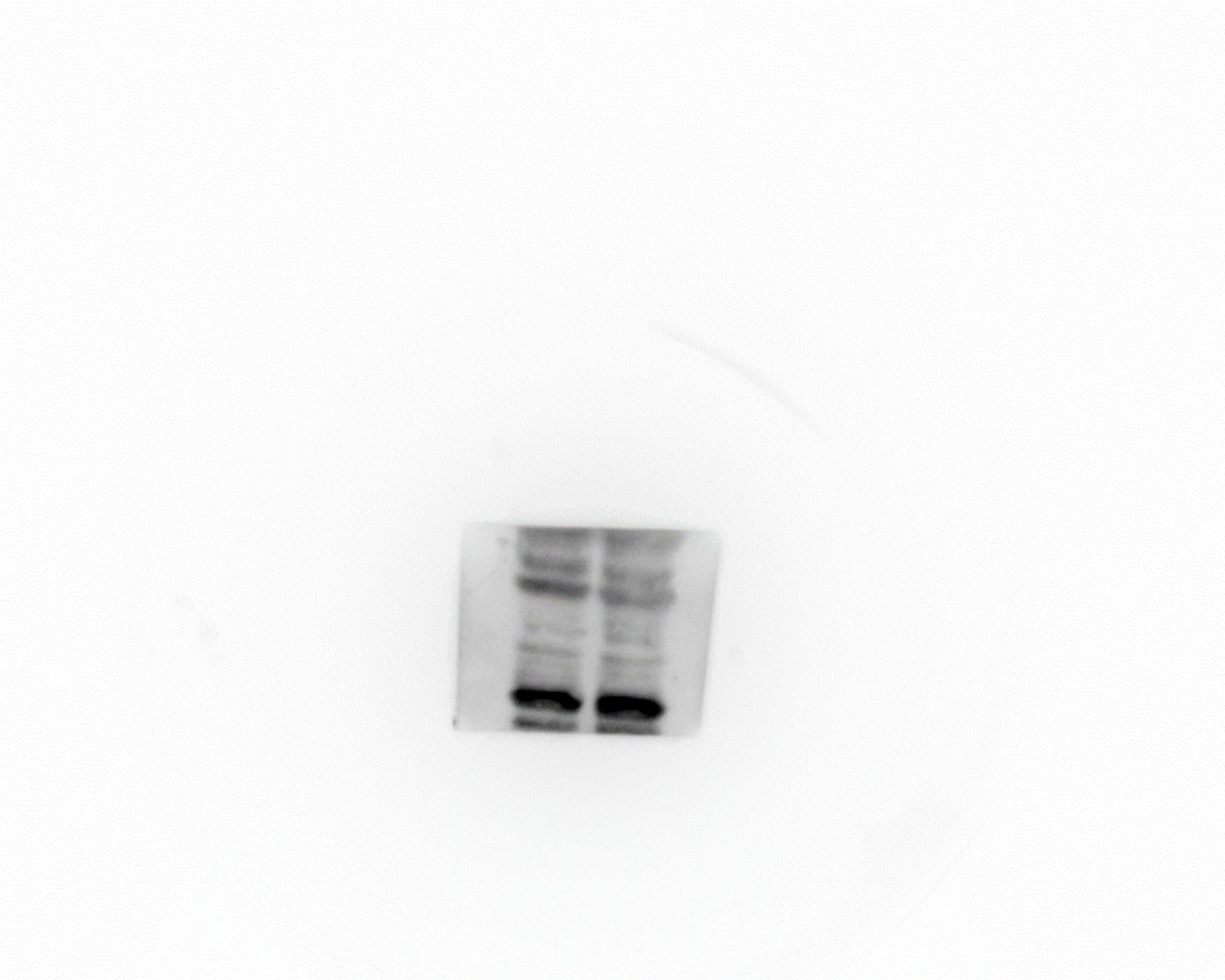

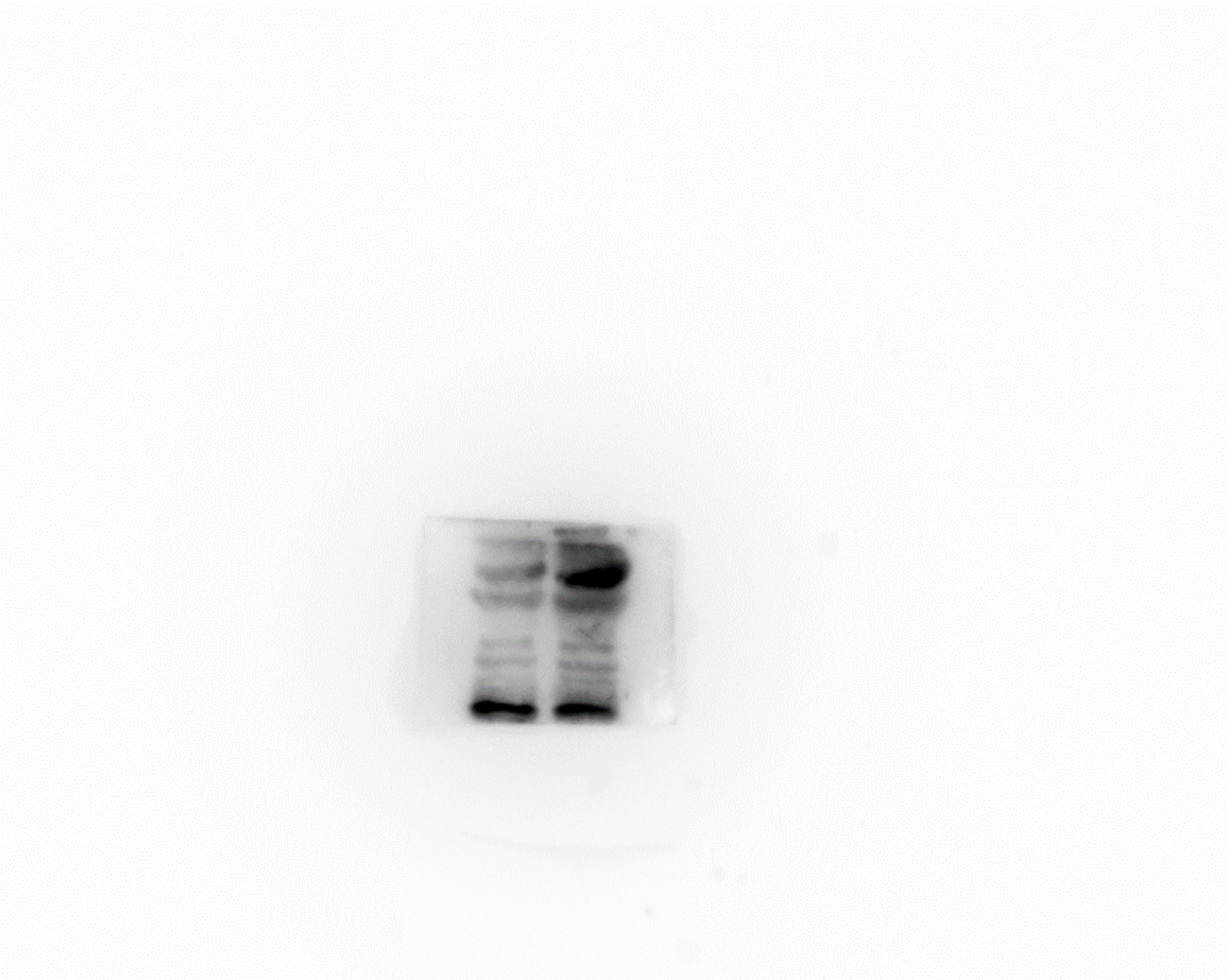

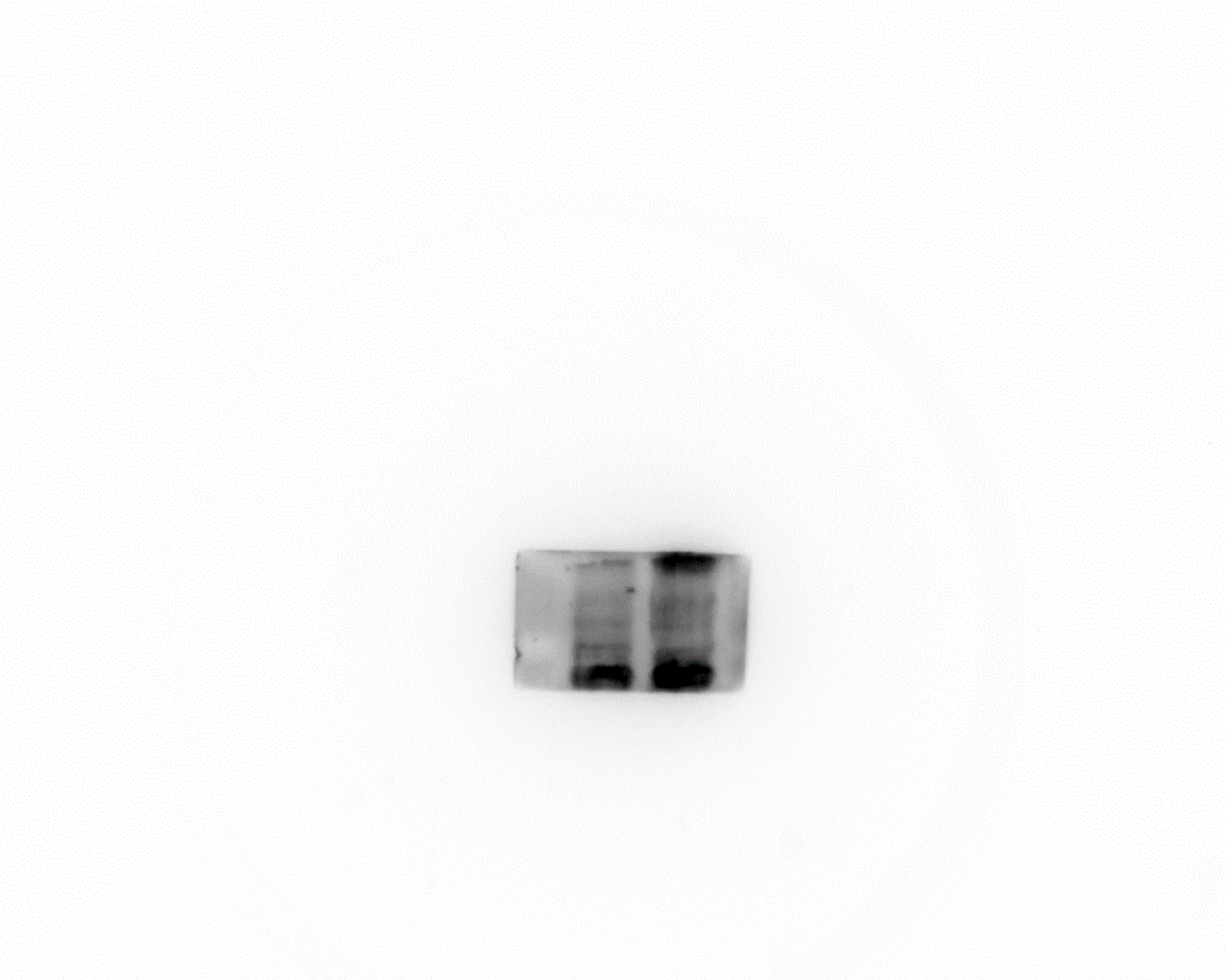

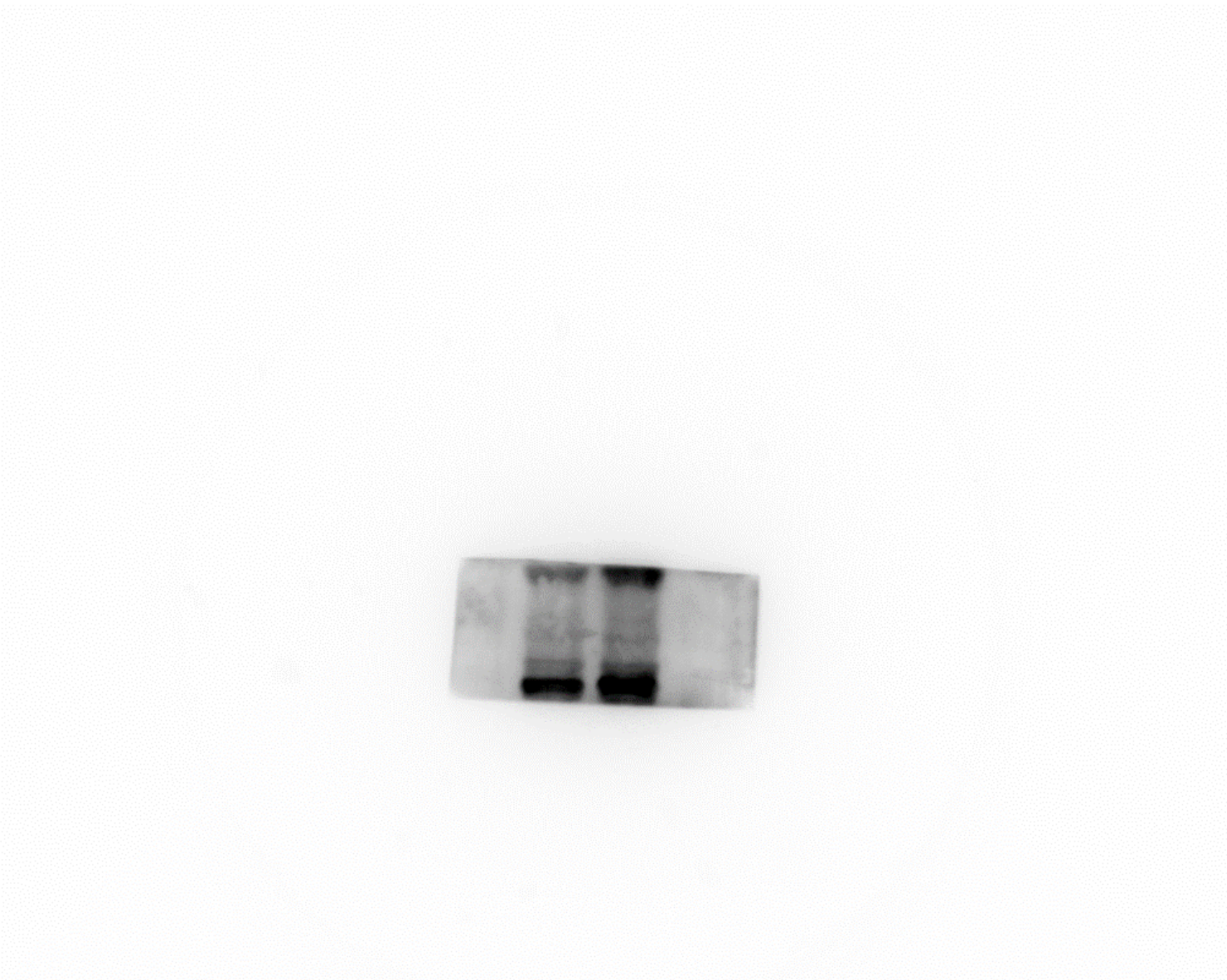

Aβ in brain tissue (ND group，HSD group)

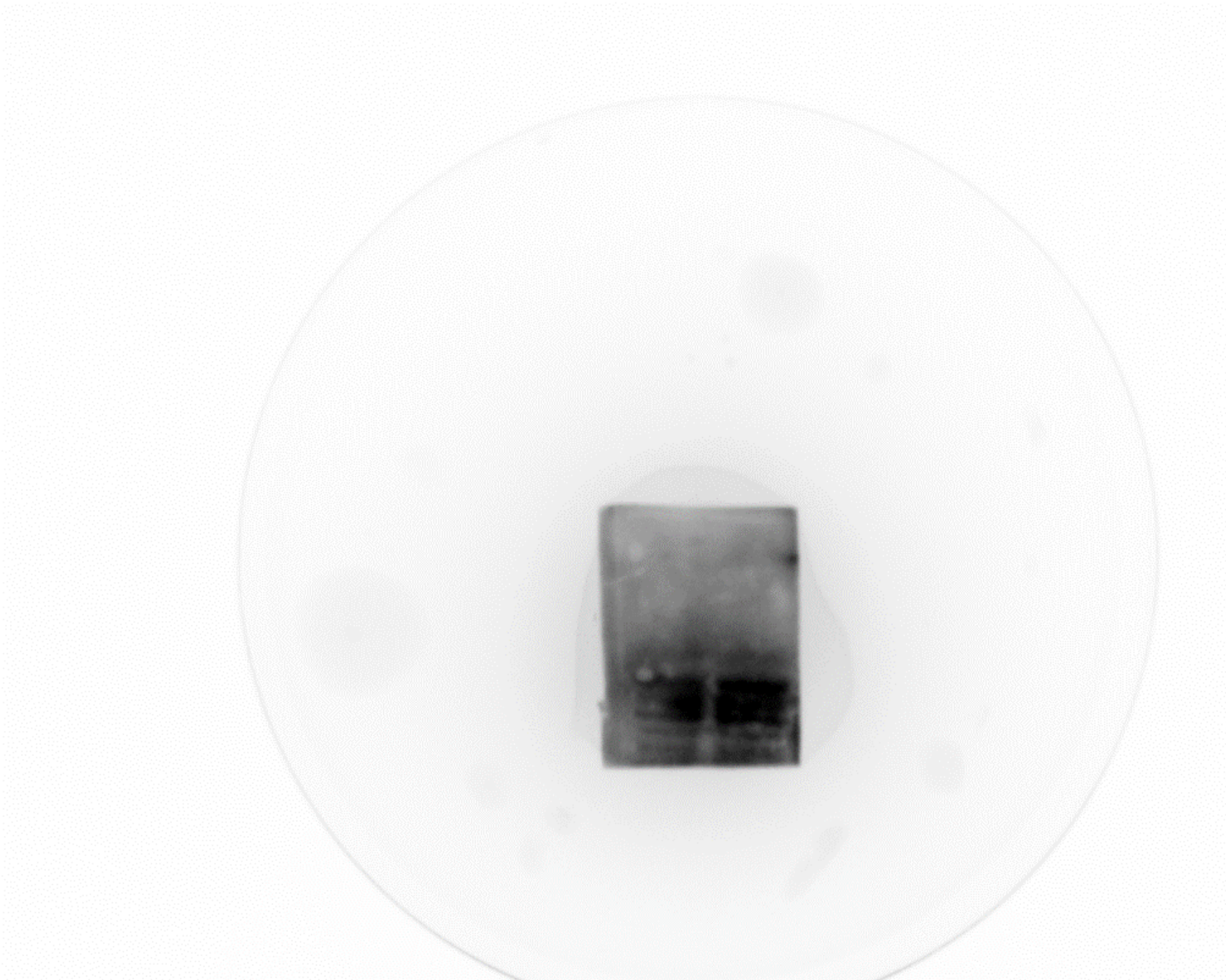

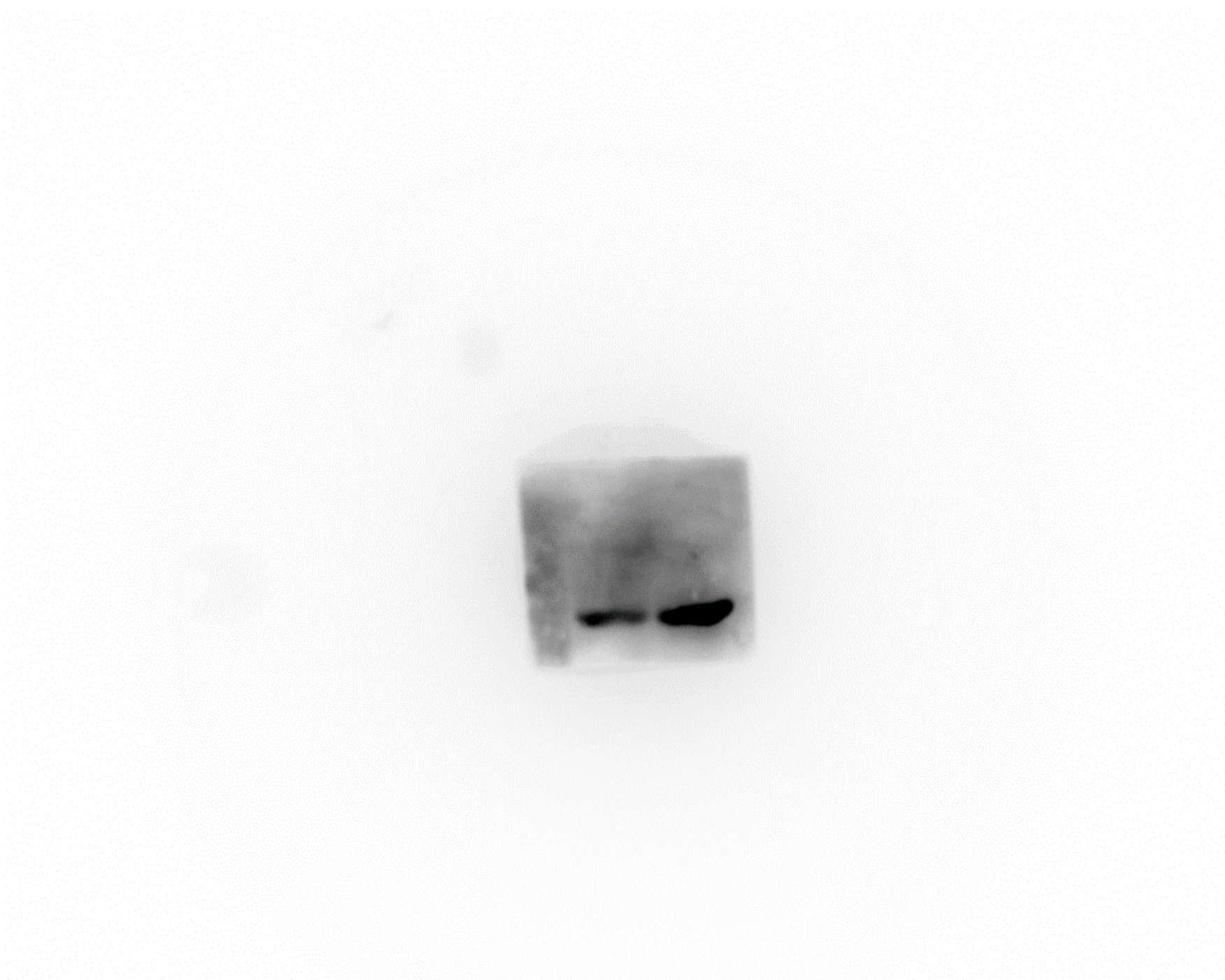

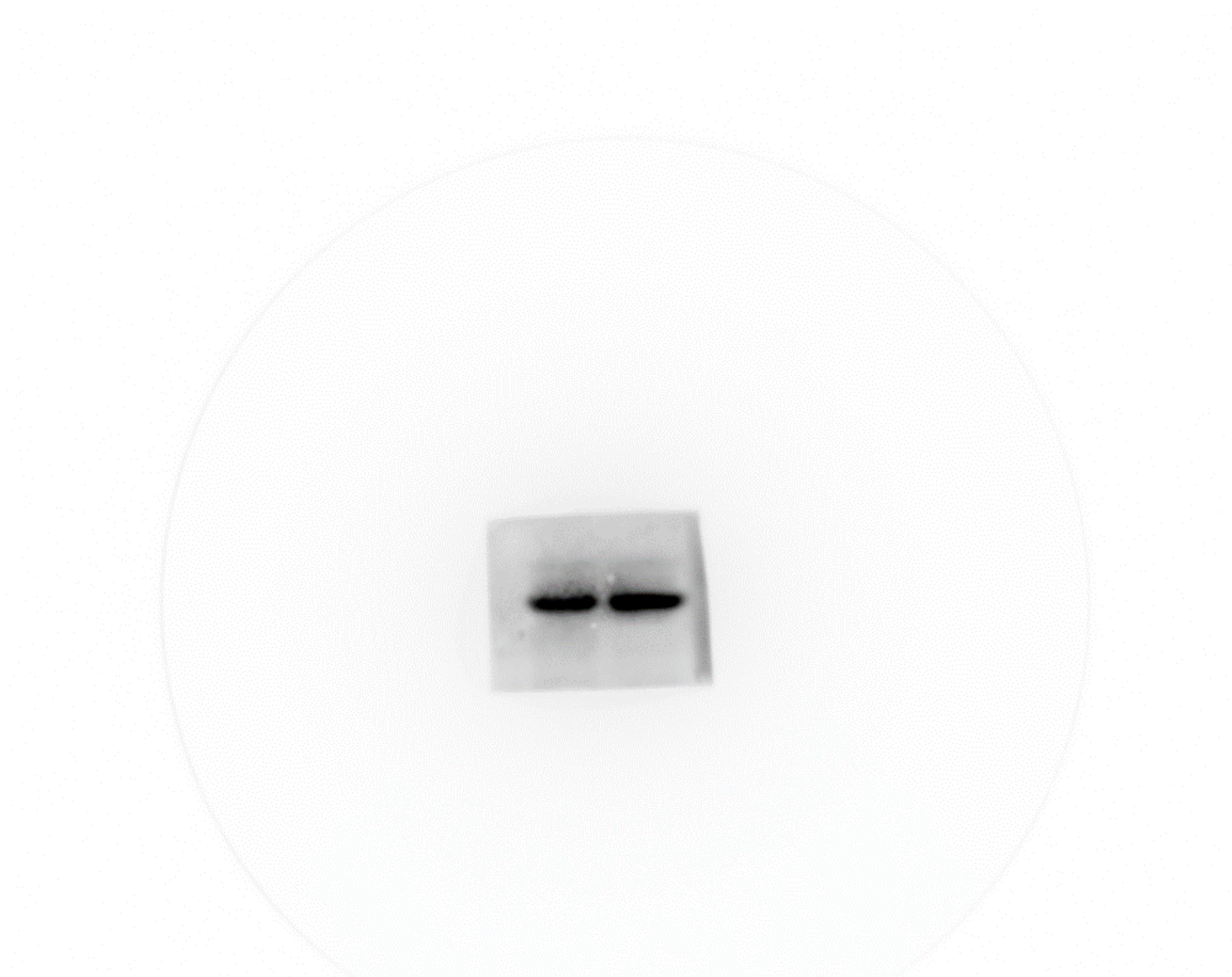

Aβ1-42）in brain tissue (ND group，HSD group)

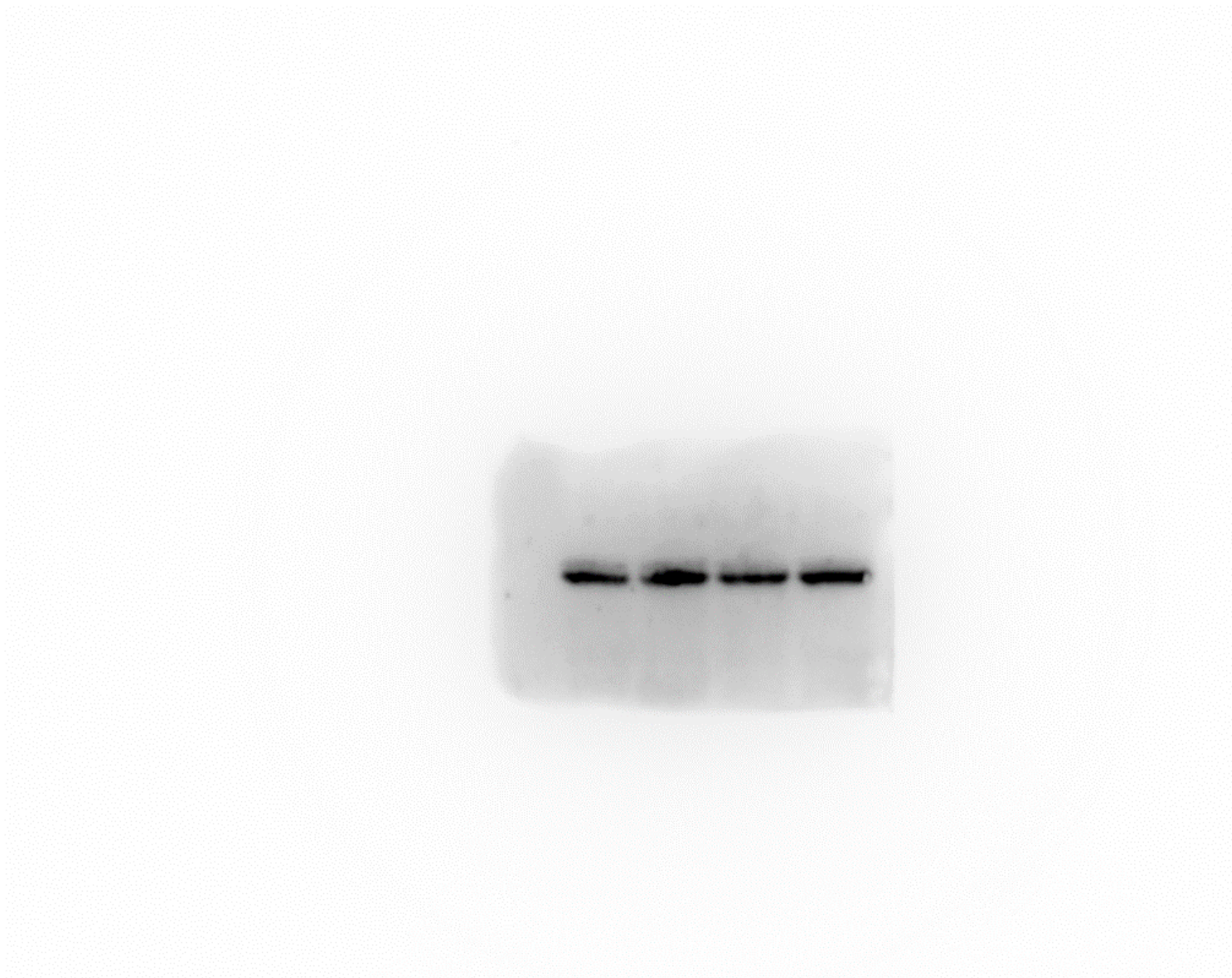

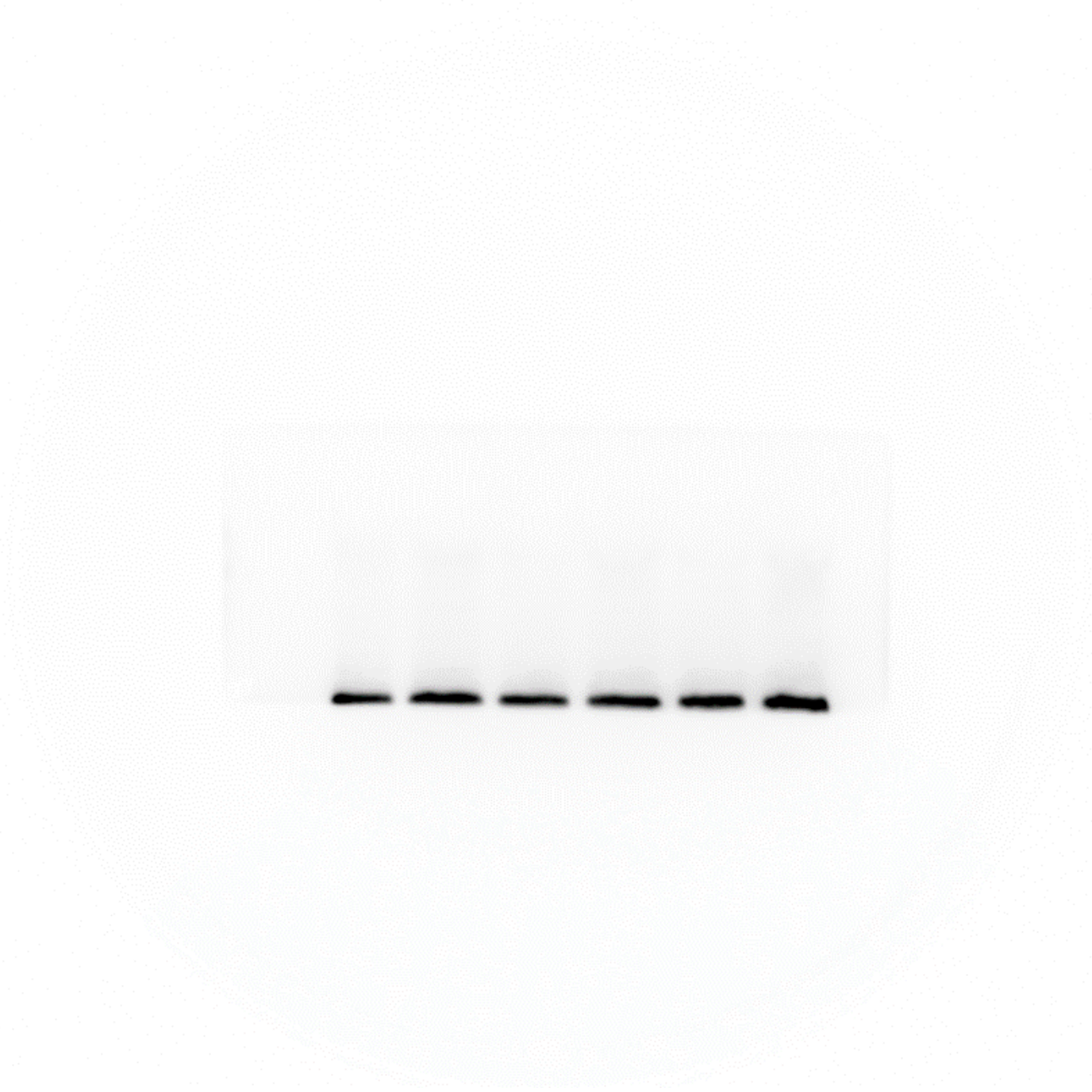

GAPDH in brain tissue (ND group， HSD group)

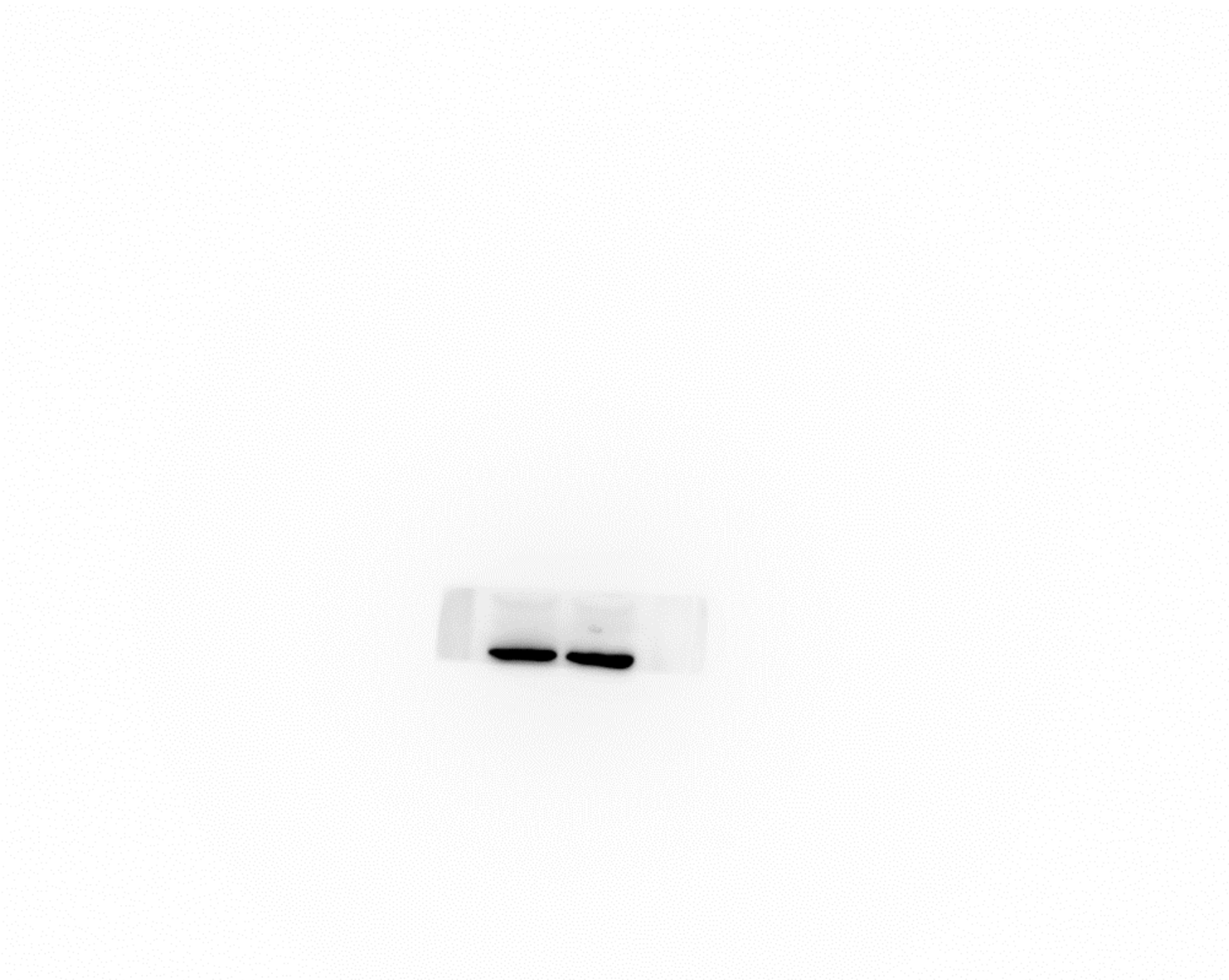

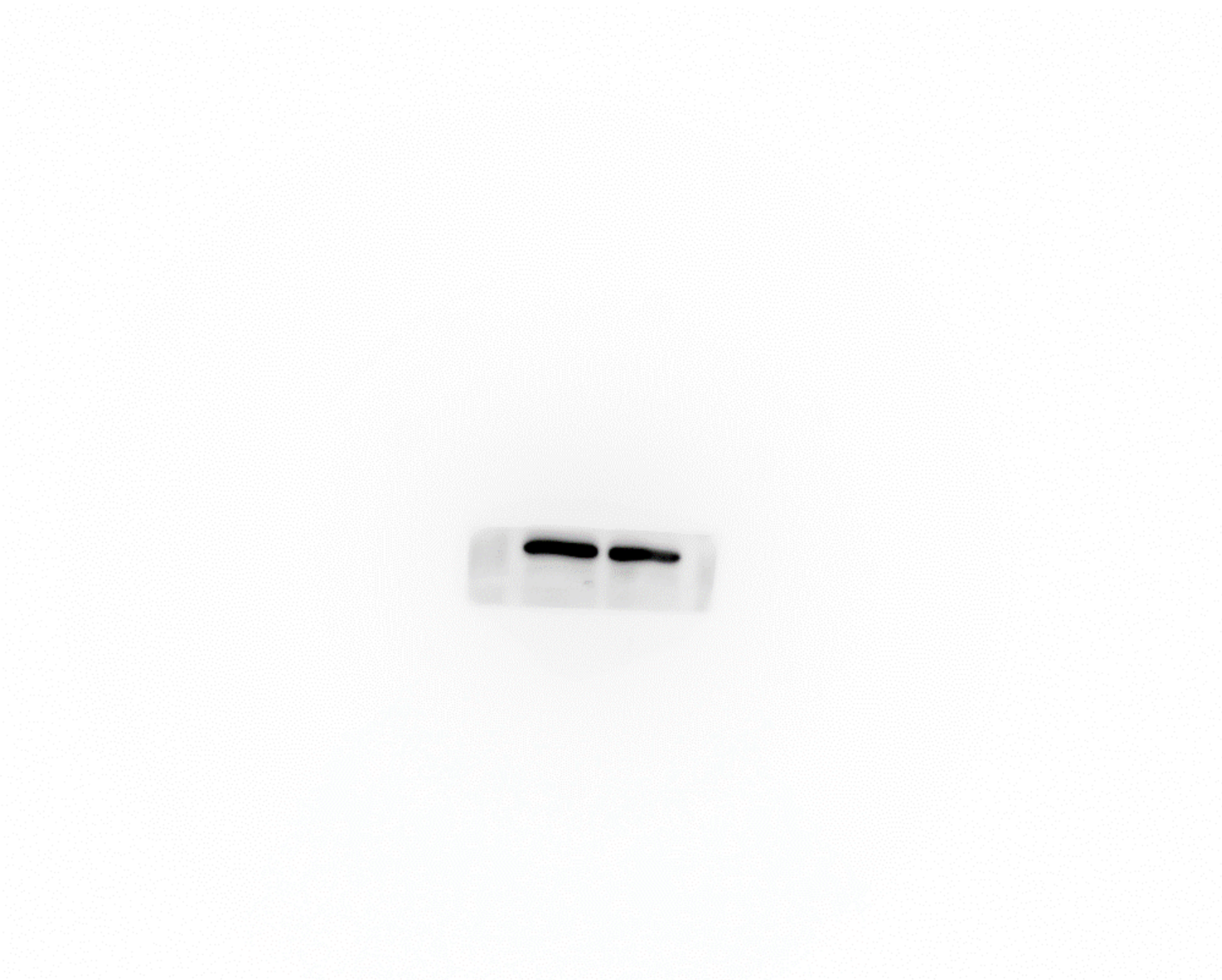

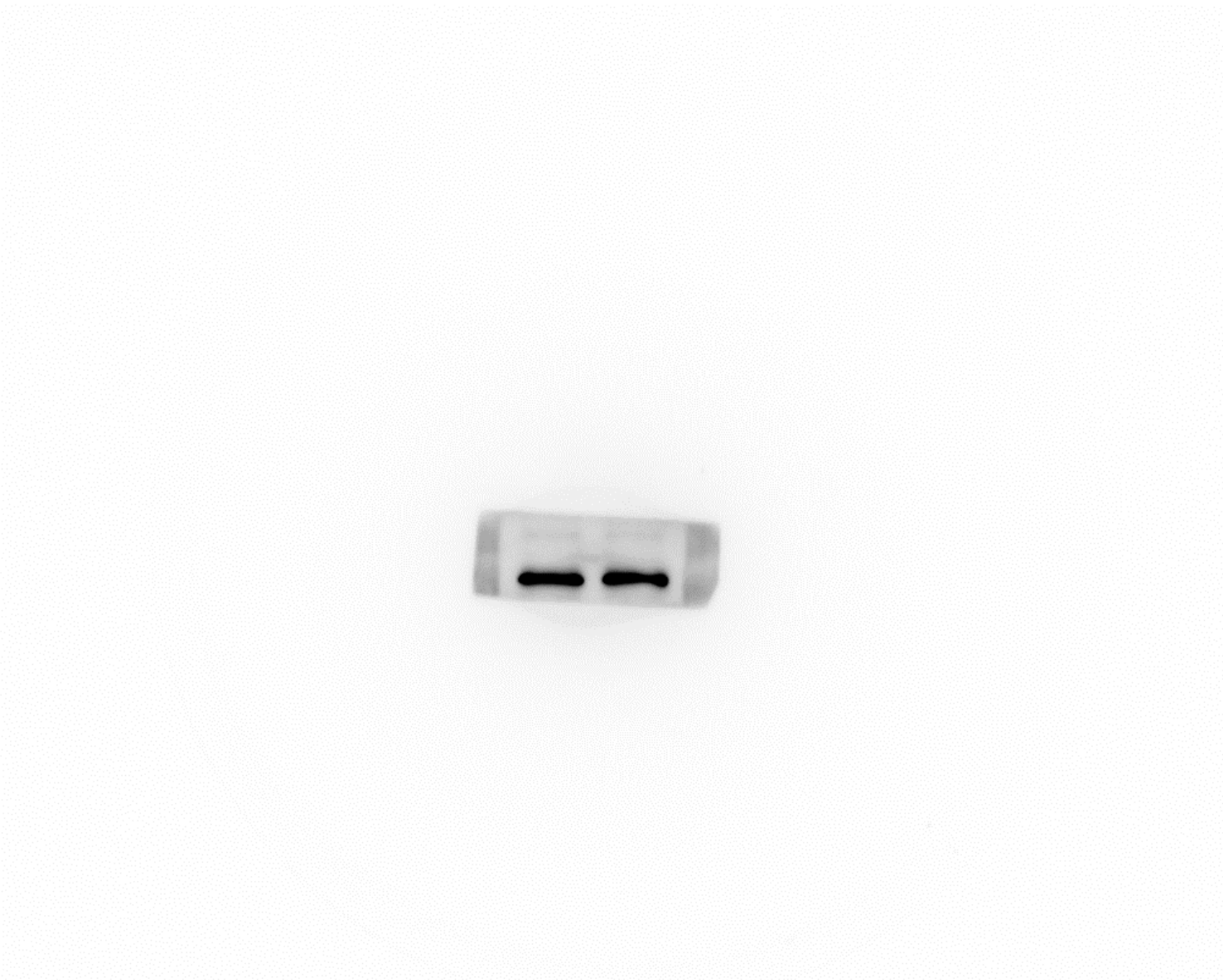

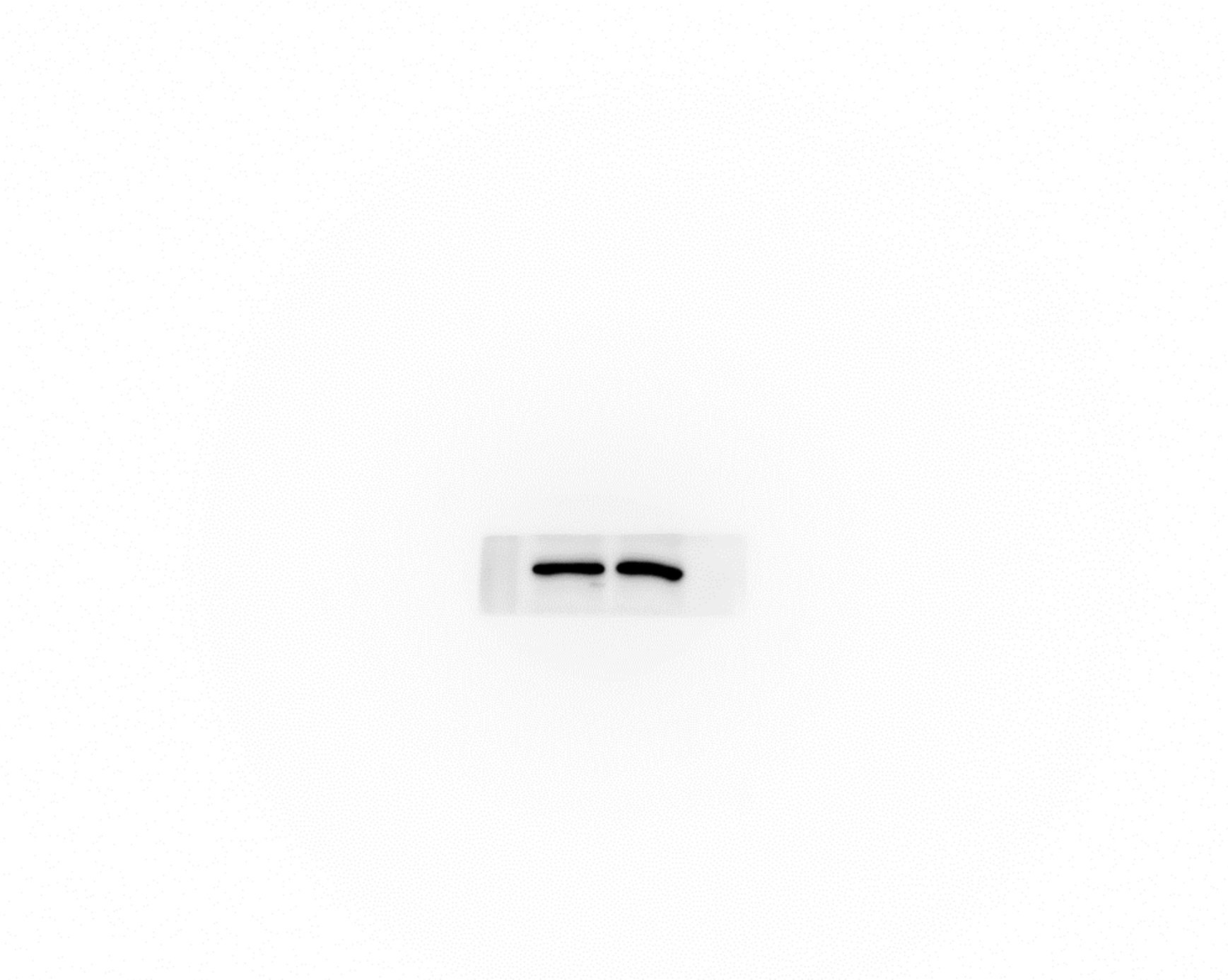

PINK1 in the cell (CON group， NaCl group，NaCl+CCCP group)

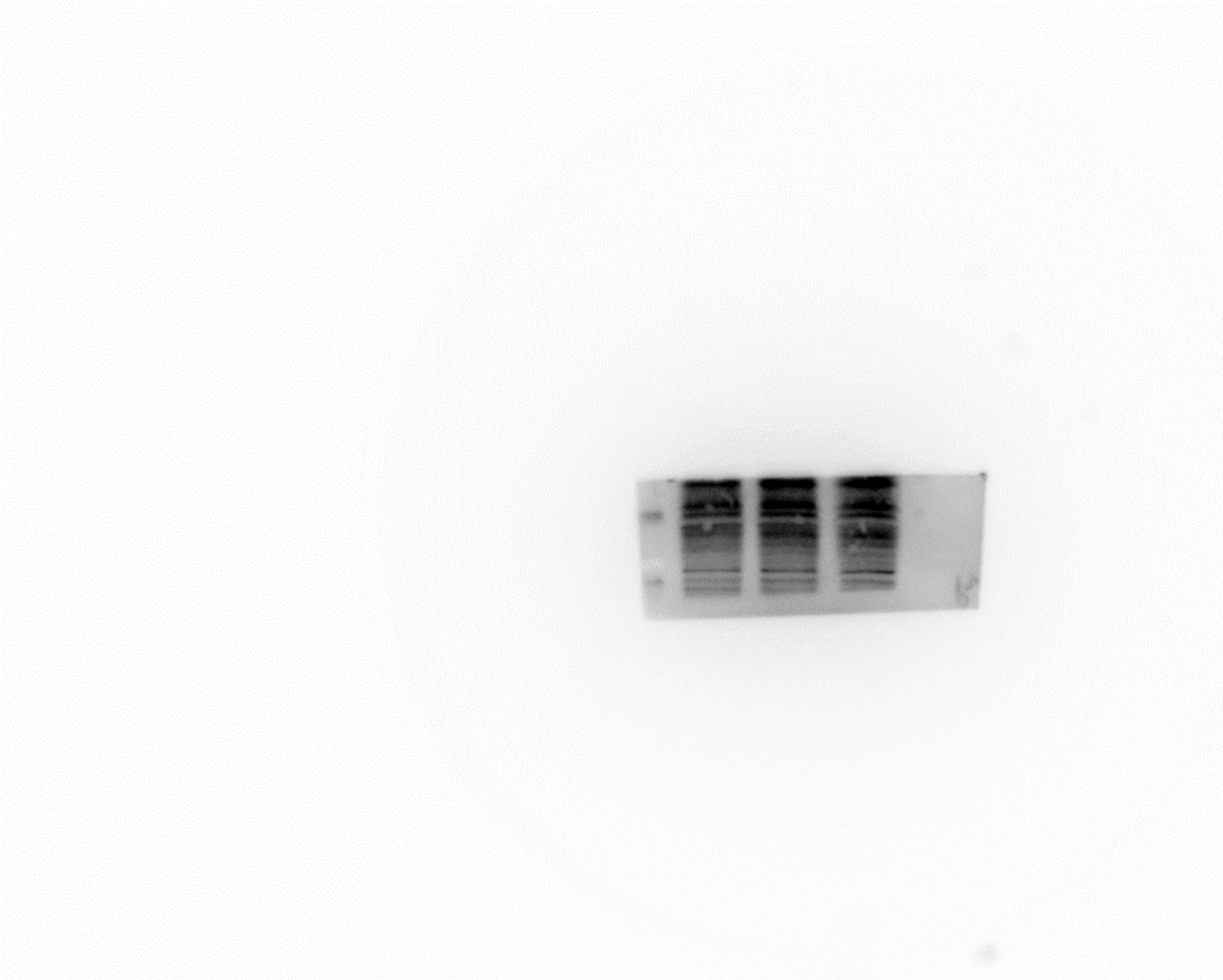

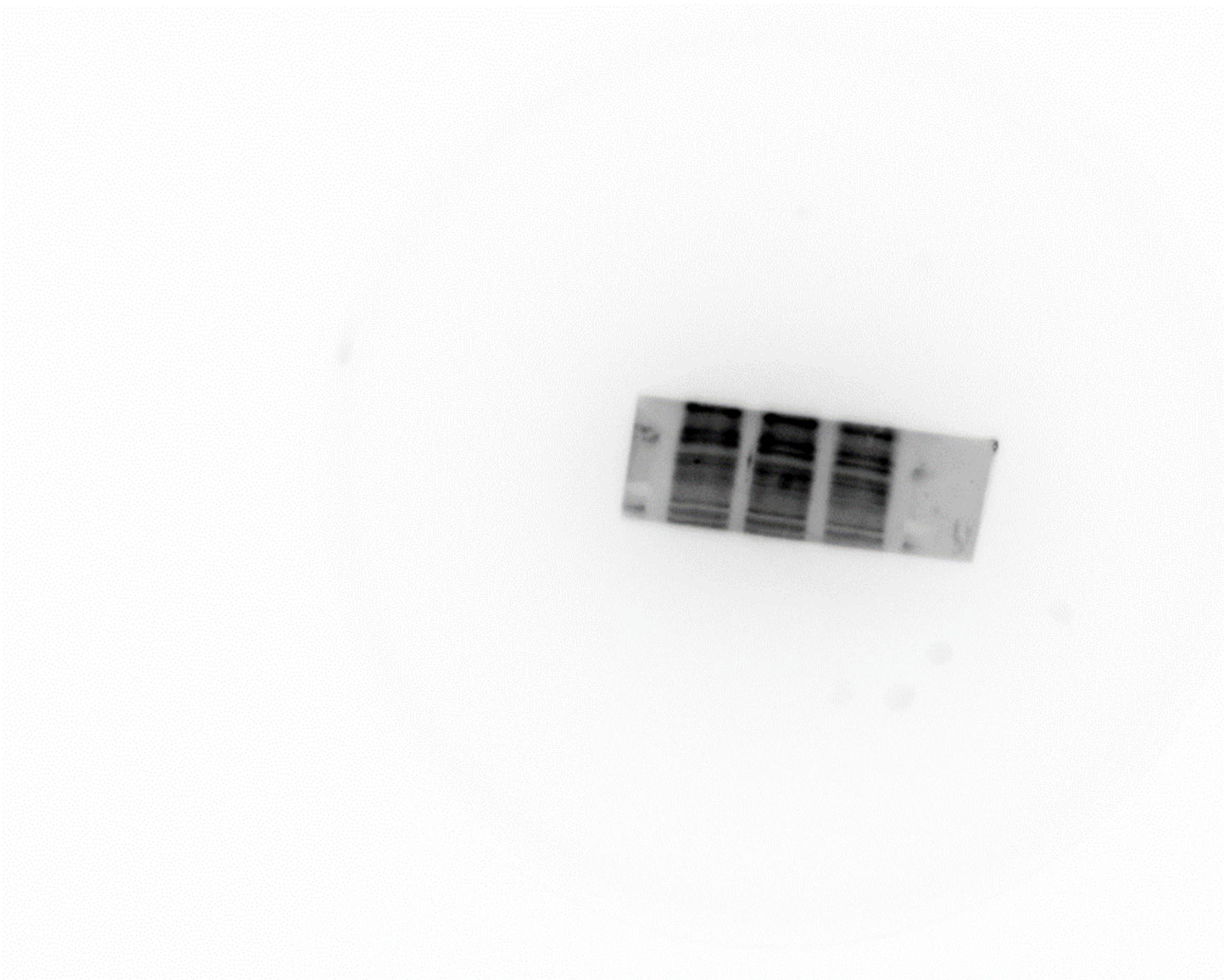

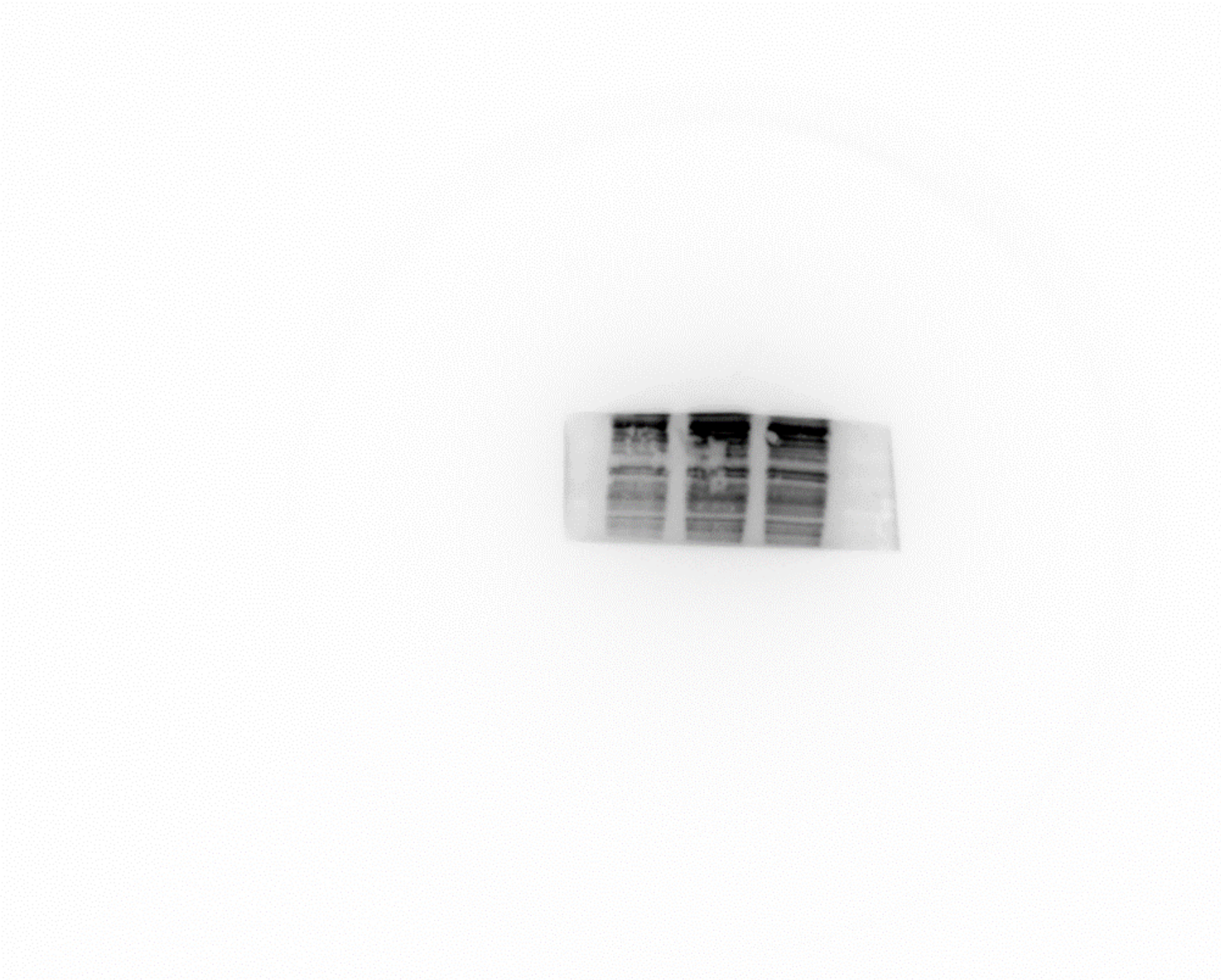

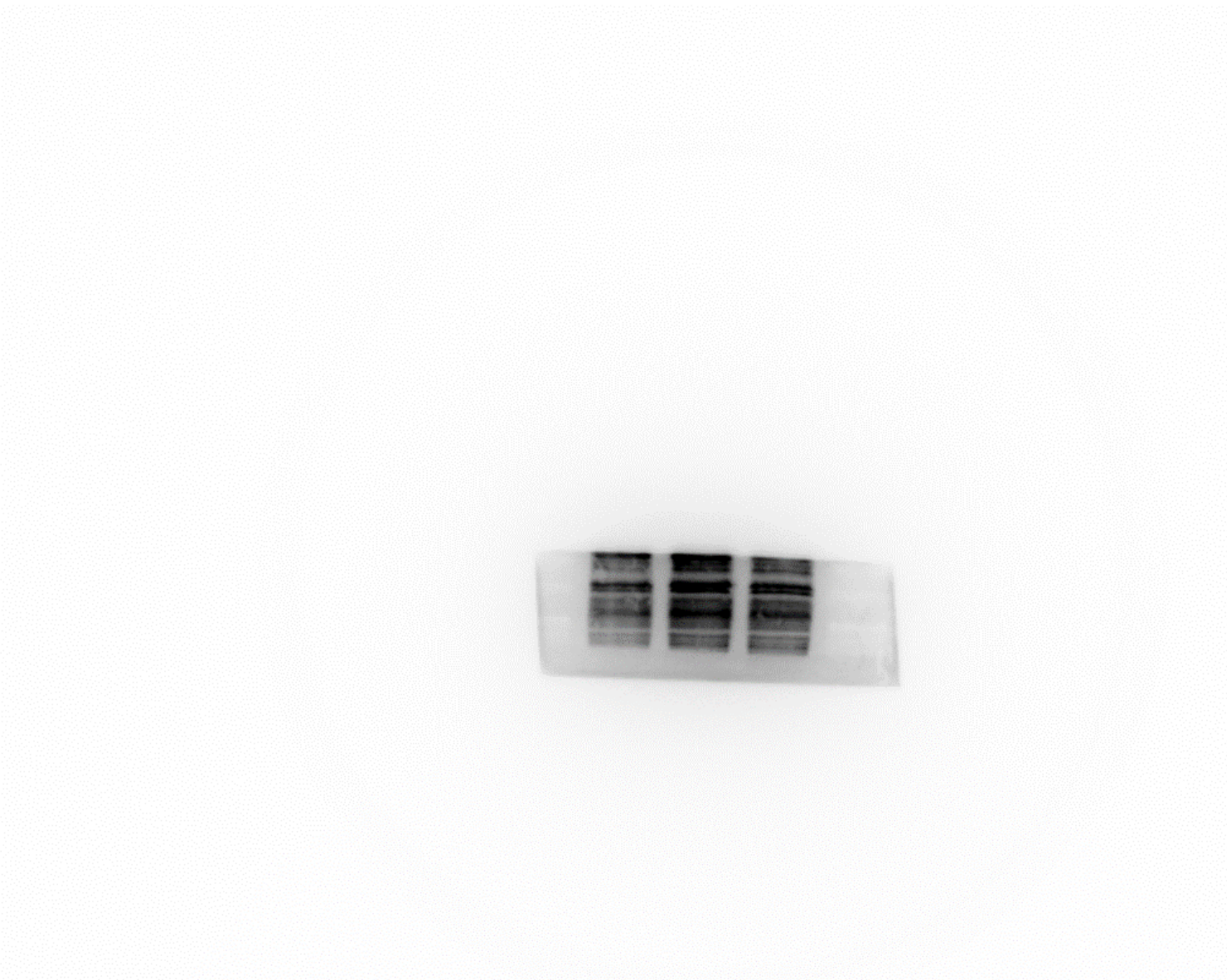

PARKIN in the cell (CON group， NaCl group，NaCl+CCCP group)

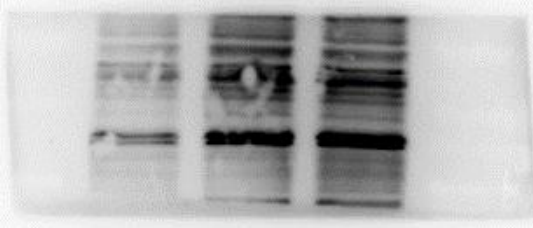

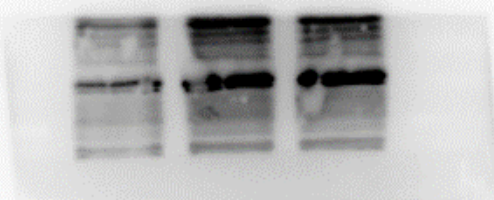

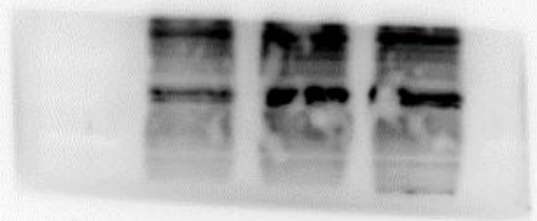

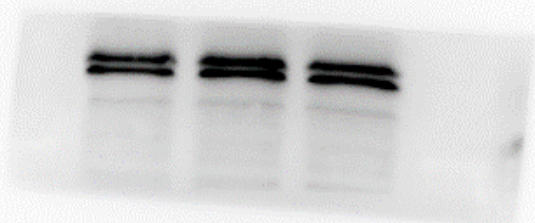

Beclin1 in the cell (CON group， NaCl group， NaCl+CCCP group)

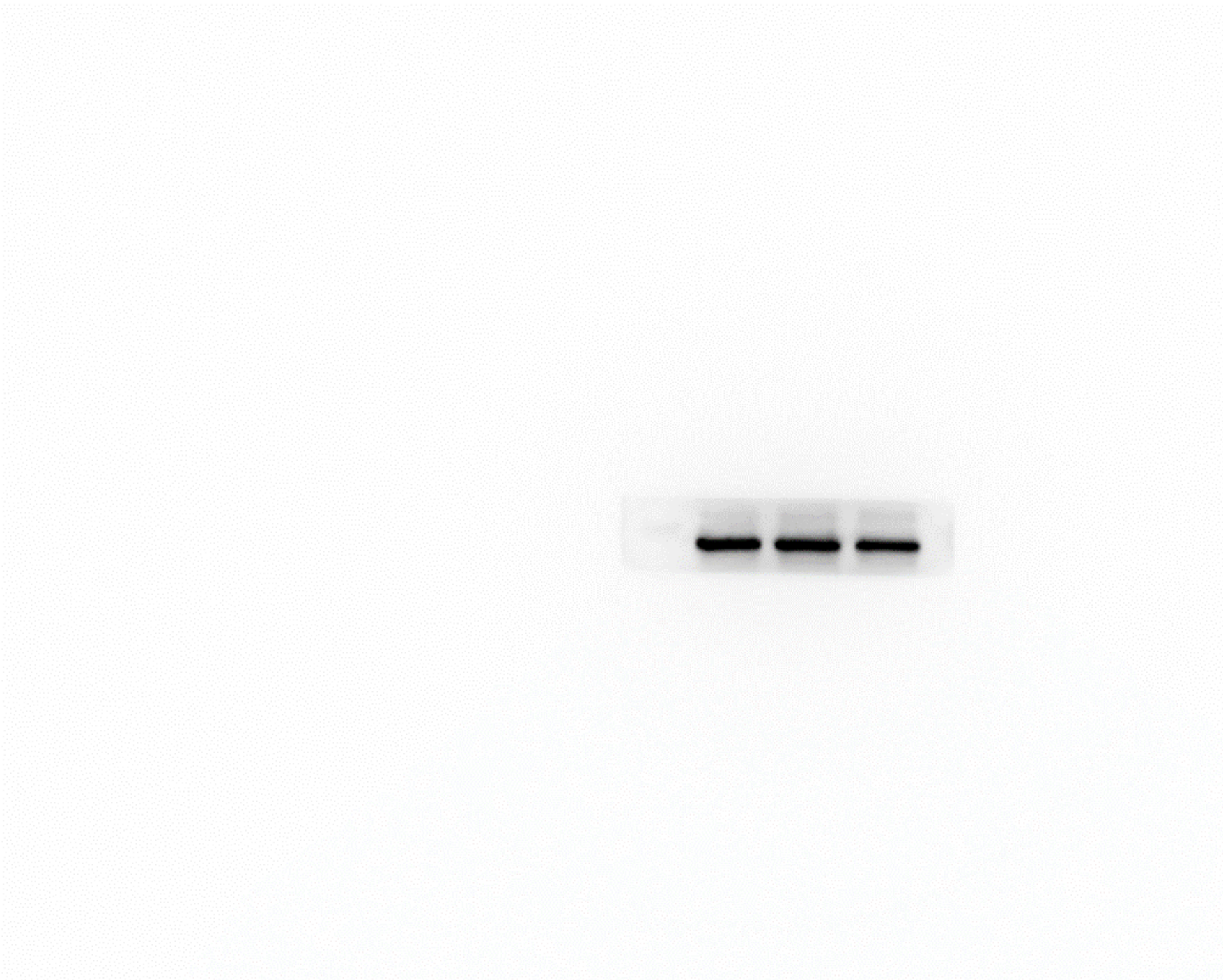

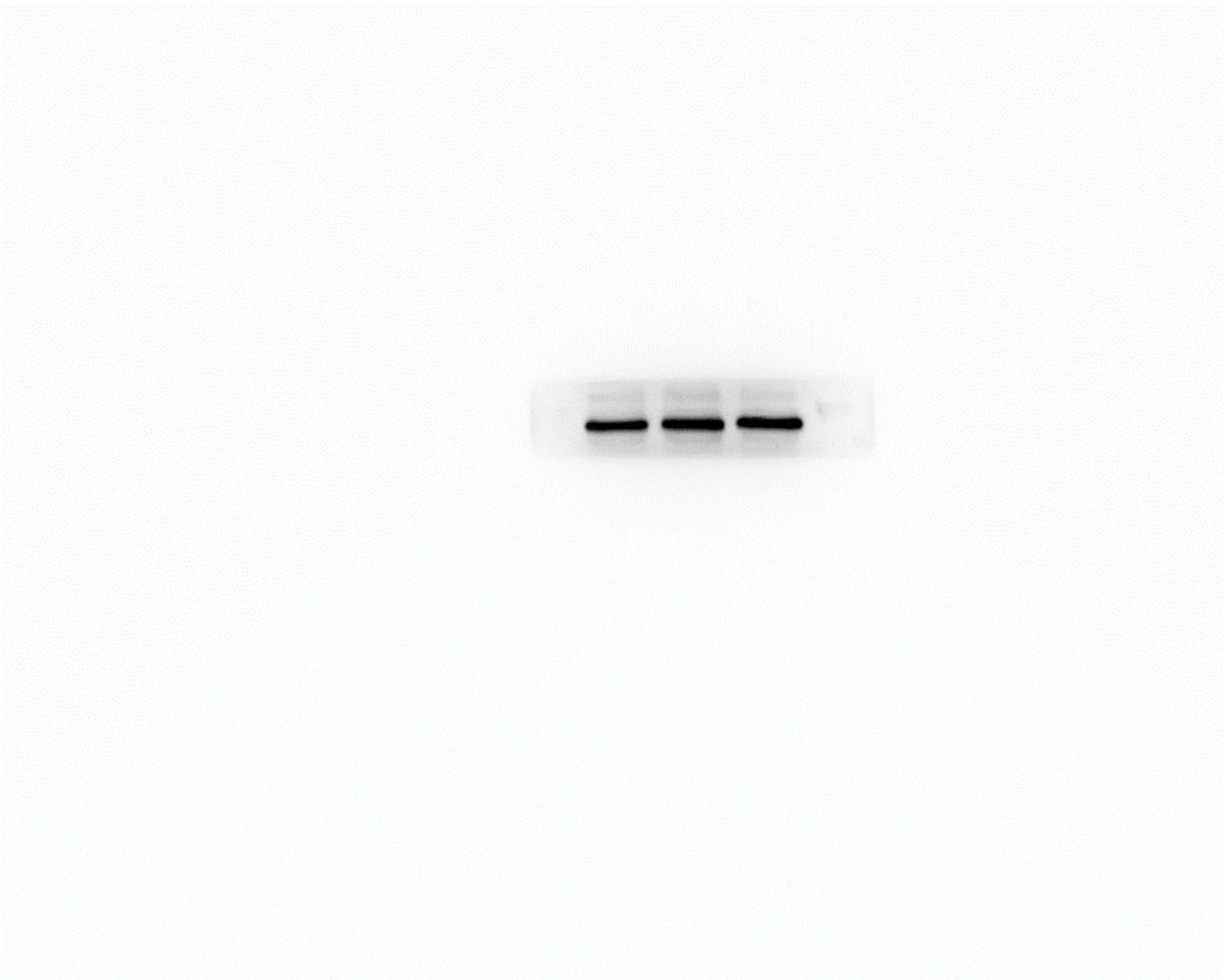

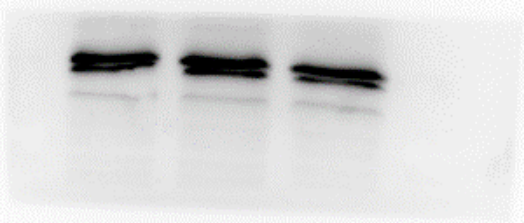

P62 in the cell (CON group， NaCl group， NaCl+CCCP group)

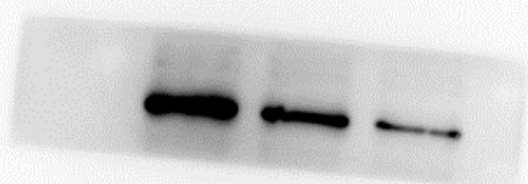

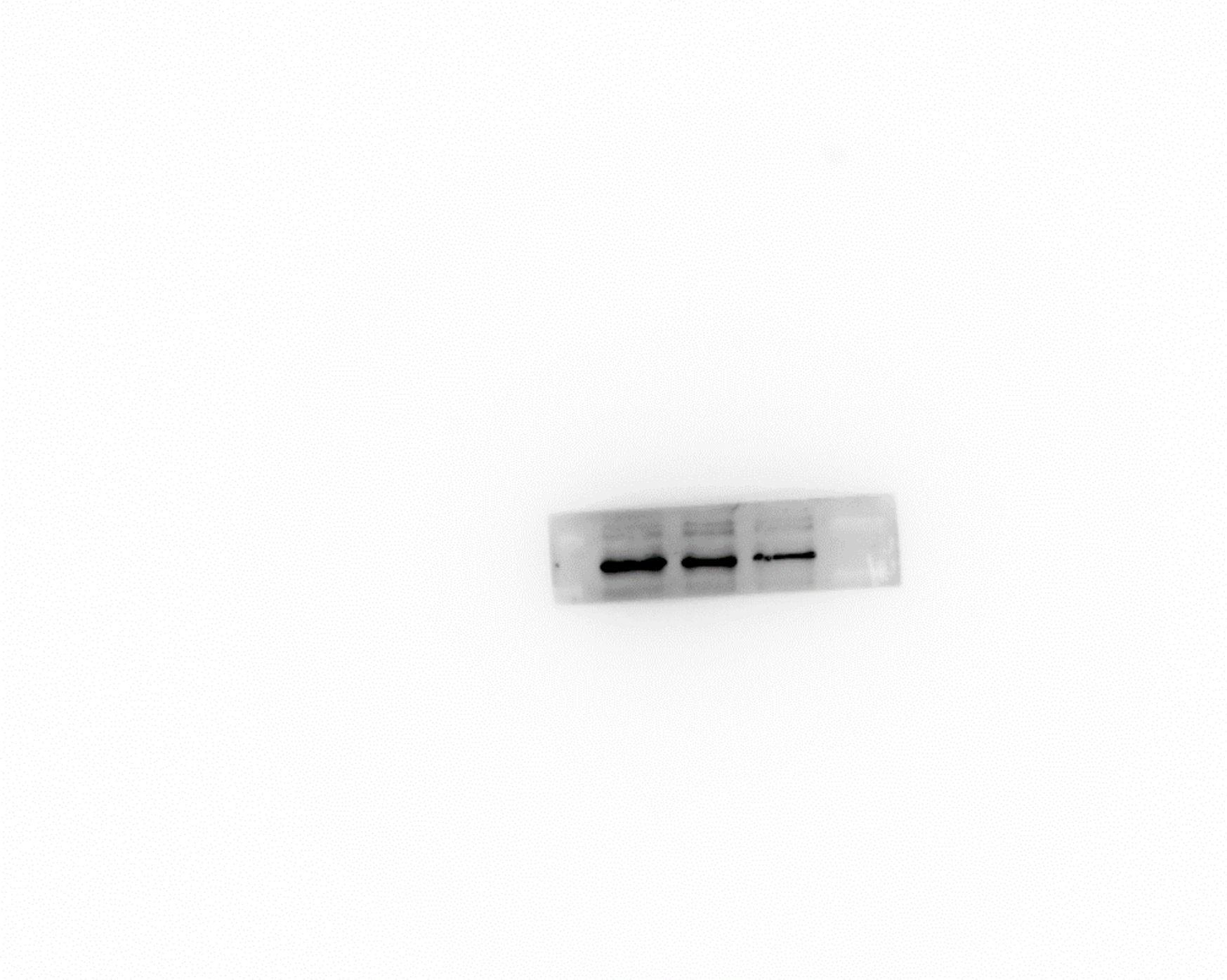

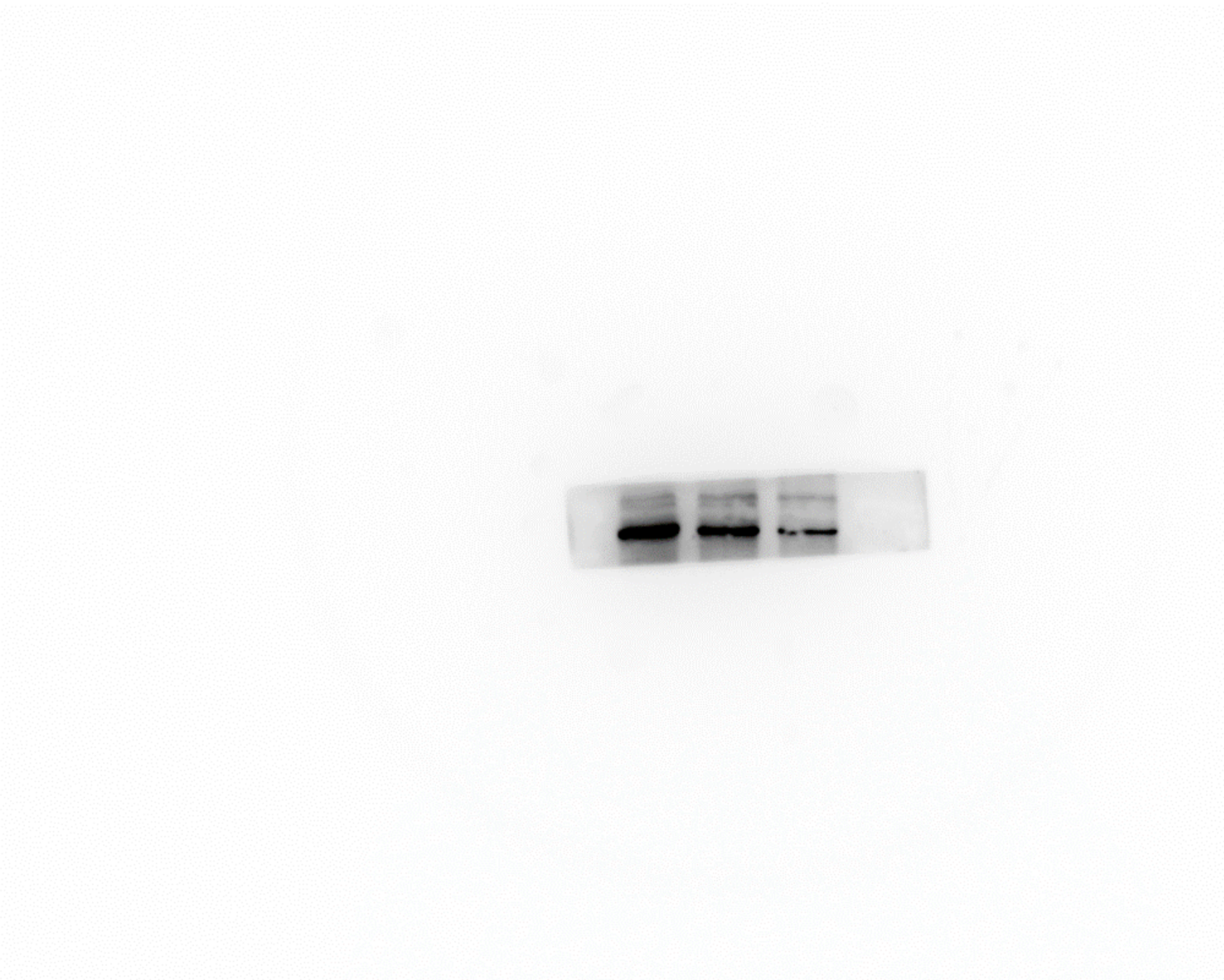

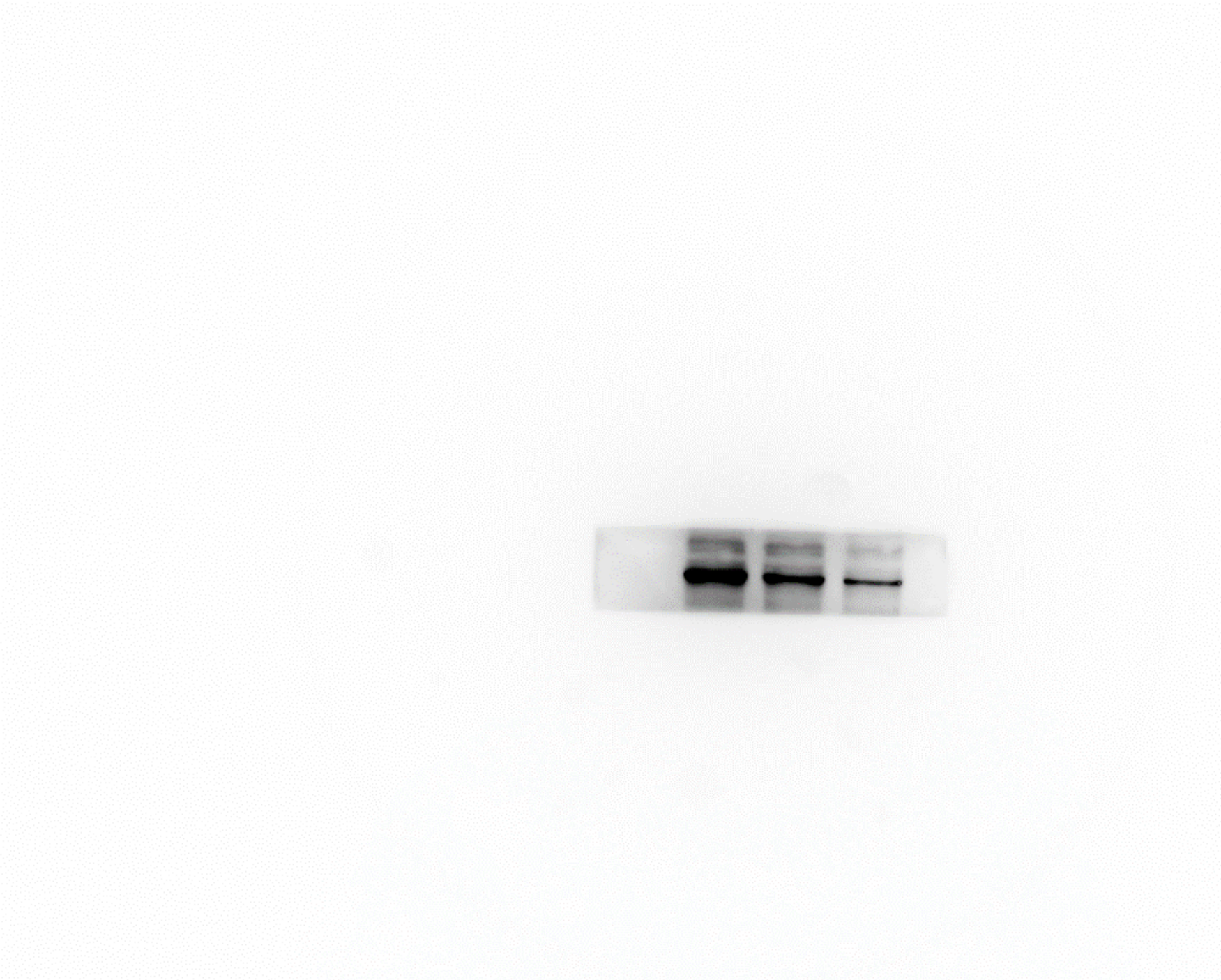

LC3 in the cell (CON group， NaCl group，NaCl+CCCP group)

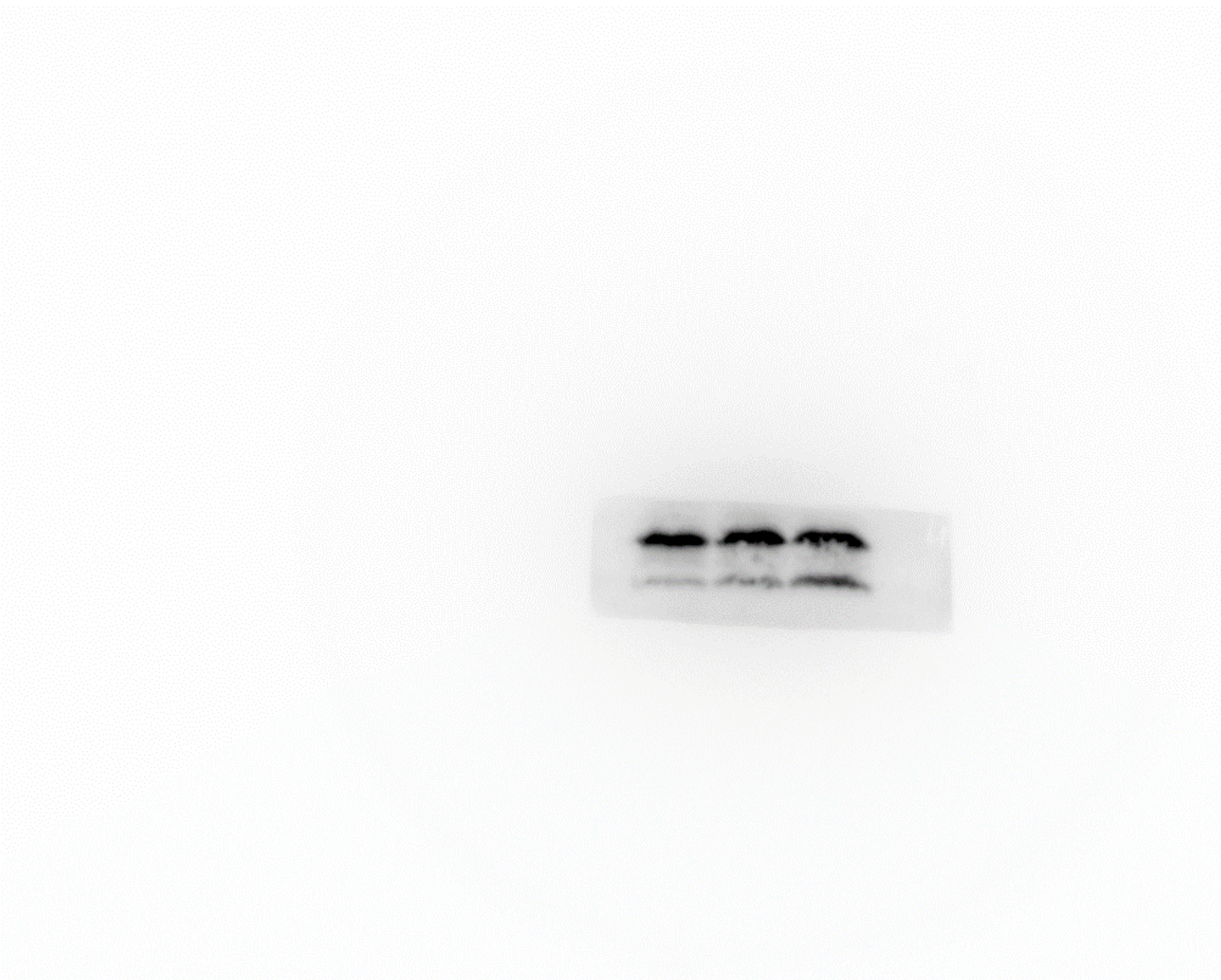

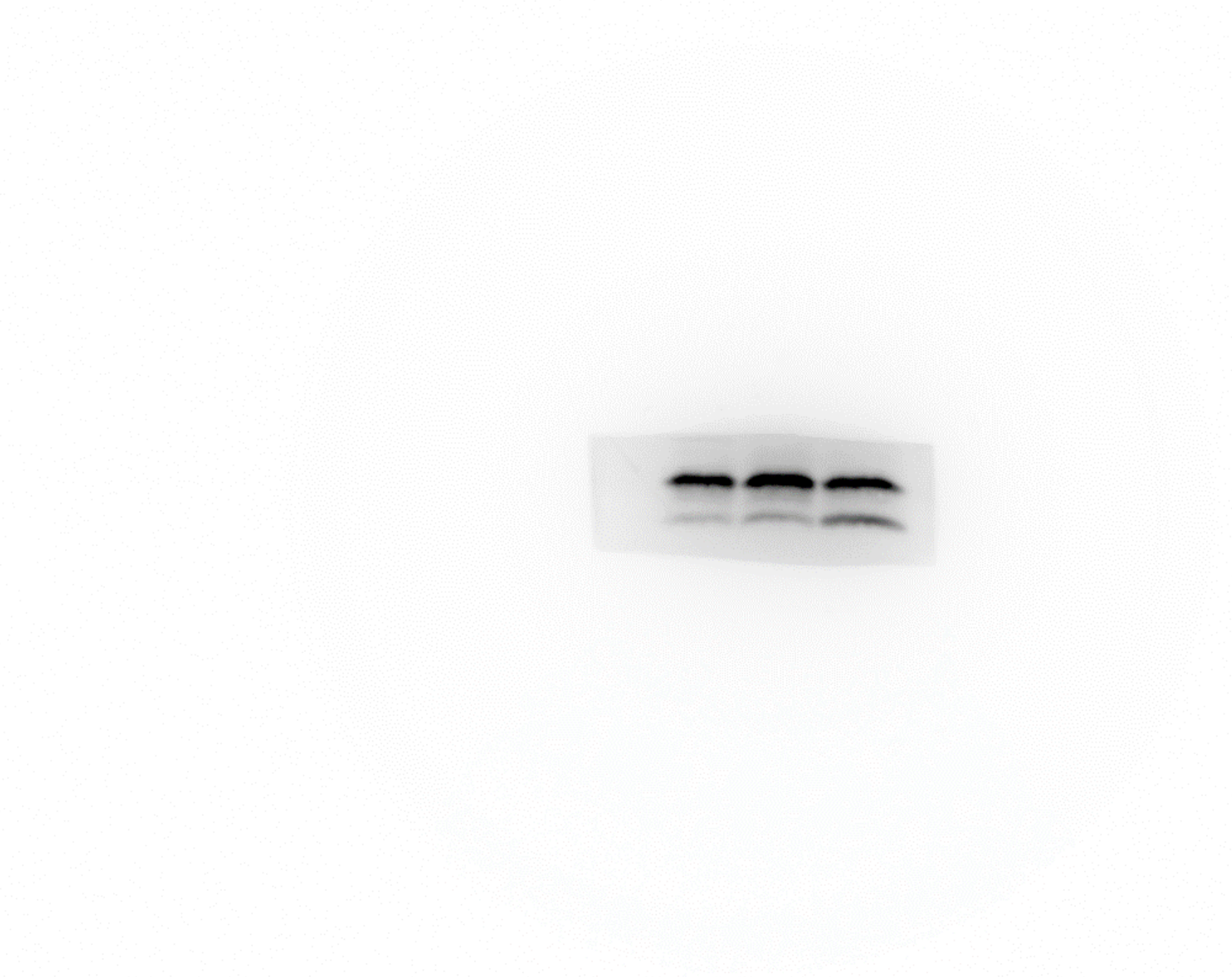

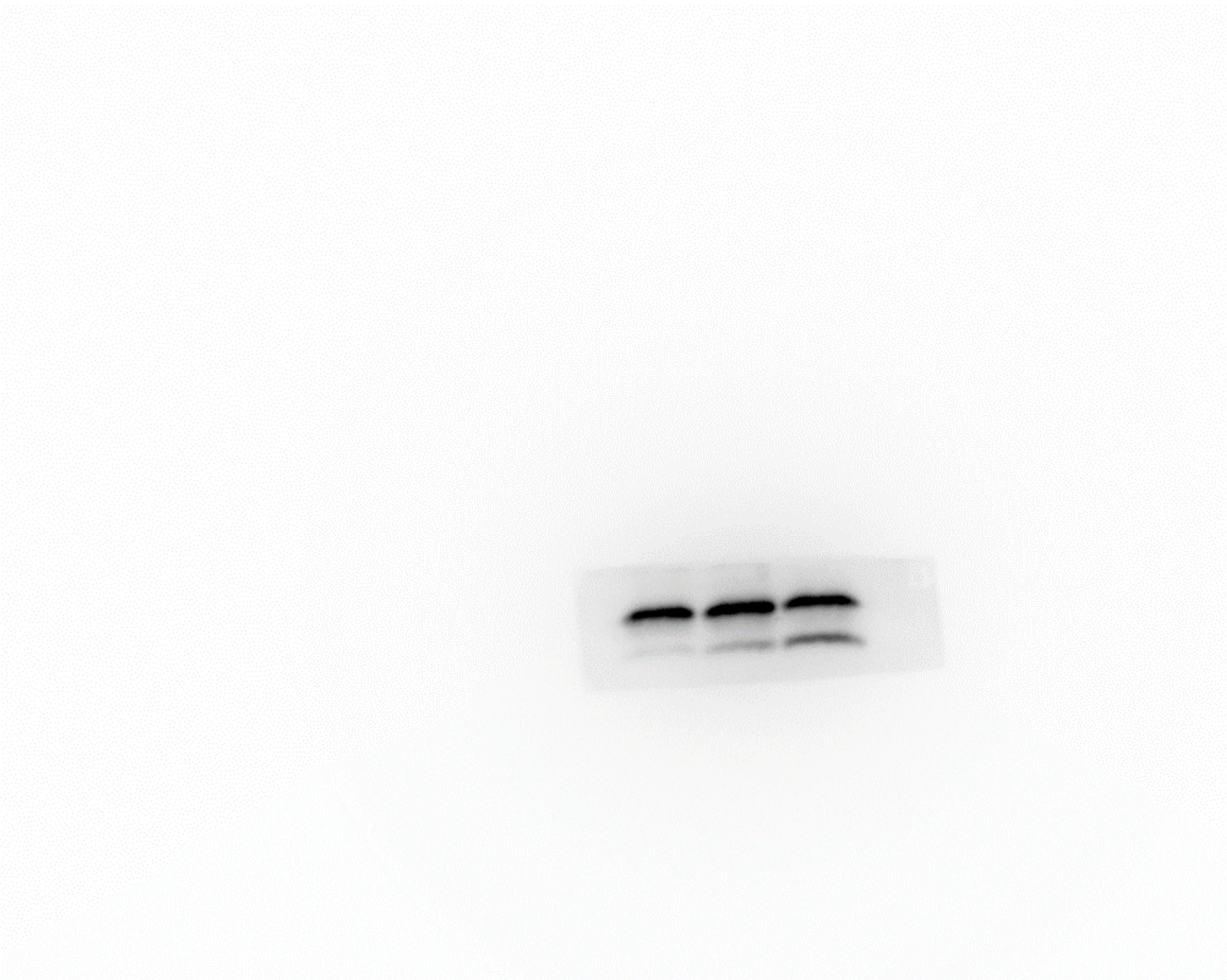

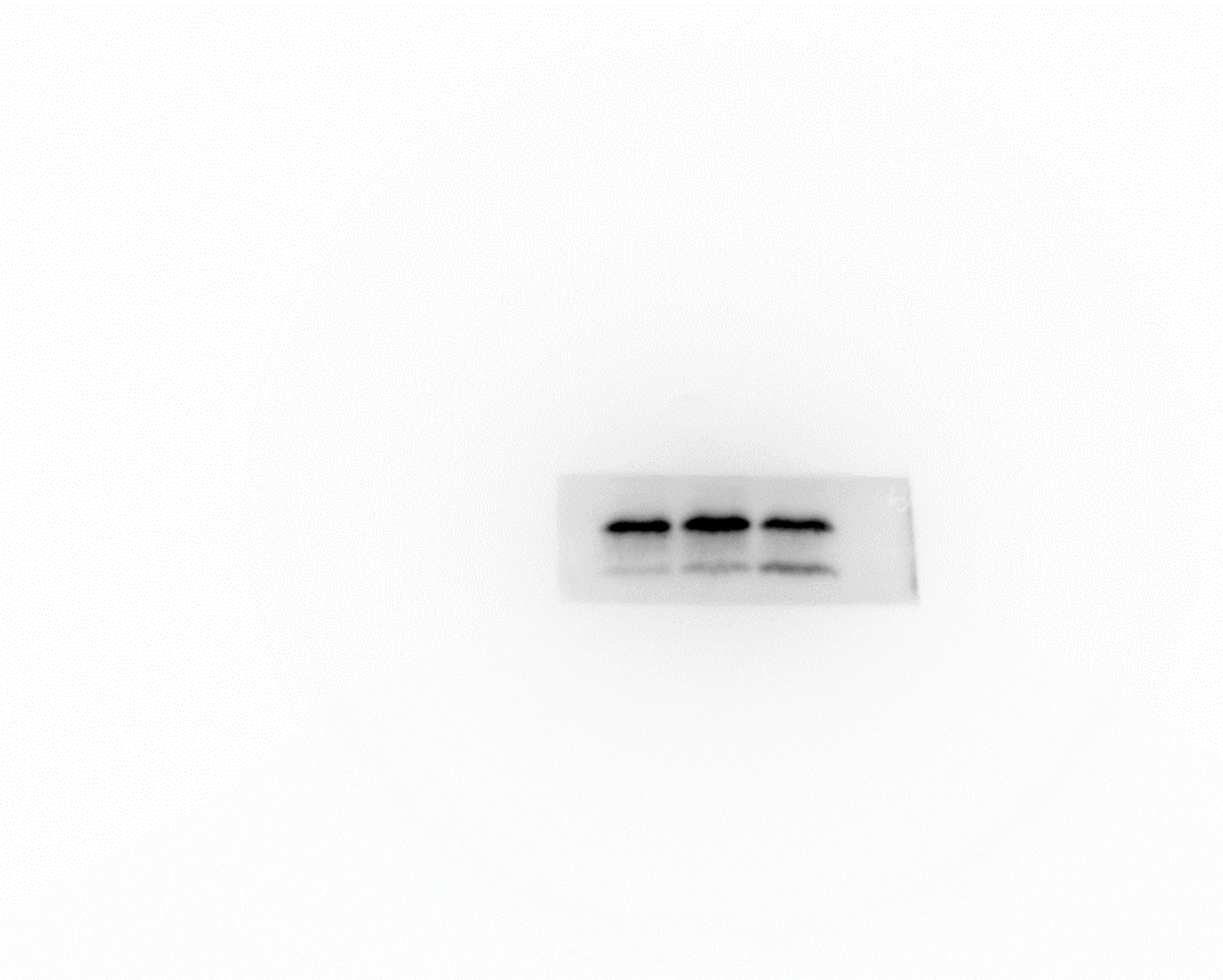

BACE in the cell (CON group， NaCl group， NaCl+CCCP group)

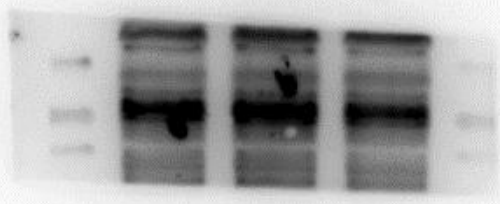

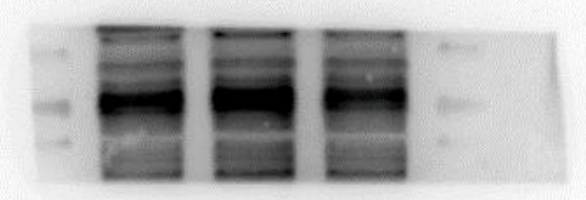

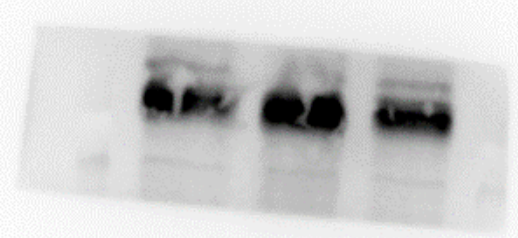

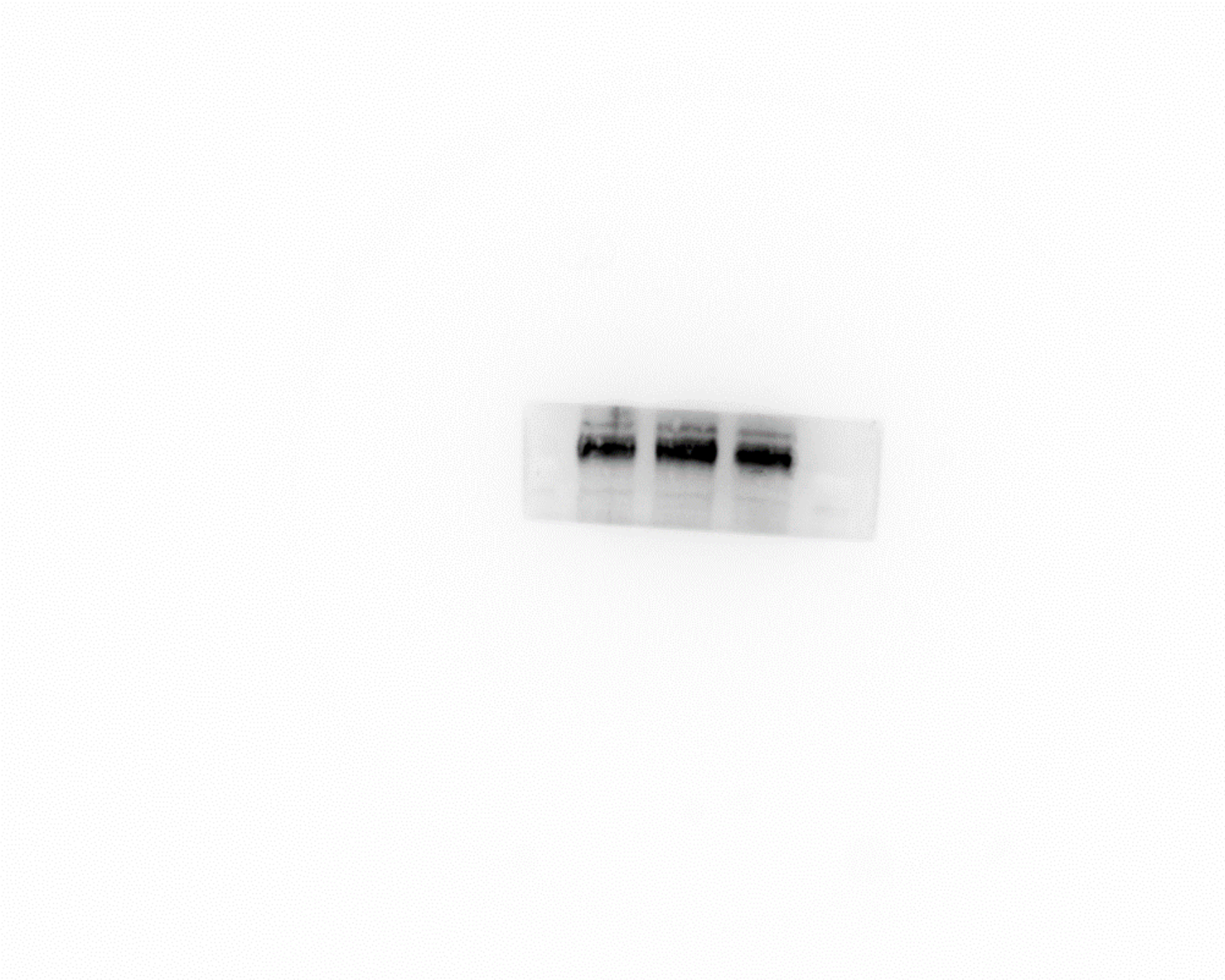

Presenlin1 in the cell (CON group， NaCl group，NaCl+CCCP group)

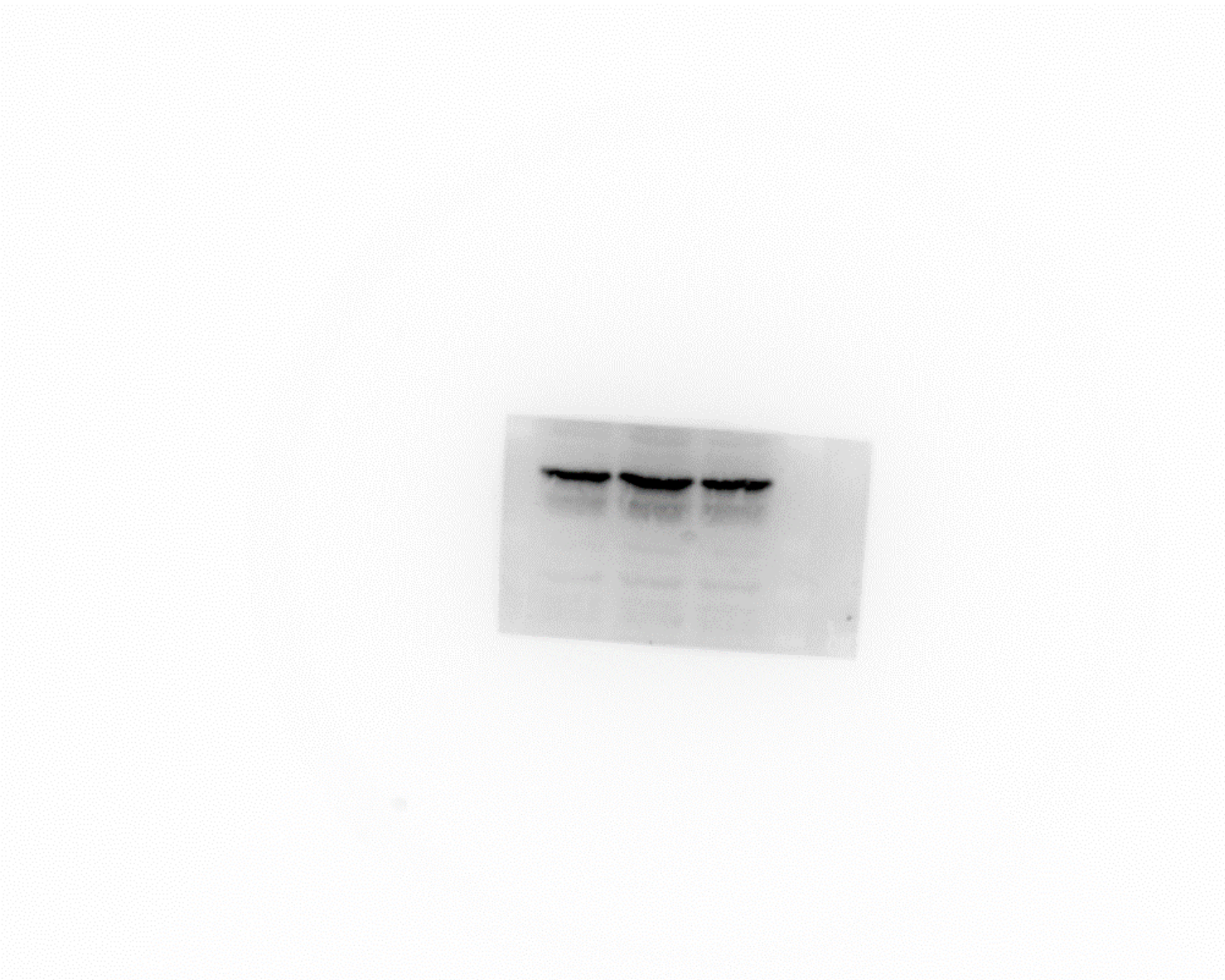

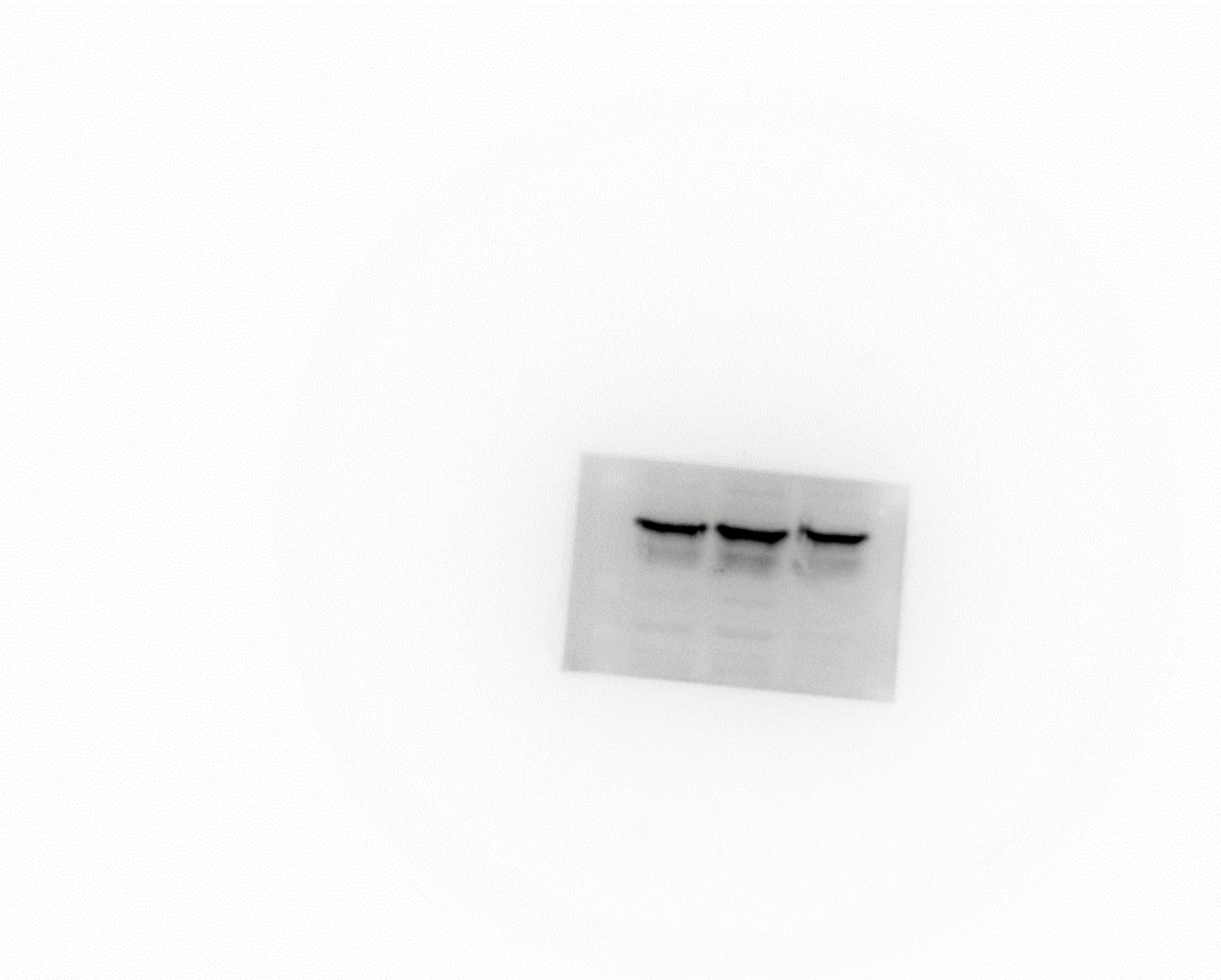

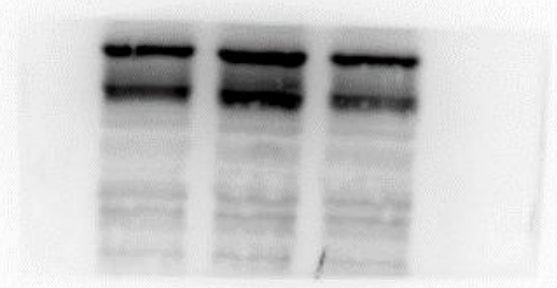

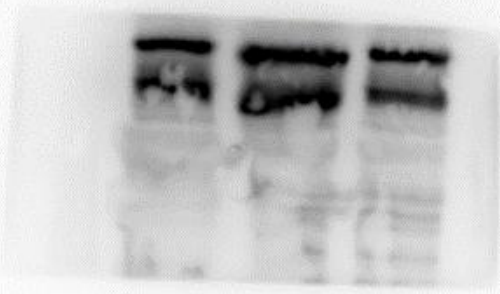

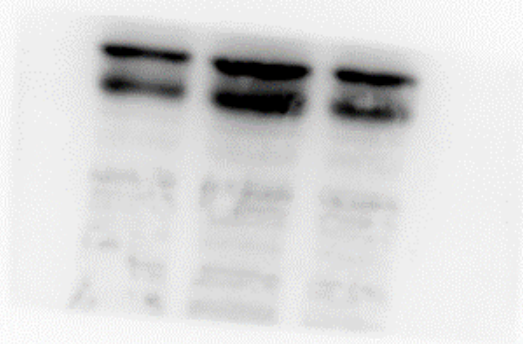

Presenlin2 in the cell (CON group， NaCl group，NaCl+CCCP group)

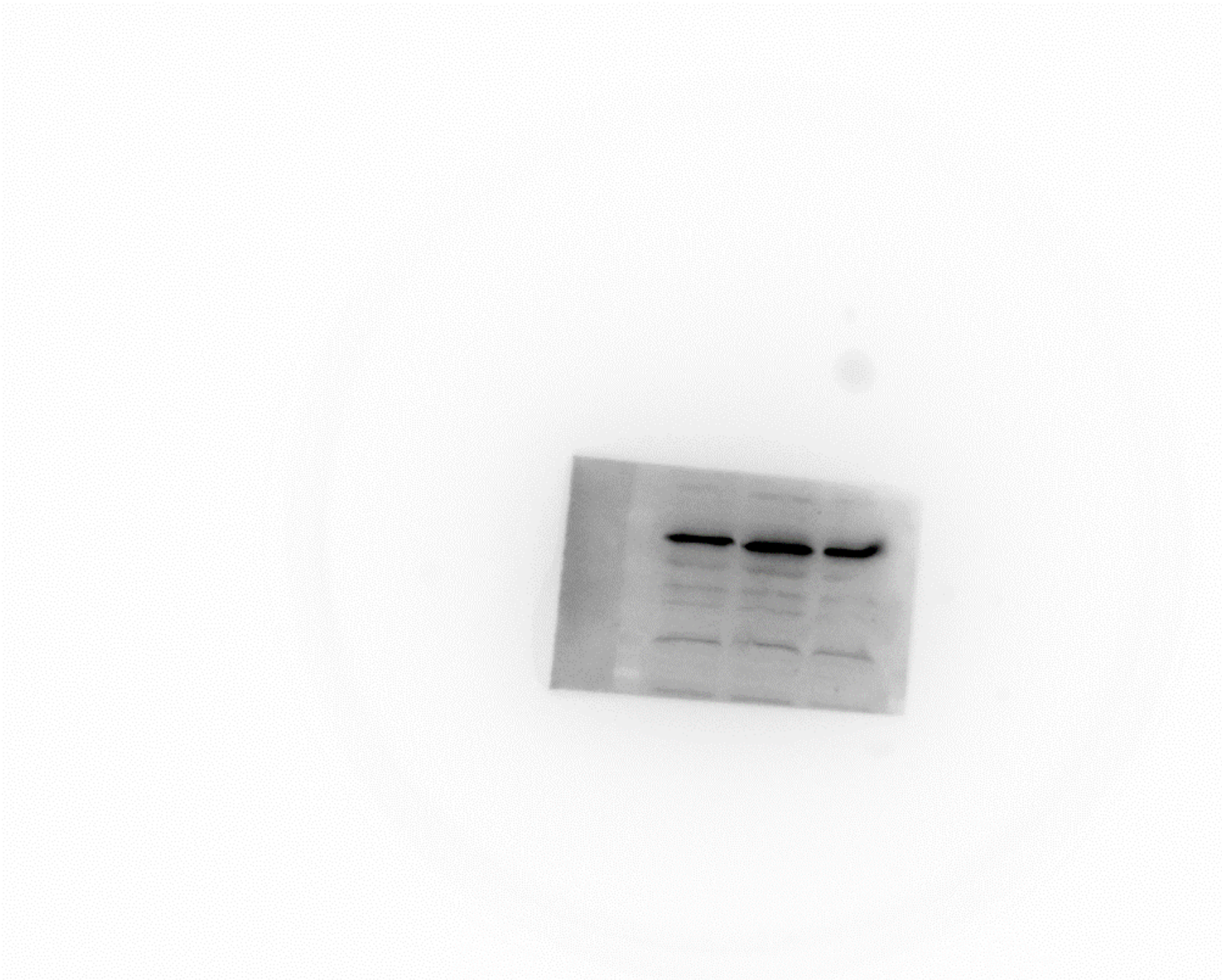

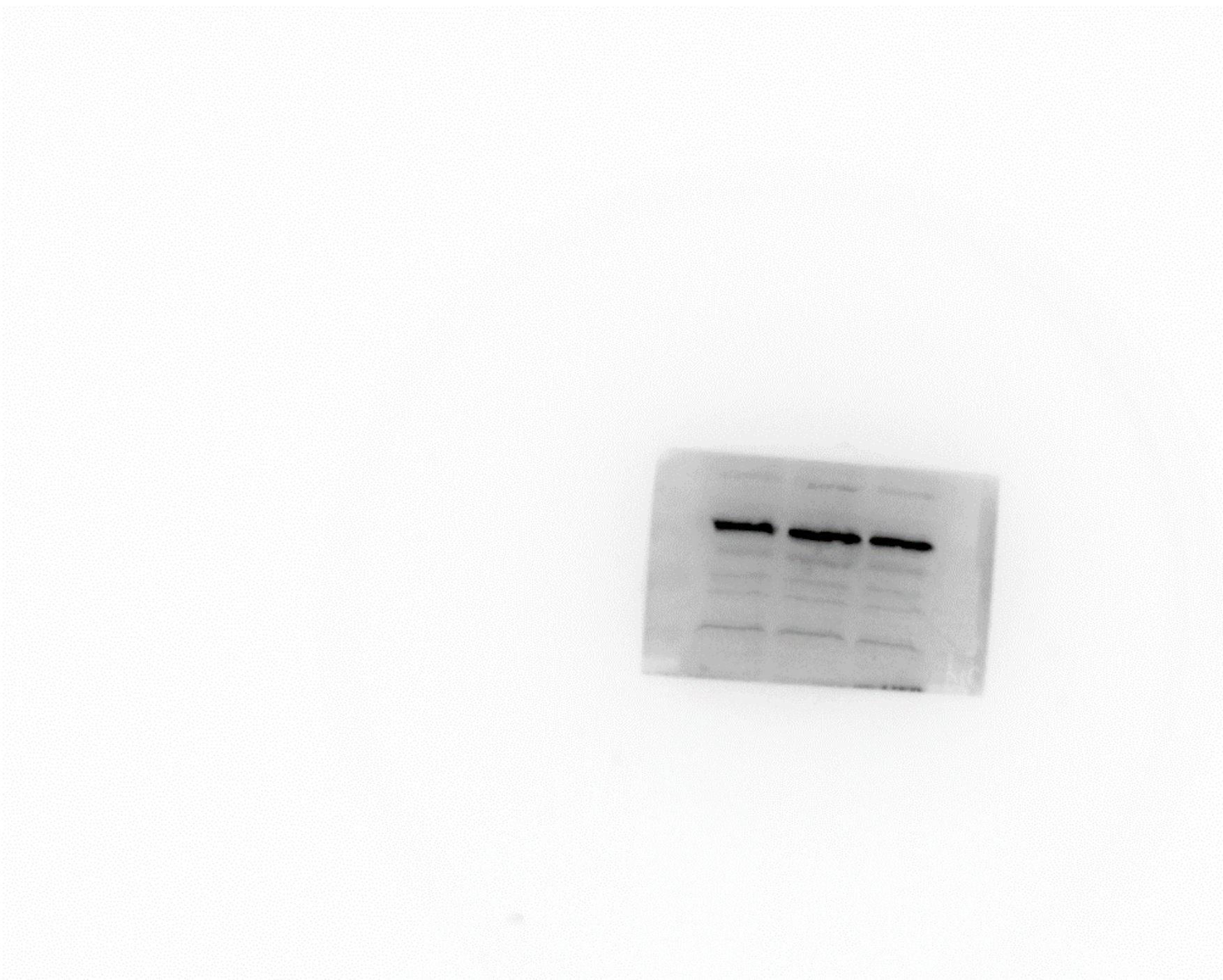

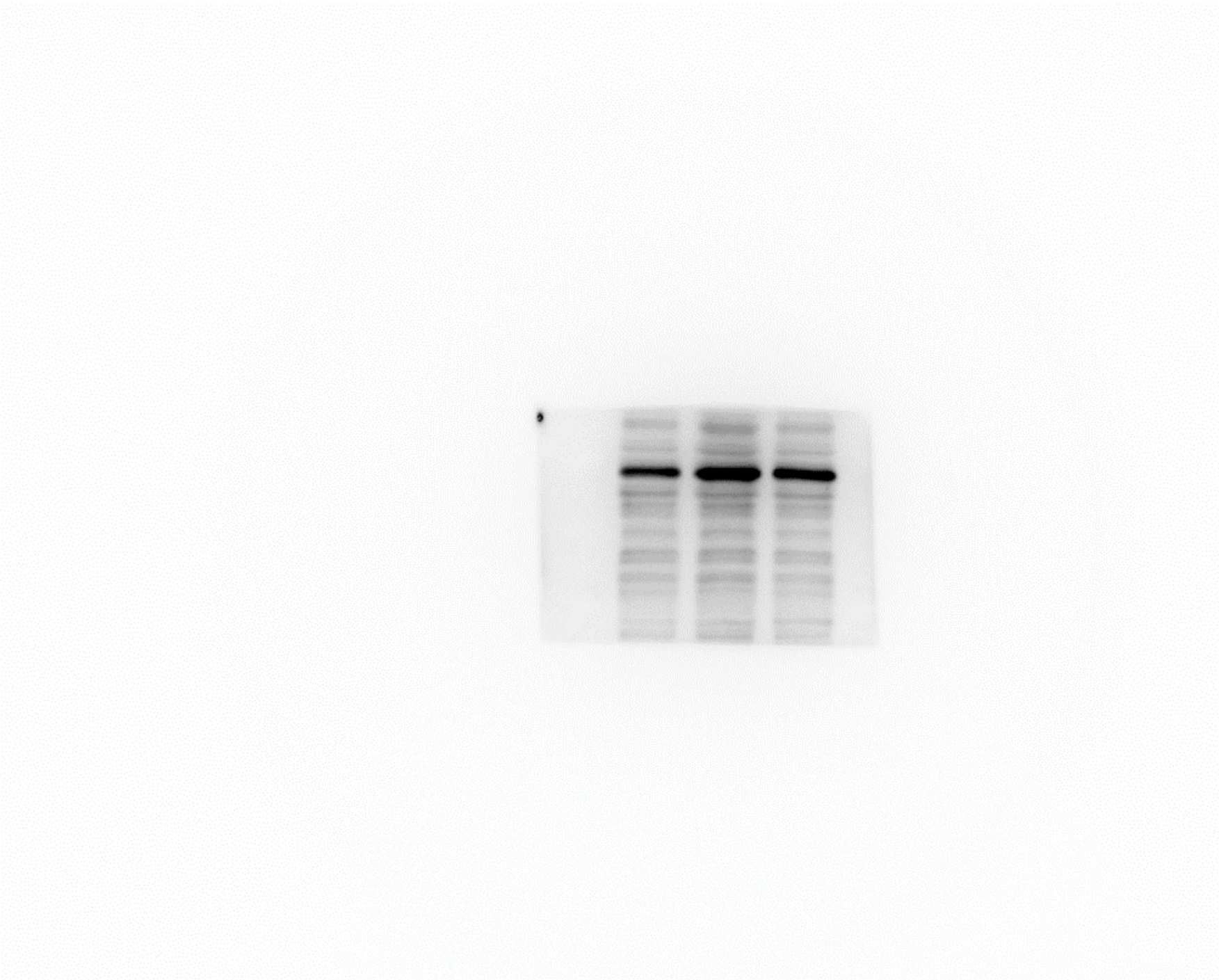

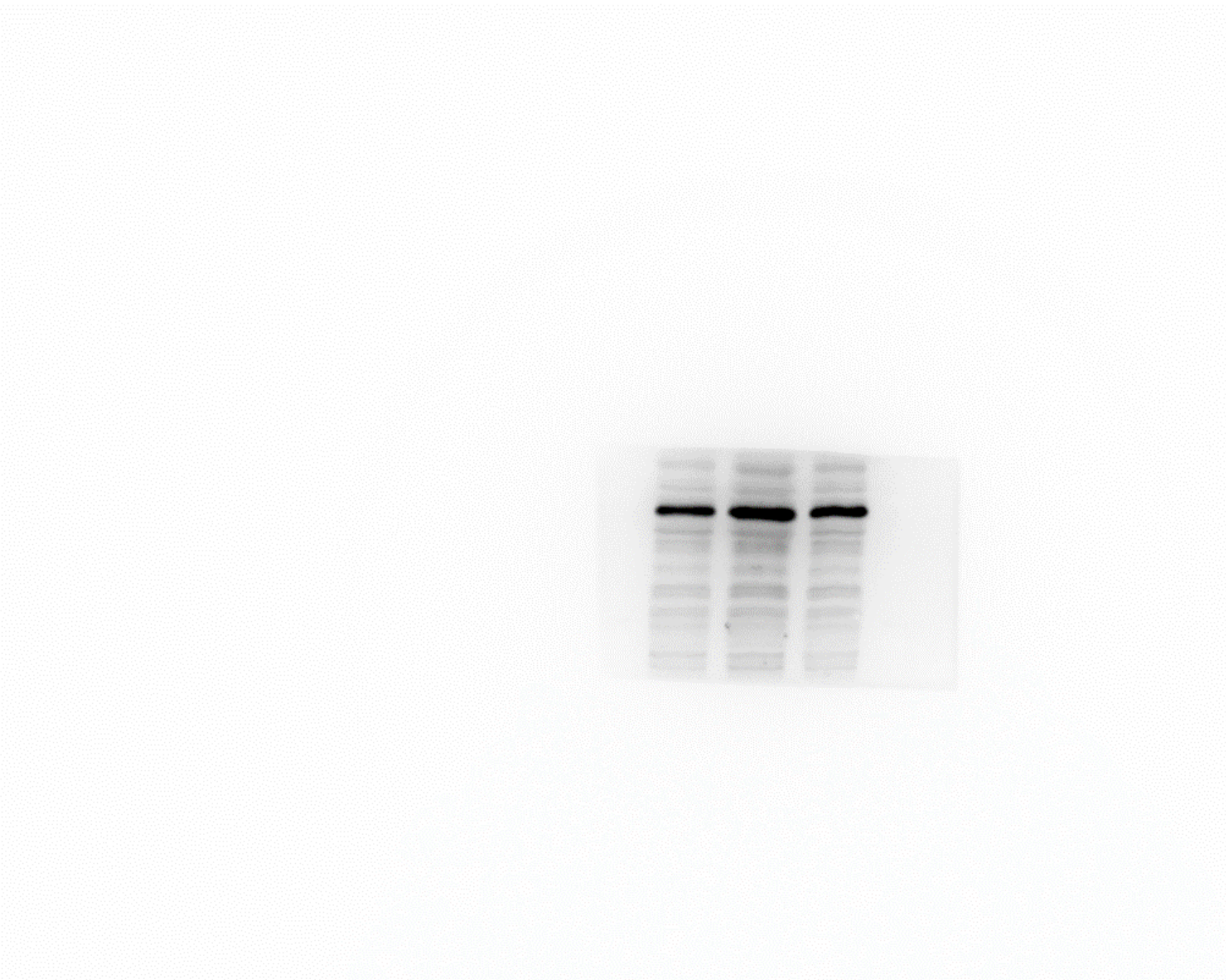

Neprilysin in the cell (CON group， NaCl group，NaCl+CCCP group)

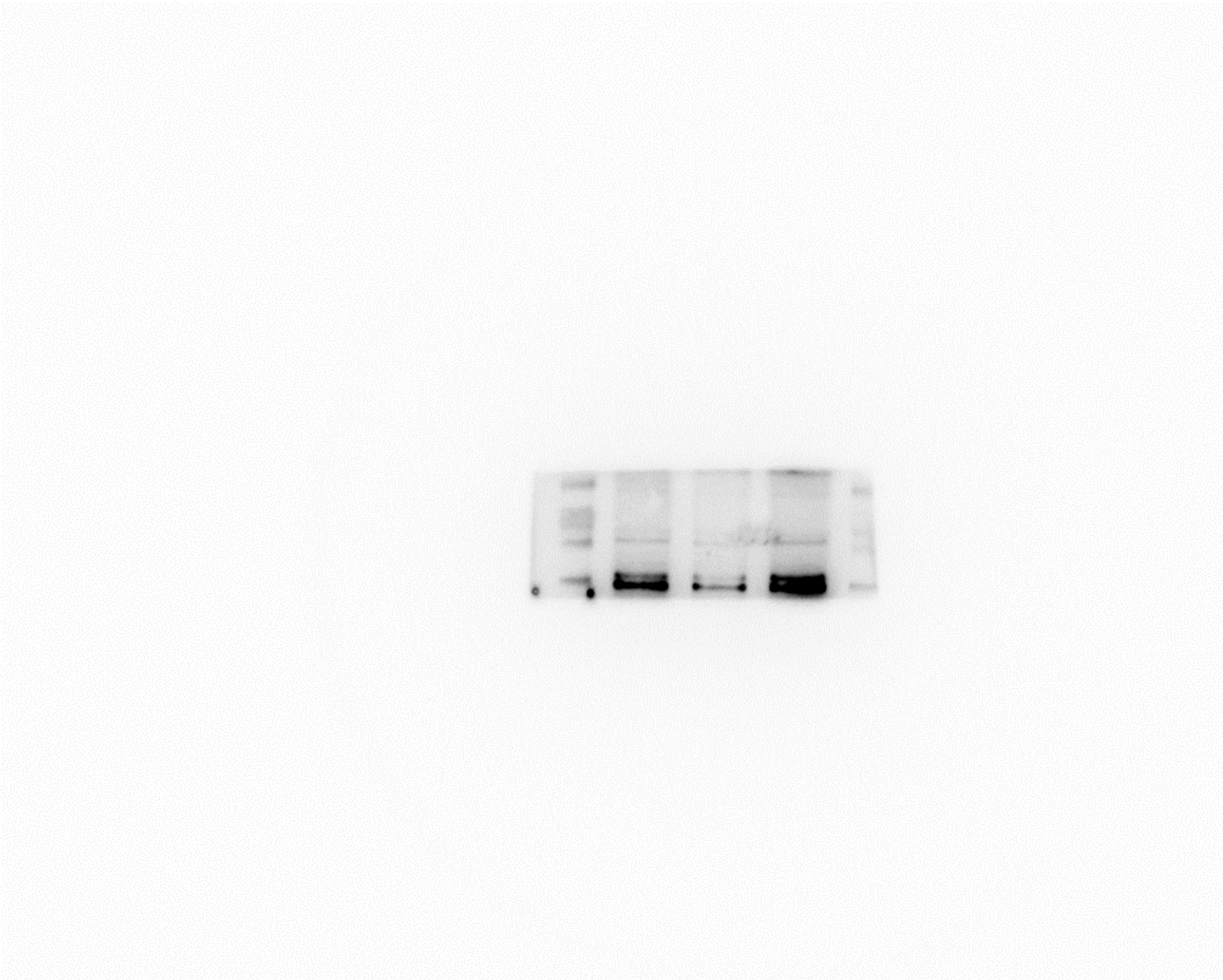

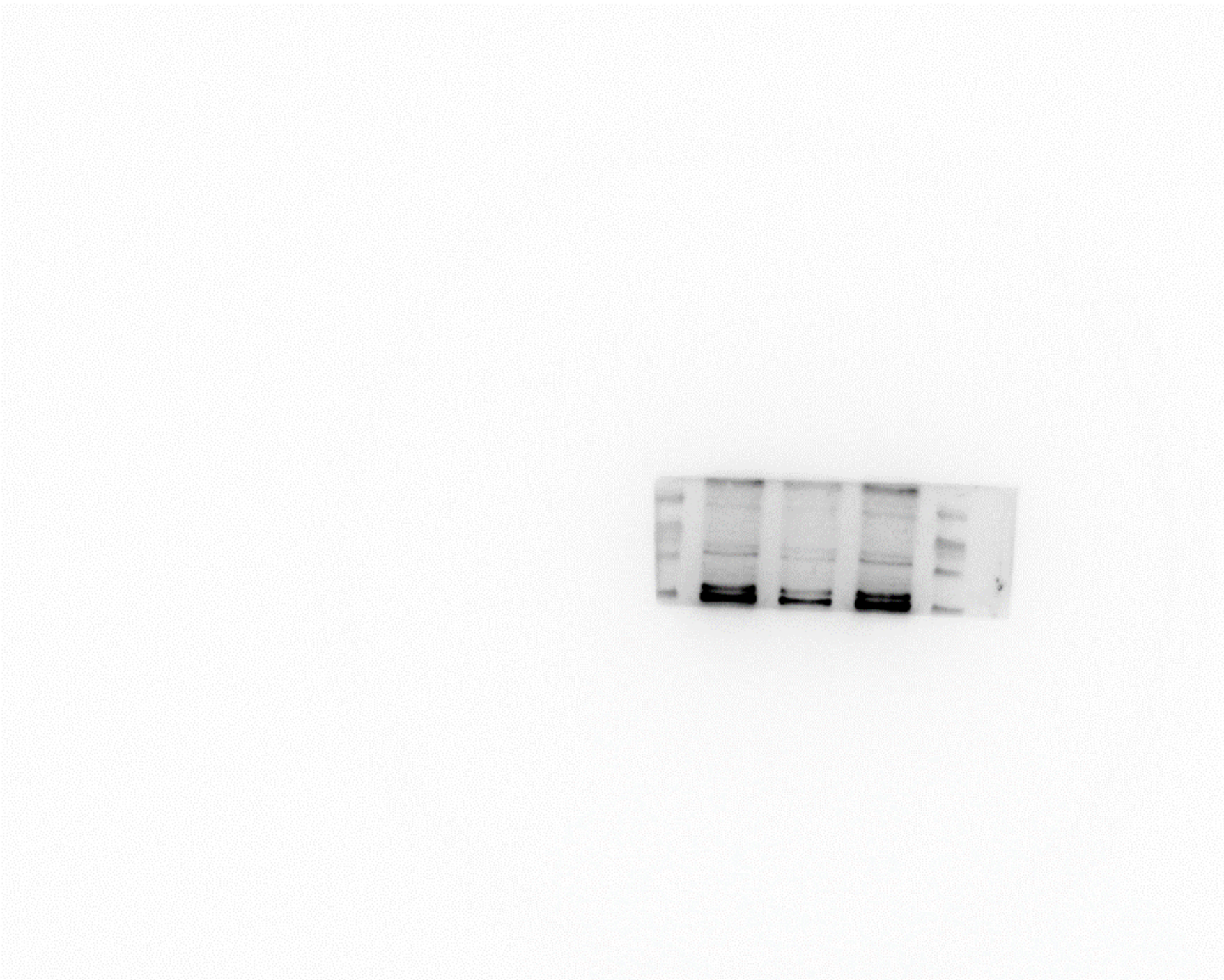

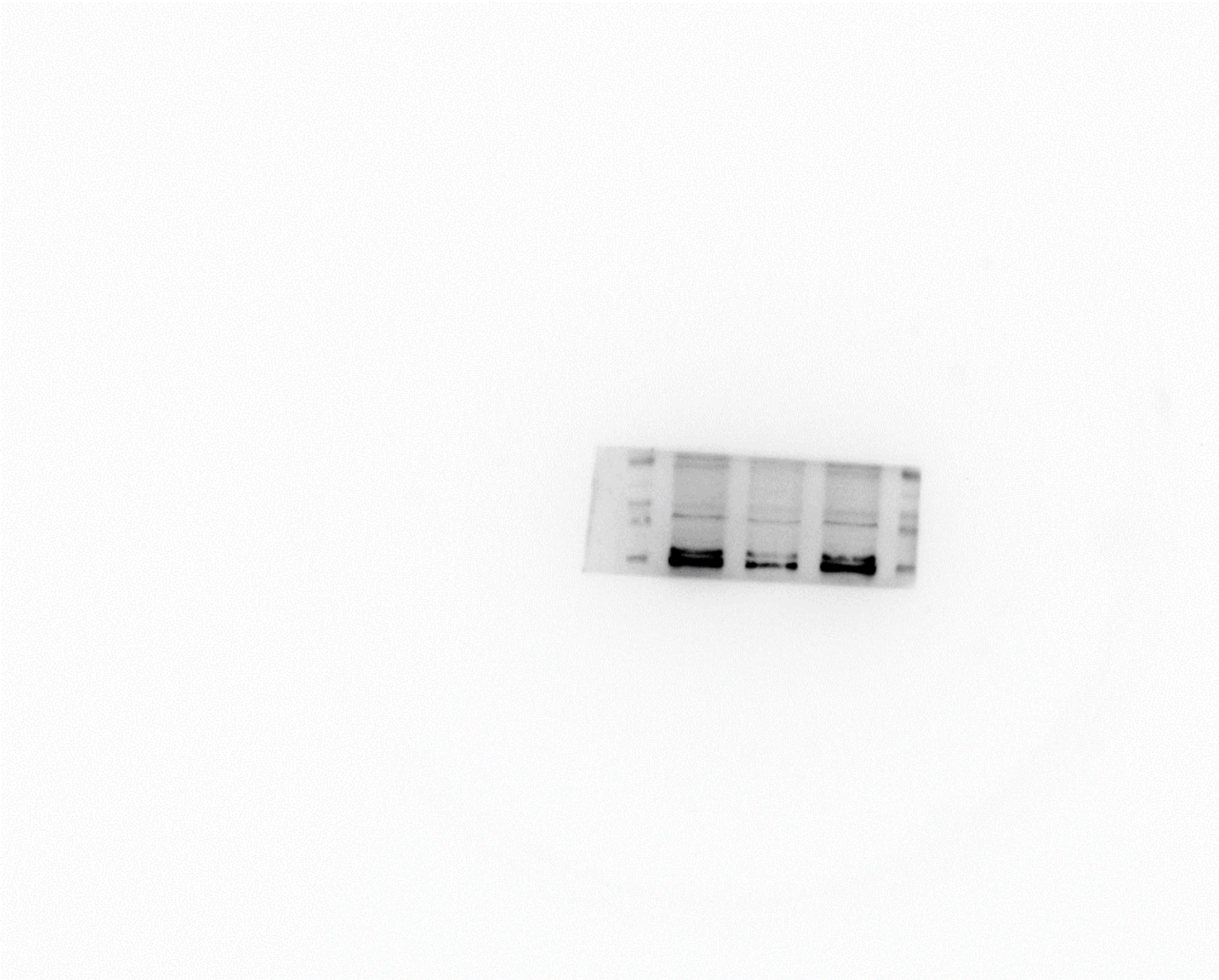

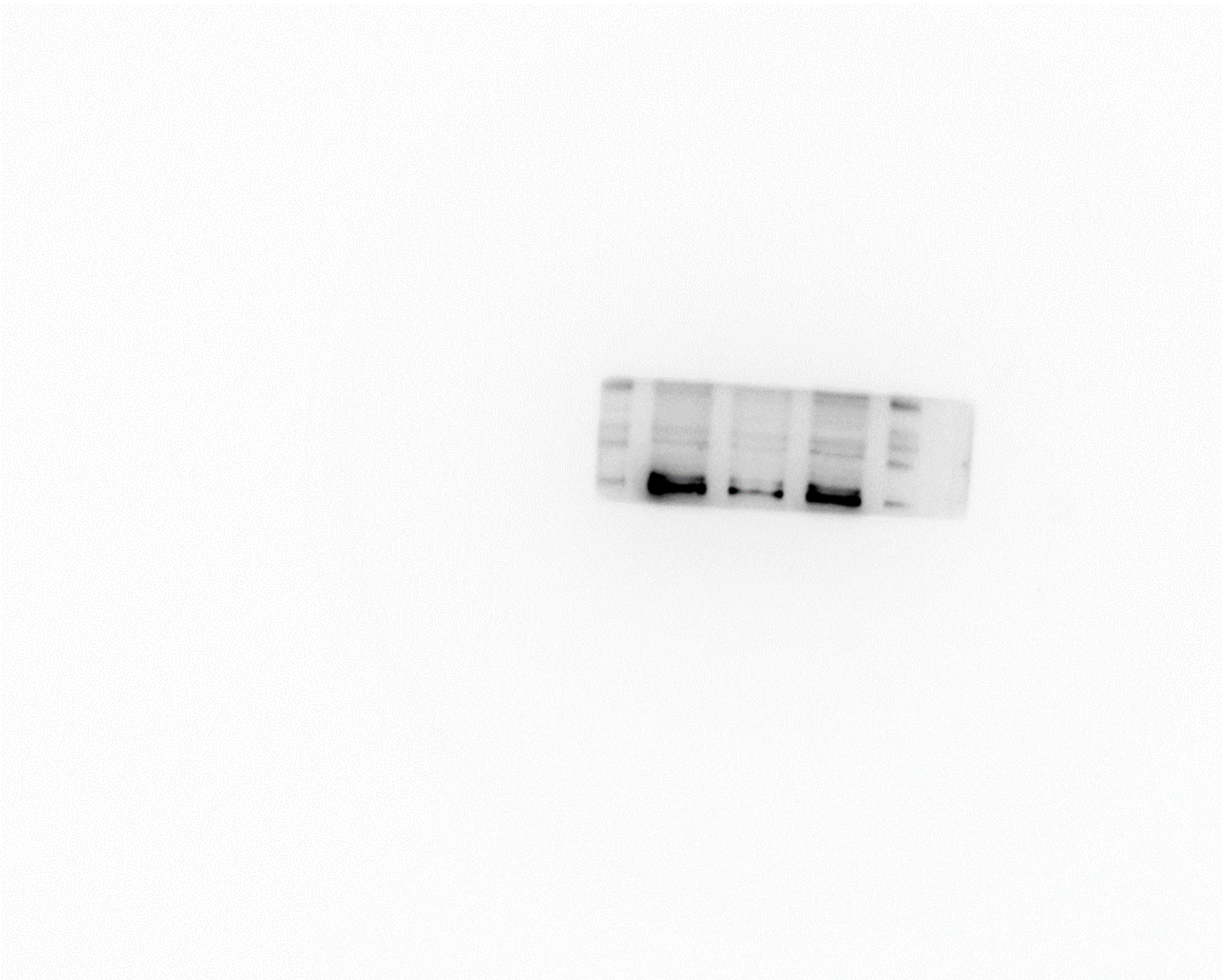

Aβ in the cell (CON group, NaCl group, NaCl+CCCP group)

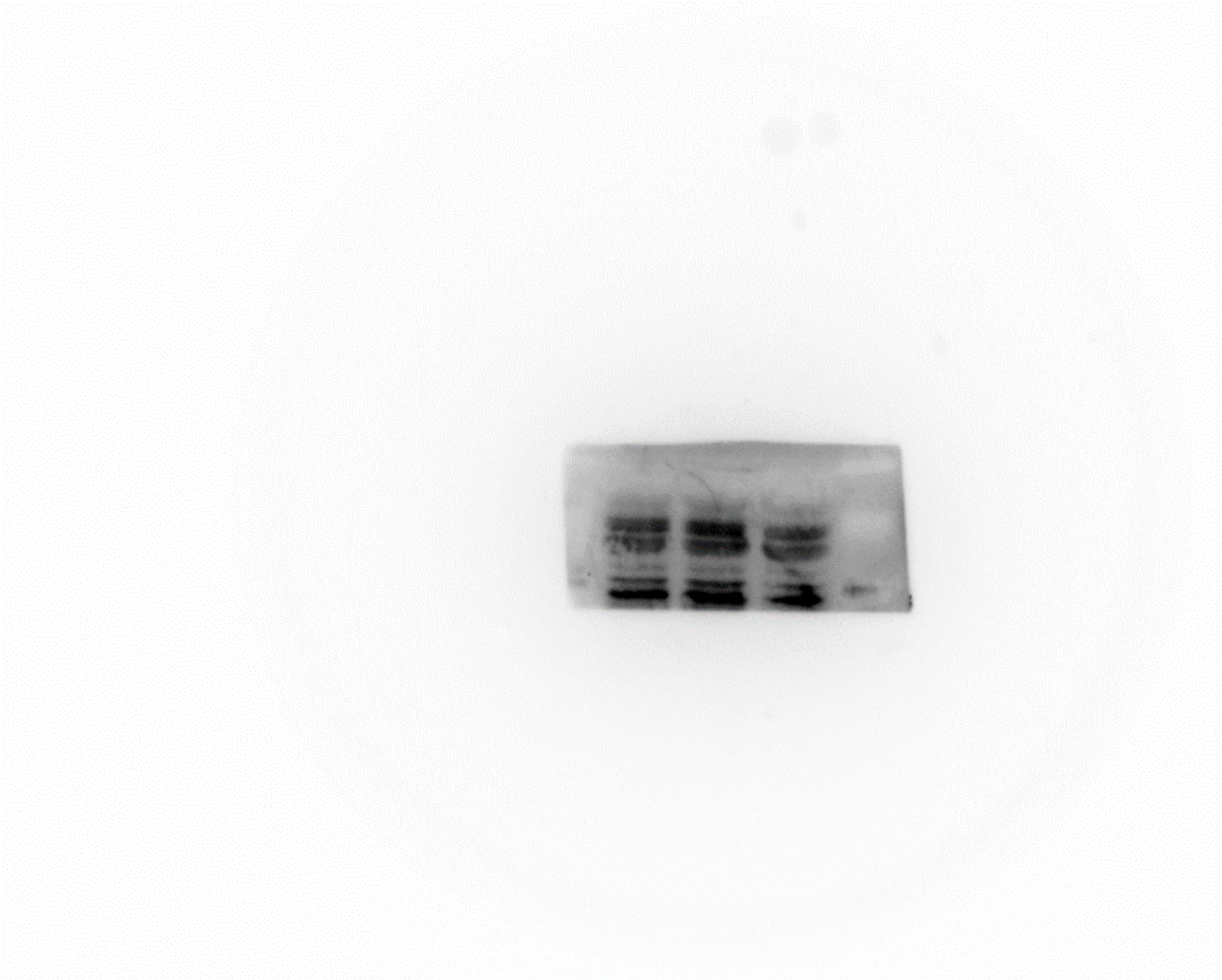

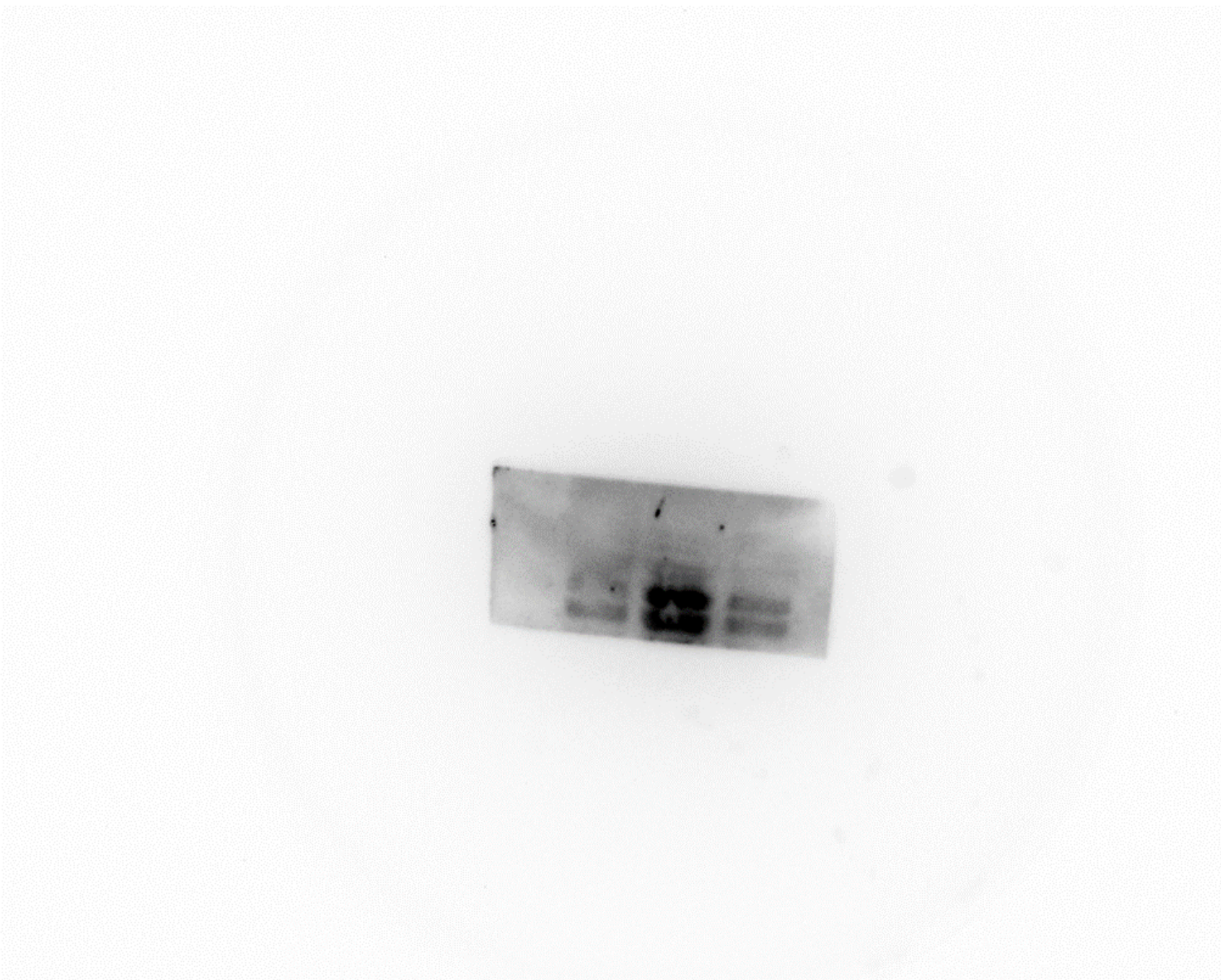

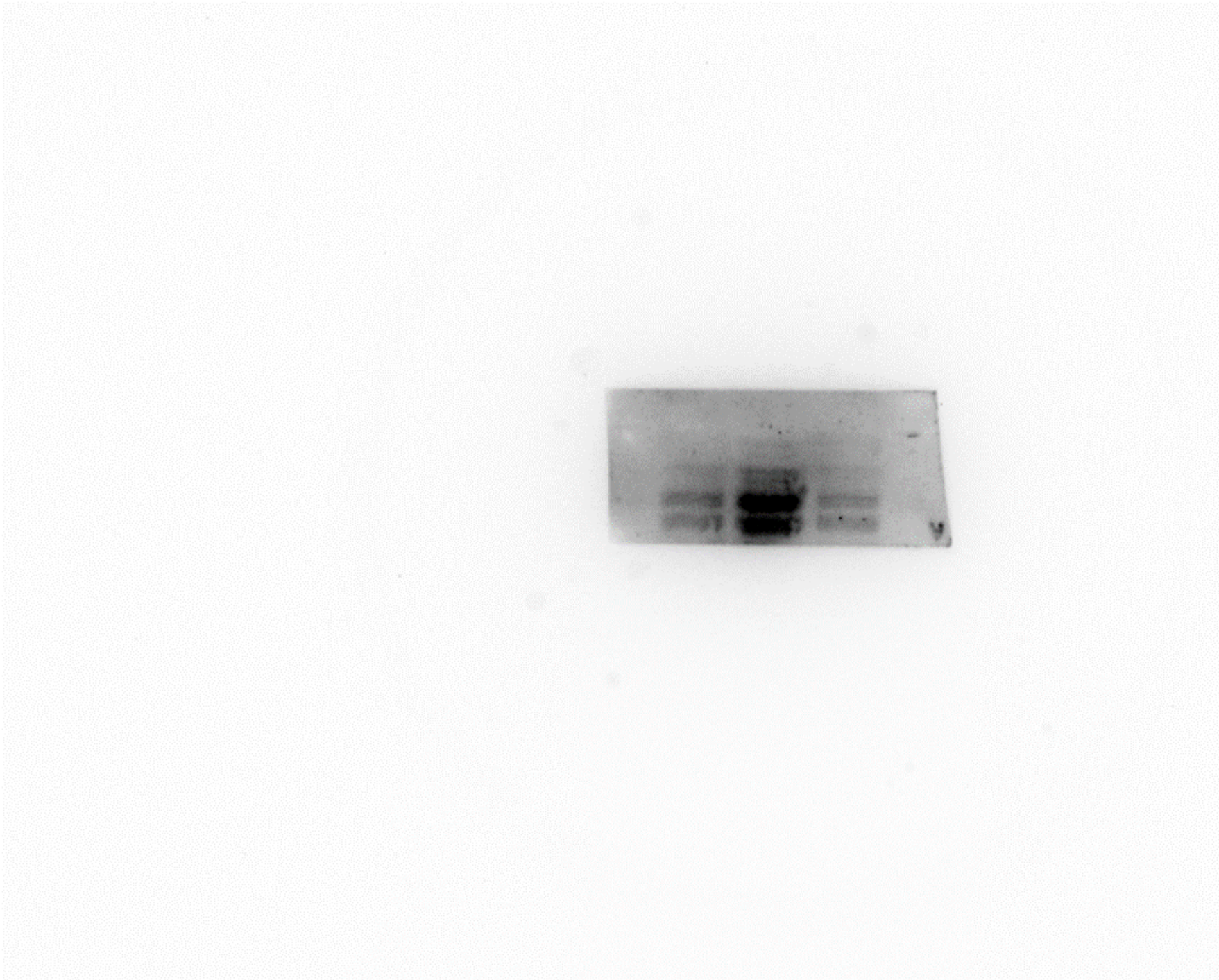

Aβ (1-42) in the cell (CON group, NaCl group, NaCl+CCCP group)

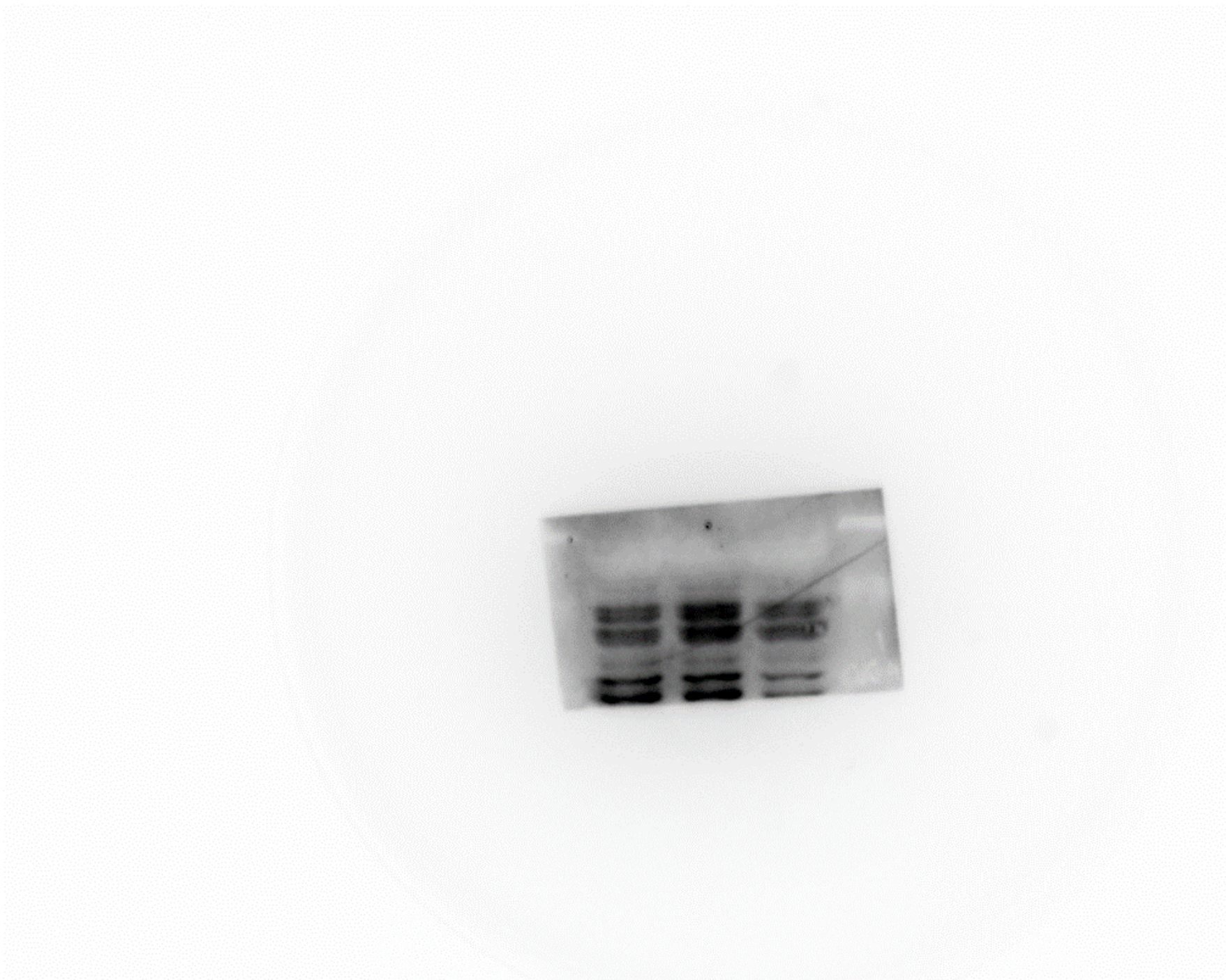

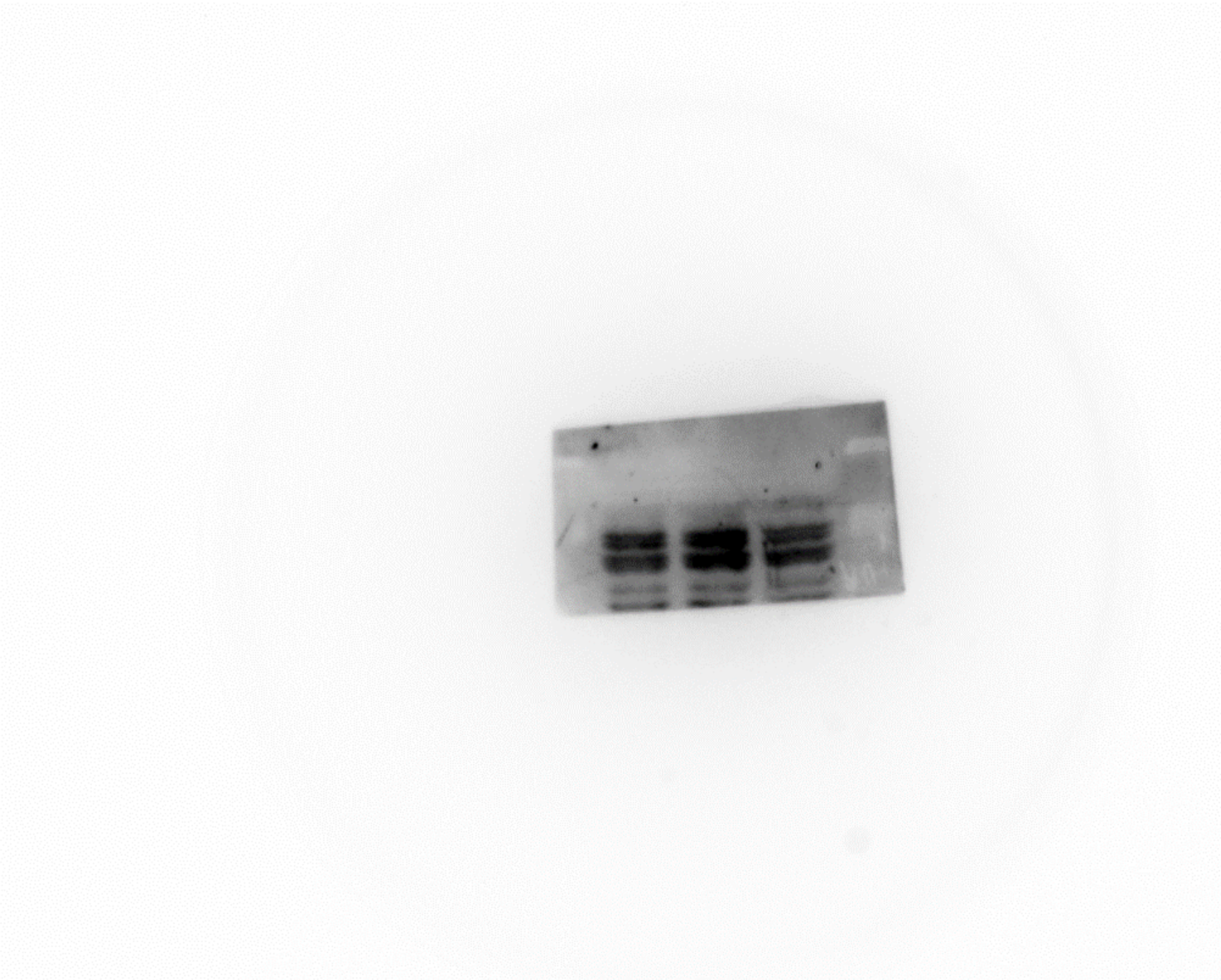

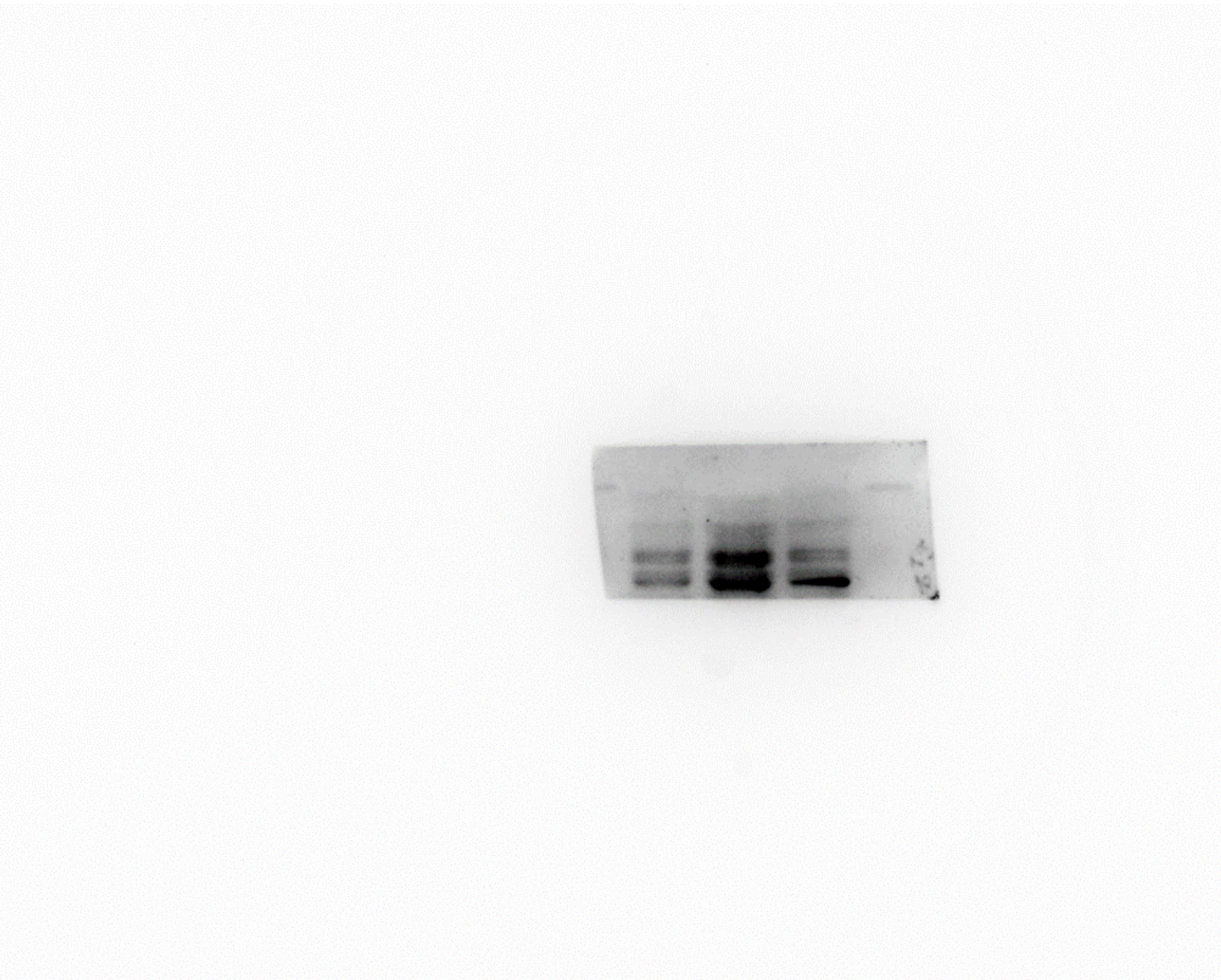

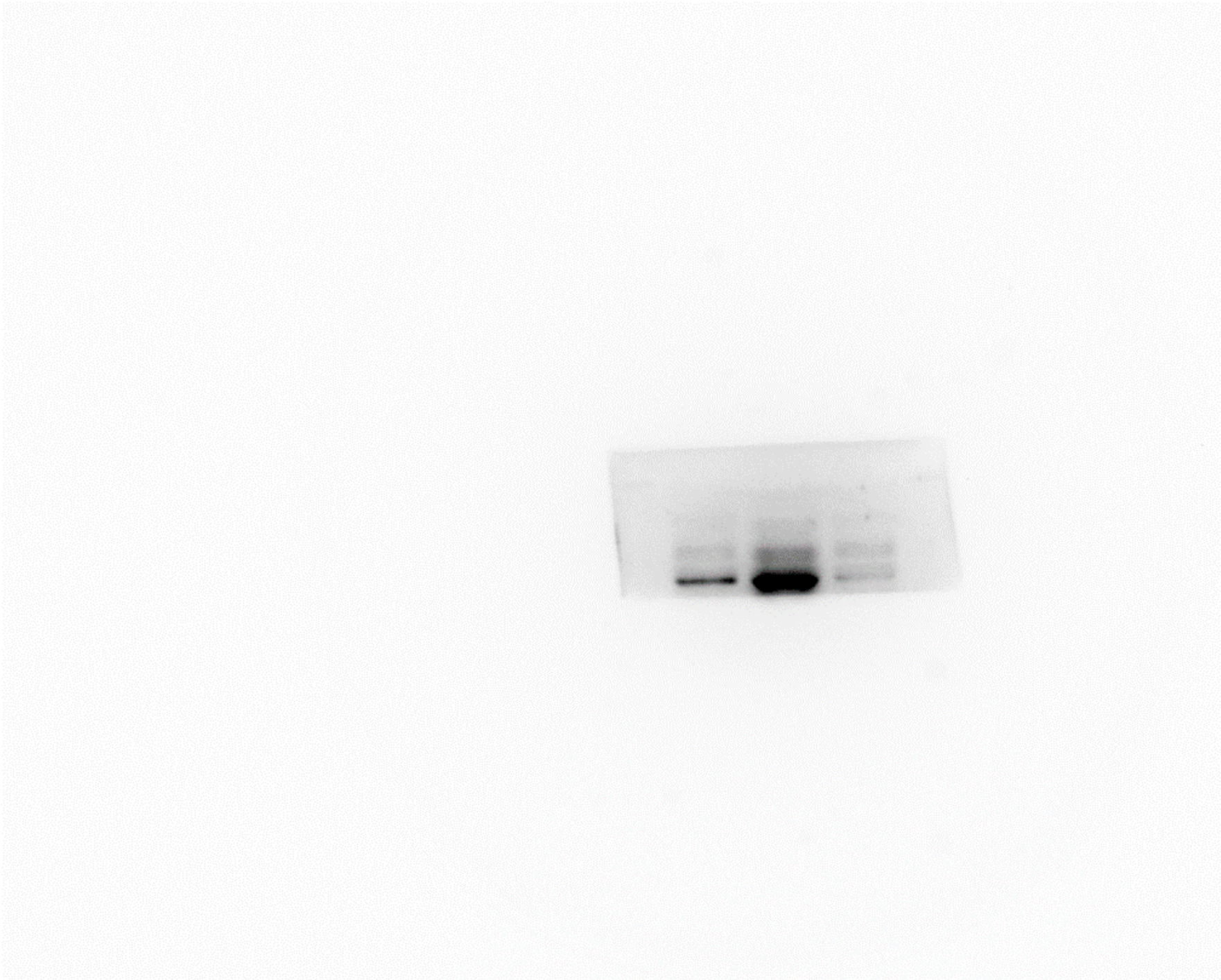

GAPDH in the cell (CON group， NaCl group，NaCl+CCCP group)

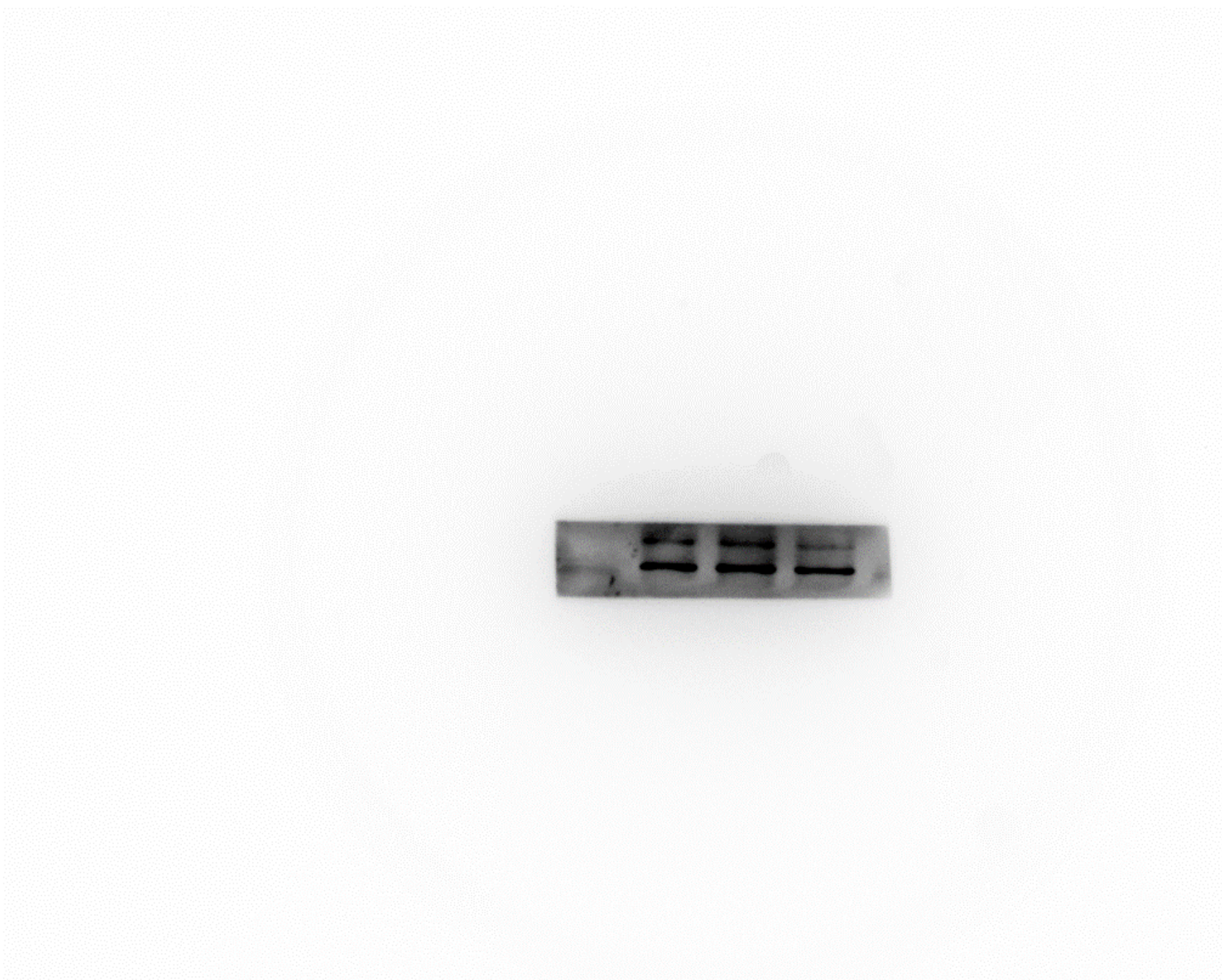

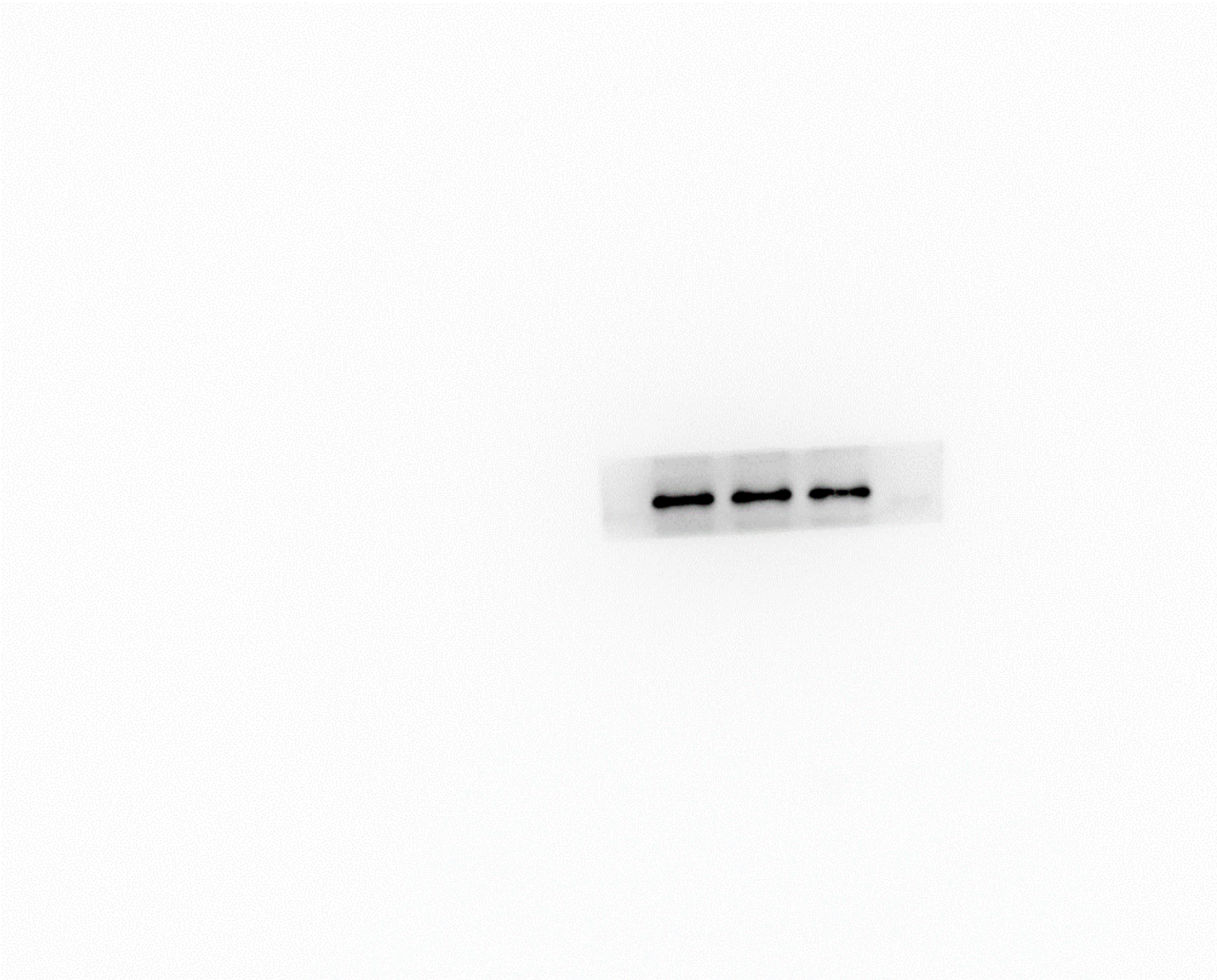

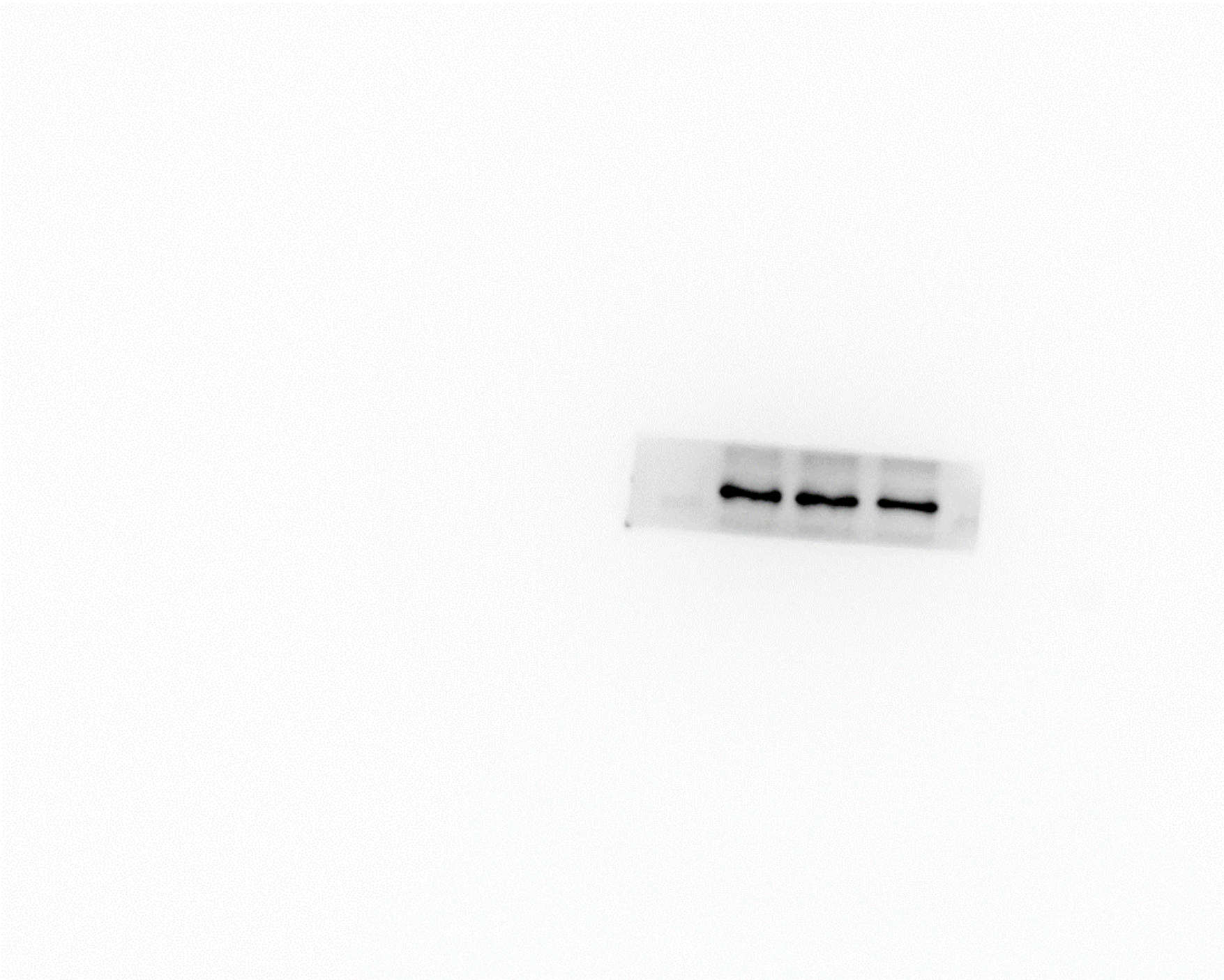

Supplement: Supplementary file 1 [file Data_Sheet_1.PDF]
